# Supplementary material for: Genomic and phenotypic signatures provide insights into the wide adaptation of a global plant invader
Source: Plant Commun. 2024 Jan 13;5(4):100820. doi: 10.1016/j.xplc.2024.100820 (PMC11009367; doi:10.1016/j.xplc.2024.100820)
Supplement: Document S1. Supplemental Figures 1–17 and Supplemental Tables 1–3 [file mmc1.pdf]

Plant Communications, Volume *5*

Supplemental information

Genomic and phenotypic signatures provide insights into the wide adaptation of a global plant invader

Yan Hao, Xin-Feng Wang, Yaolin Guo, Tian-Yang Li, Ji Yang, Malika L. Ainouche, Armel Salmon, Rui-Ting Ju, Ji-Hua Wu, Lin-Feng Li, and Bo Li

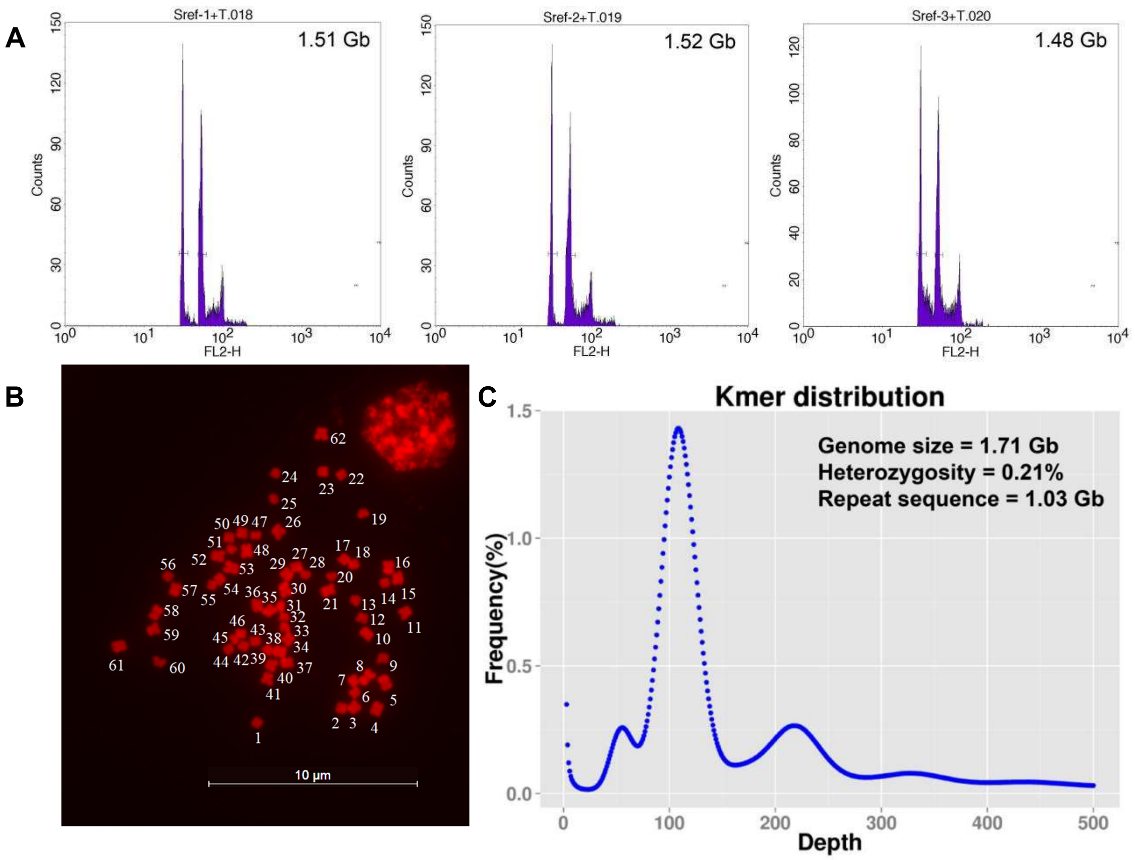

Supplementary Figure 1. (**A**) Genome size of *S. alterniflora* estimated by flow cytometry. Samples from left (1.51 Gb), middle (1.52 Gb) to right (1.48 Gb) are three biological replicates. (**B**) Karyotype of the US sample used to assemble the reference genome. A total of 62 chromosomes were identified. (**C**) K-mer (k = 19) analysis of the *S. alterniflora* genome based on Illumina short reads. A total of 218.01 Gb short reads were used to perform K-mer analysis. The heterozygosity rate is 0.21%.

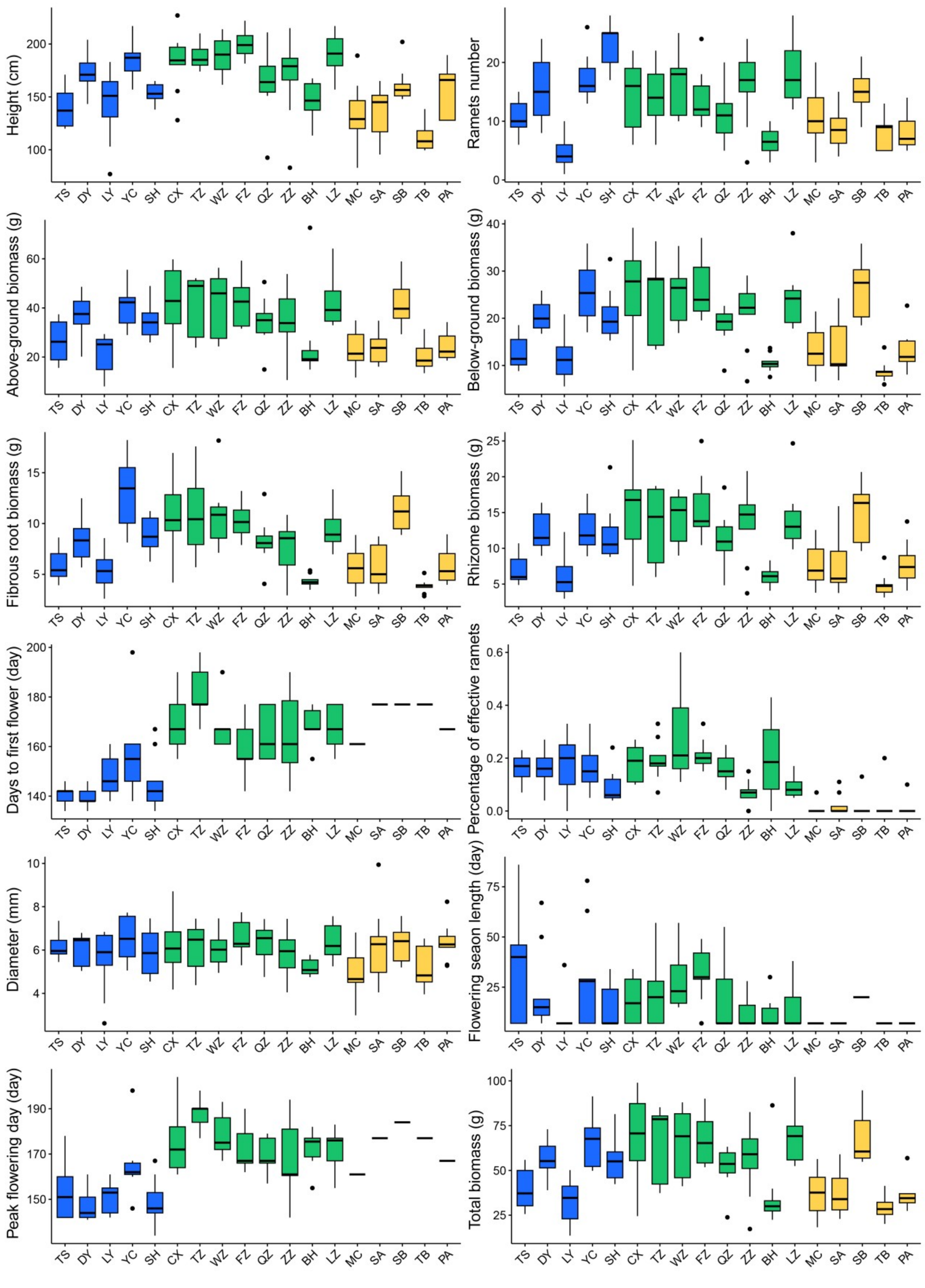

Supplementary Figure 2. Vegetative and reproductive traits of invasive Chinese and native US populations. All plants were planted in plastic pots (16 cm in diameter and 17.5 cm in height) containing a mixture of vermiculite and soil (1:3). All these selected seedlings were grown in greenhouse with 10‰ salinity concentration.

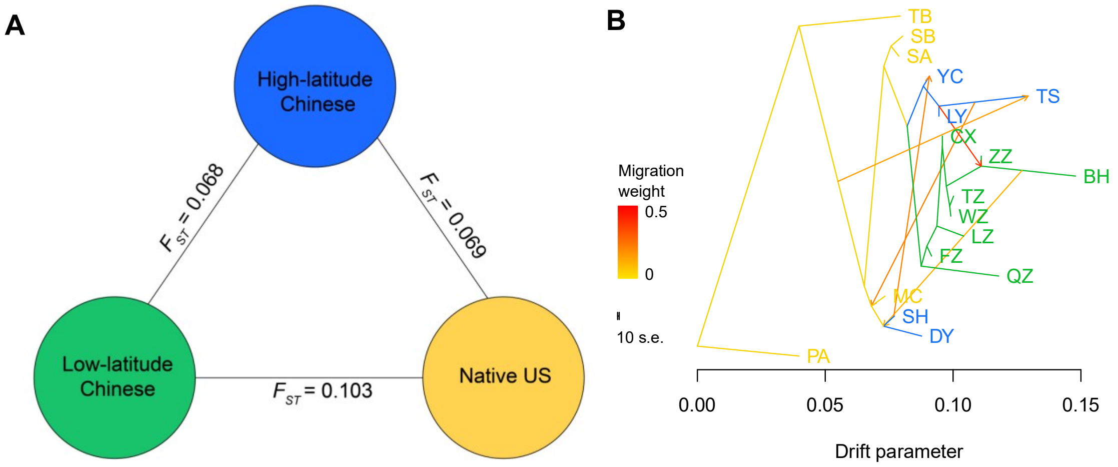

Supplementary Figure 3. (**A**) Genetic differentiation (F_{ST}) at the whole genome level among native US, high- and low-latitude Chinese populations. (**B**) Population split and mixture events inferred based on whole genome SNP dataset. Yellow, blue and green colors represent native US, high-latitude Chinese and low-latitude Chinese populations, respectively. Colored arrows indicate gene flow events among these native and invasive smooth cordgrass populations. Colors from orange to red indicate migration weight from weak to strong.

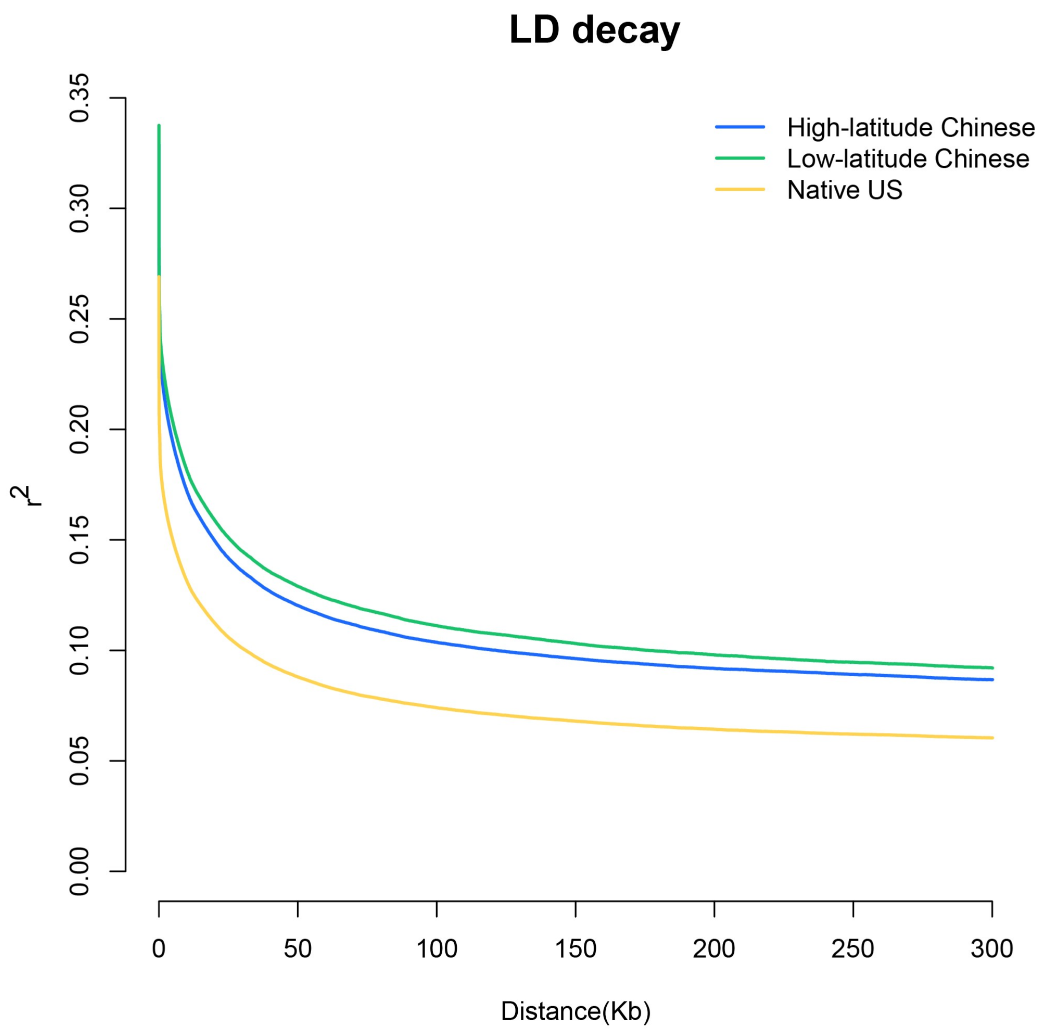

Supplementary Figure 4. Linkage disequilibrium decay of the native US, high- and low-latitude Chinese populations based on whole genome SNP dataset.

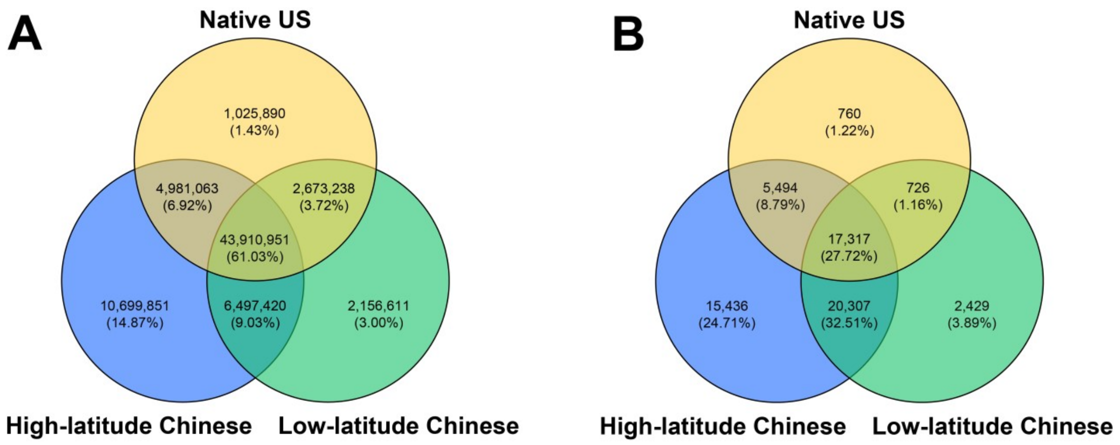

Supplementary Figure 5. Venn analysis of the specific- and shared-SNPs (**A**) and intersecting genes (**B**) among native US, low- and high-latitude Chinese populations. The intersecting genes are those that contain SNPs at gene body region.

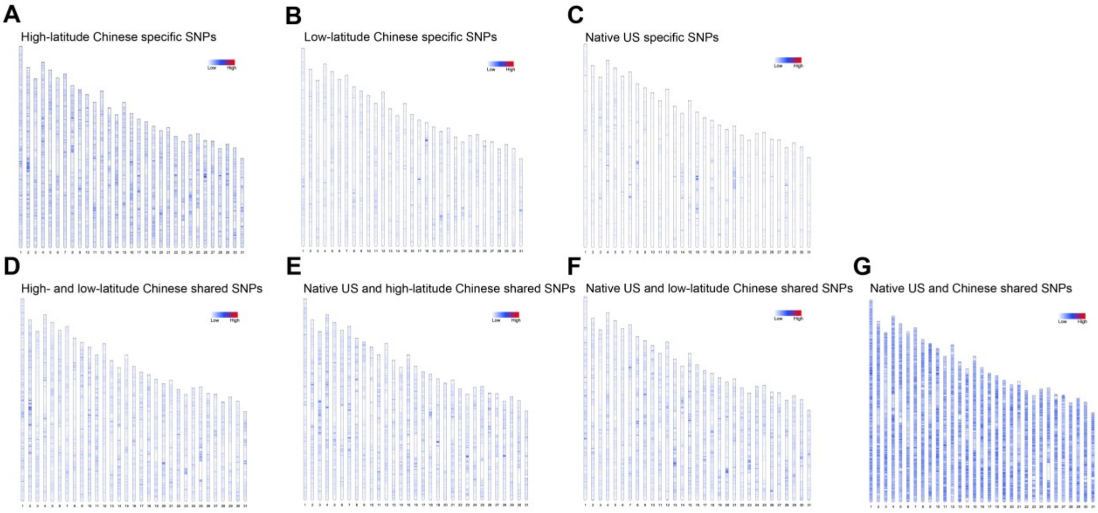

Supplementary Figure 6. Distribution patterns of the specific- and shared-SNPs on the 31 chromosomes. Colors from white to blue and red represent the numbers of SNPs for each 50-Kb sliding window. From subpanels (**A**) to (**G**) are the seven types of SNPs identified in Supplementary Figure 5A.

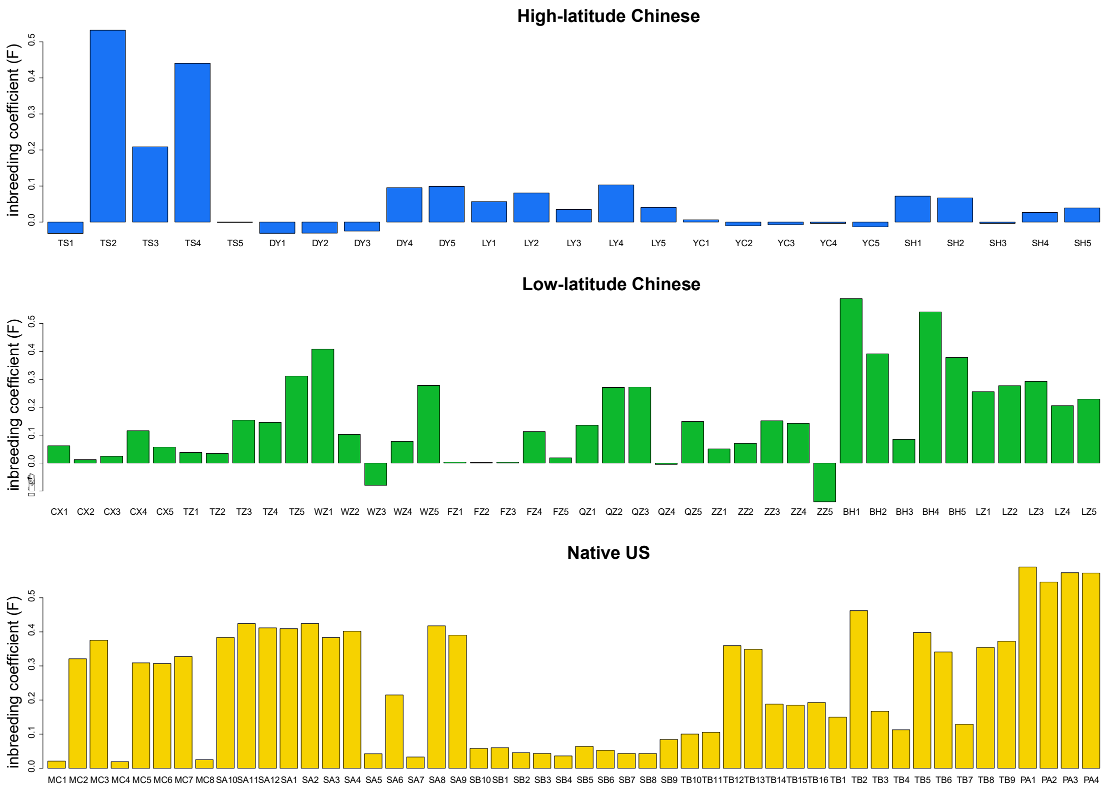

Supplementary Figure 7. Inbreeding coefficient of all native US and invasive Chinese accessions based on whole genome SNP dataset. Each bar on x axes represents an accession. Values on y axes are Inbreeding coefficient (Fis) for each accession.

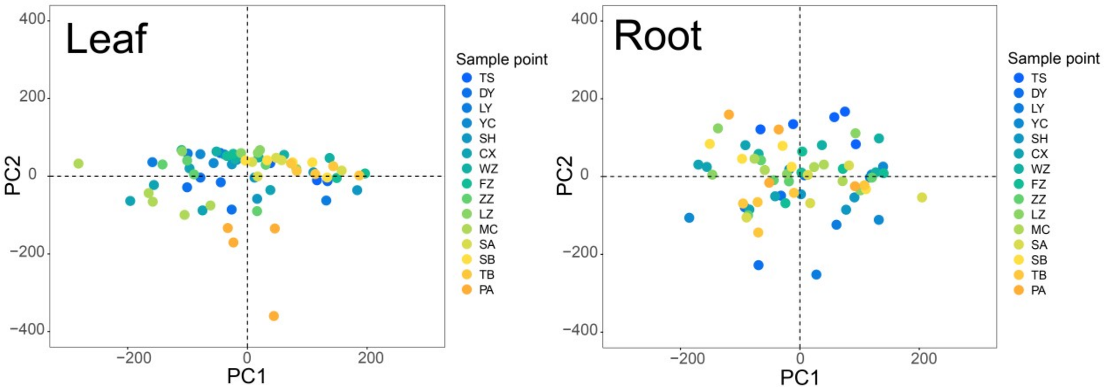

Supplementary Figure 8. Principal component analysis of the overall genes in leaf (on left) and root (on right) tissues, respectively. Different colors represent US and Chinese populations.

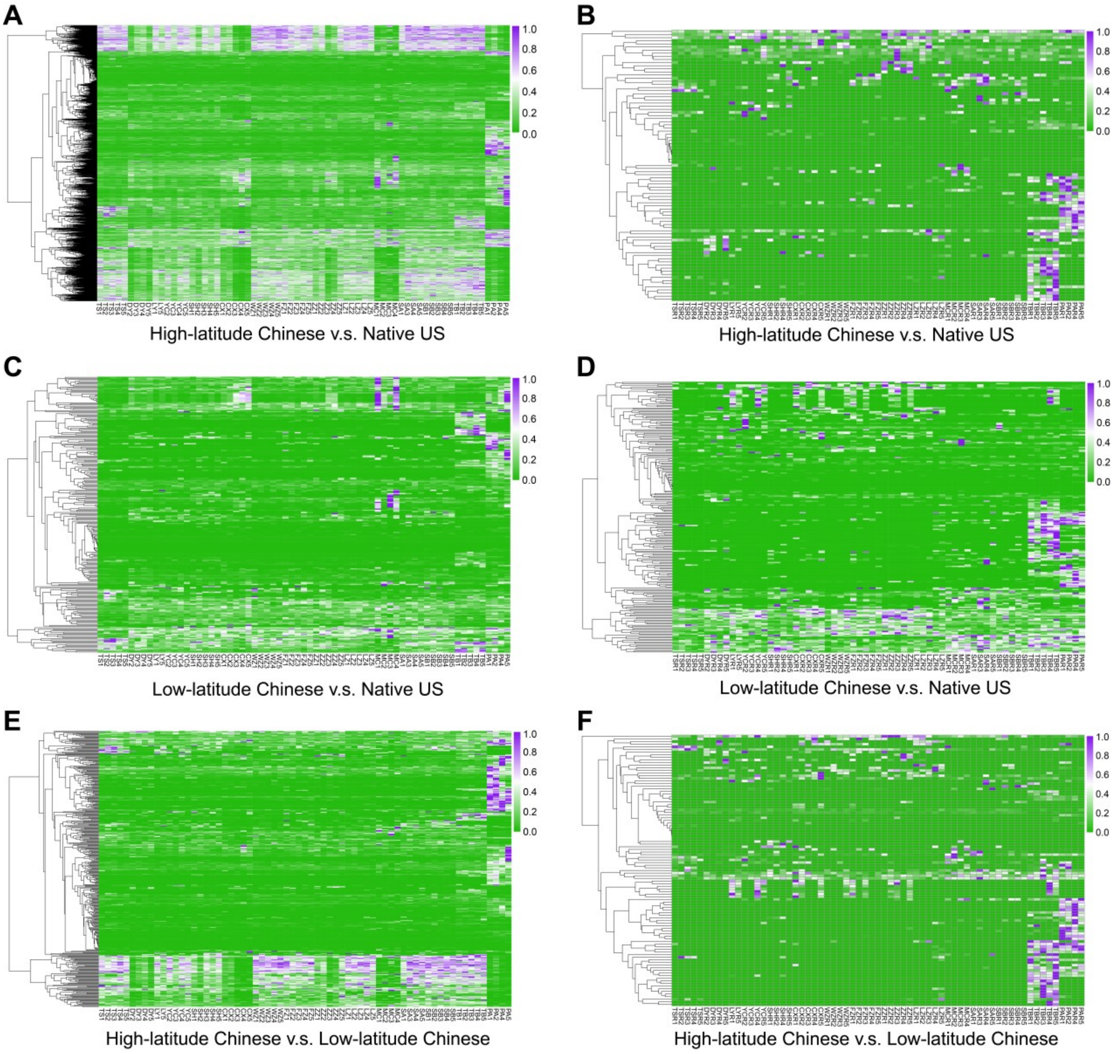

Supplementary Figure 9. Heatmap of differentially expressed genes (DEGs) identified between all invasive Chinese and native US accessions in leaf and root tissues, respectively. (**A**-**B**) Expression pattern of the DEGs identified between native US and high-latitude Chinese populations in leaf and root tissues, respectively. (**C**-**D**) Expression pattern of the DEGs identified between native US and low-latitude Chinese populations in leaf and root tissues, respectively. (**E**-**F**) Expression pattern of the DEGs identified between low- and high-latitude Chinese populations in leaf and root tissues, respectively.

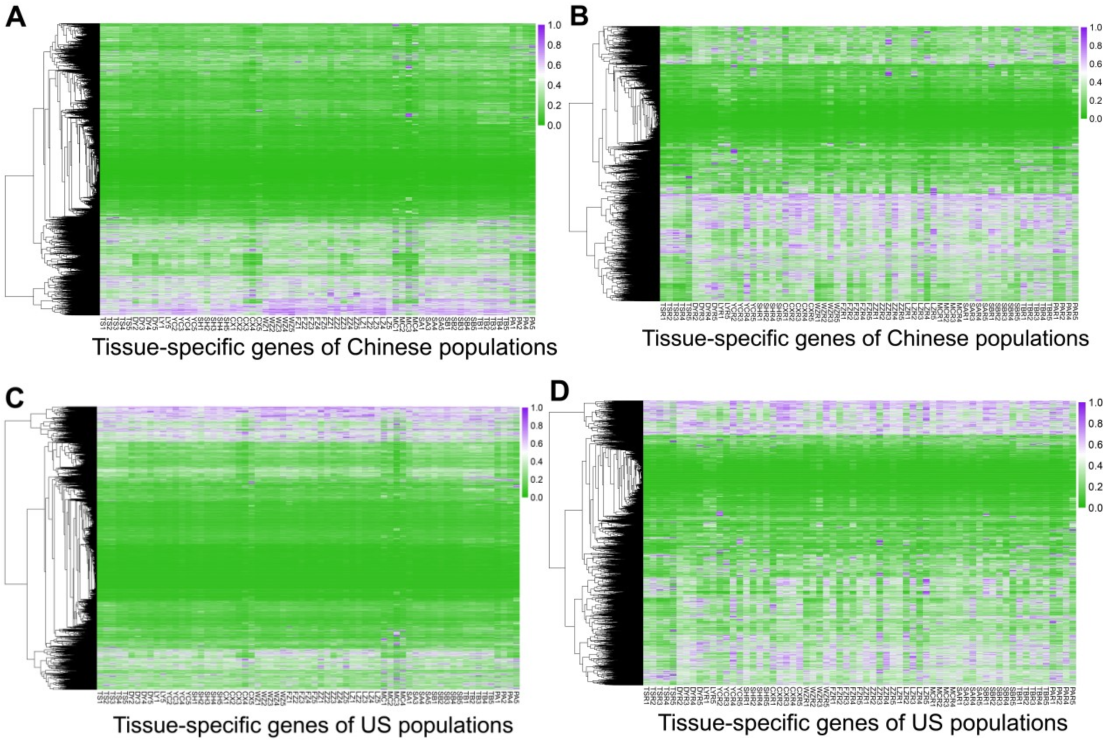

Supplementary Figure 10. Expression pattern of the tissue-specific genes identified between root and leaf tissues. (**A**-**B**) Tissue-specific genes identified in root and leaf tissues of Chinese populations, respectively. (**C**-**D**) Tissue-specific genes identified in root and leaf tissues of US populations, respectively.

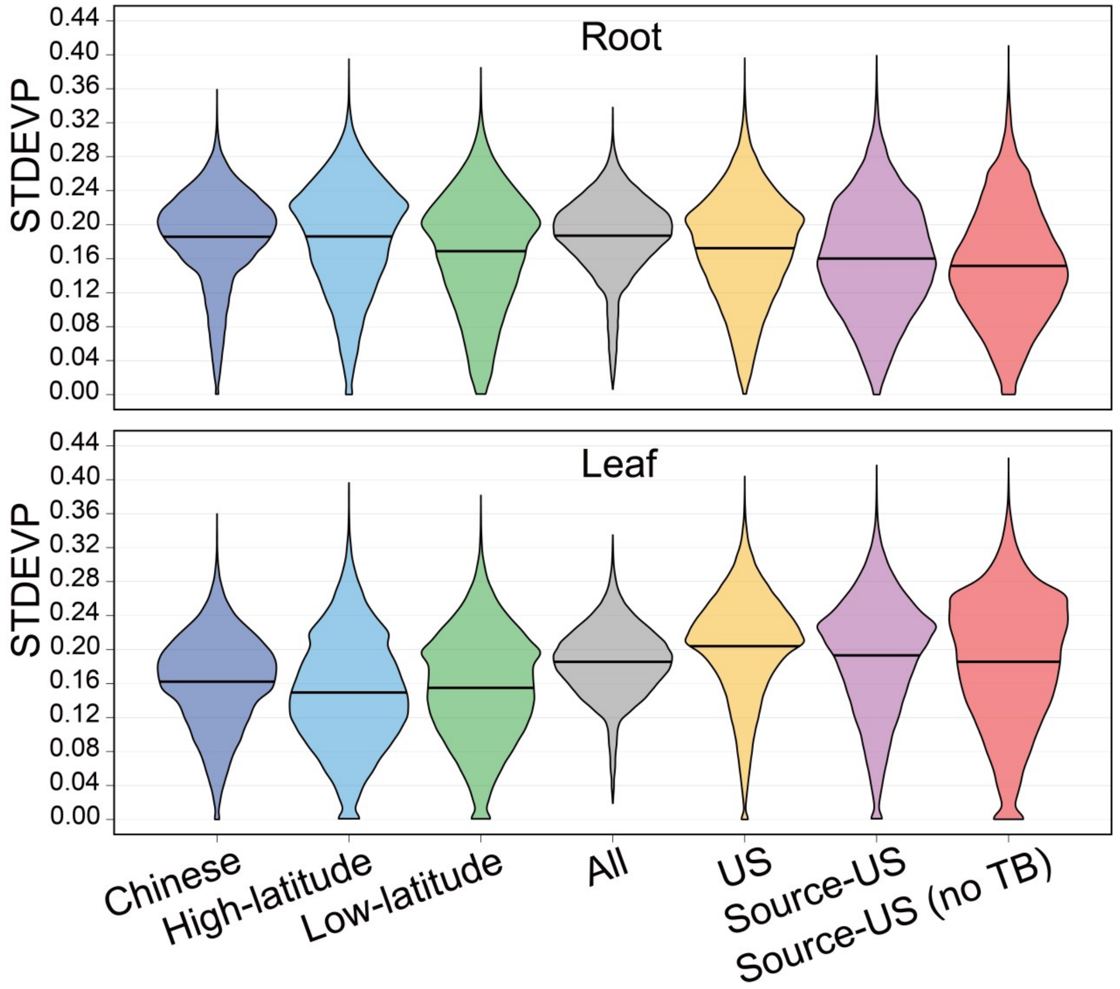

Supplementary Figure 11. Gene expression divergence in leaf and root tissues of the US and Chinese groups. Each colored violin represents a group of the US and Chinese populations. The black line in the violin is the mean value. Values on y axes are standard deviation (STDEVP) for each group.

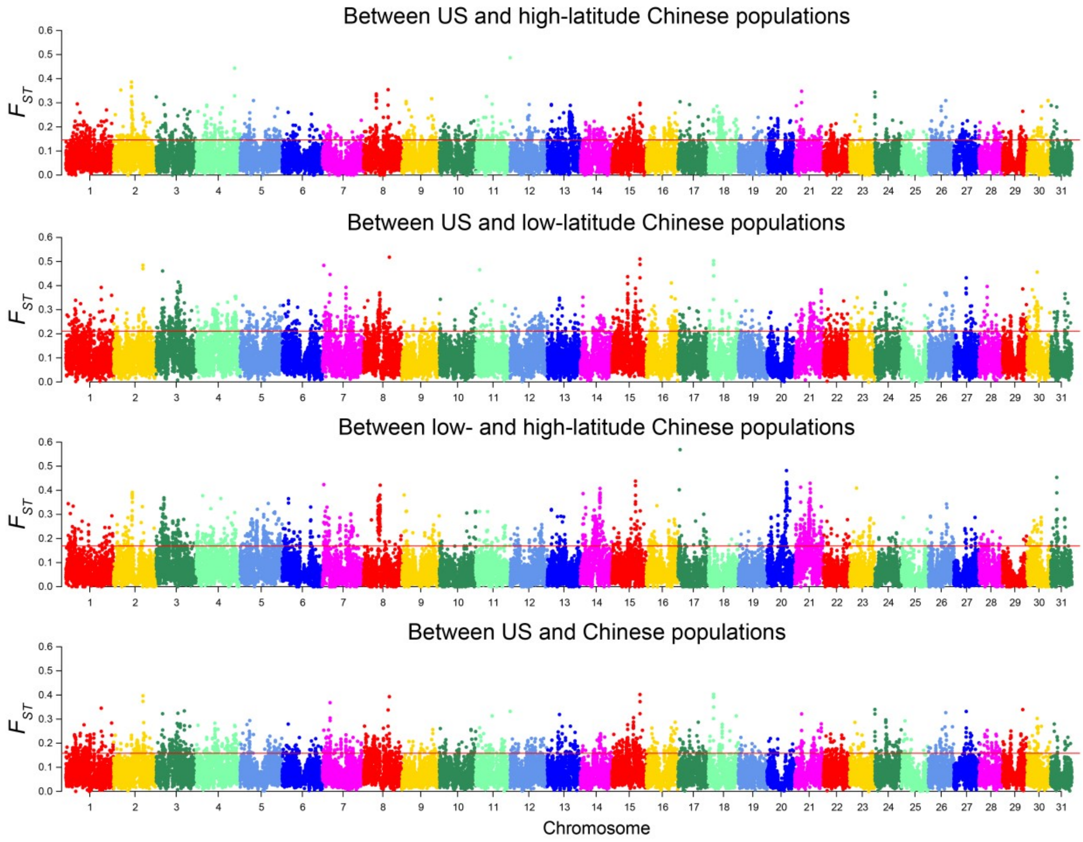

Supplementary Figure 12. Genetic differentiation (F_{ST}) among native US, low- and high-latitude populations of *S. alterniflora*. Each dot represents a 50-Kb genomic region. Dots above the red line is the top 5% highest divergent genomic regions.

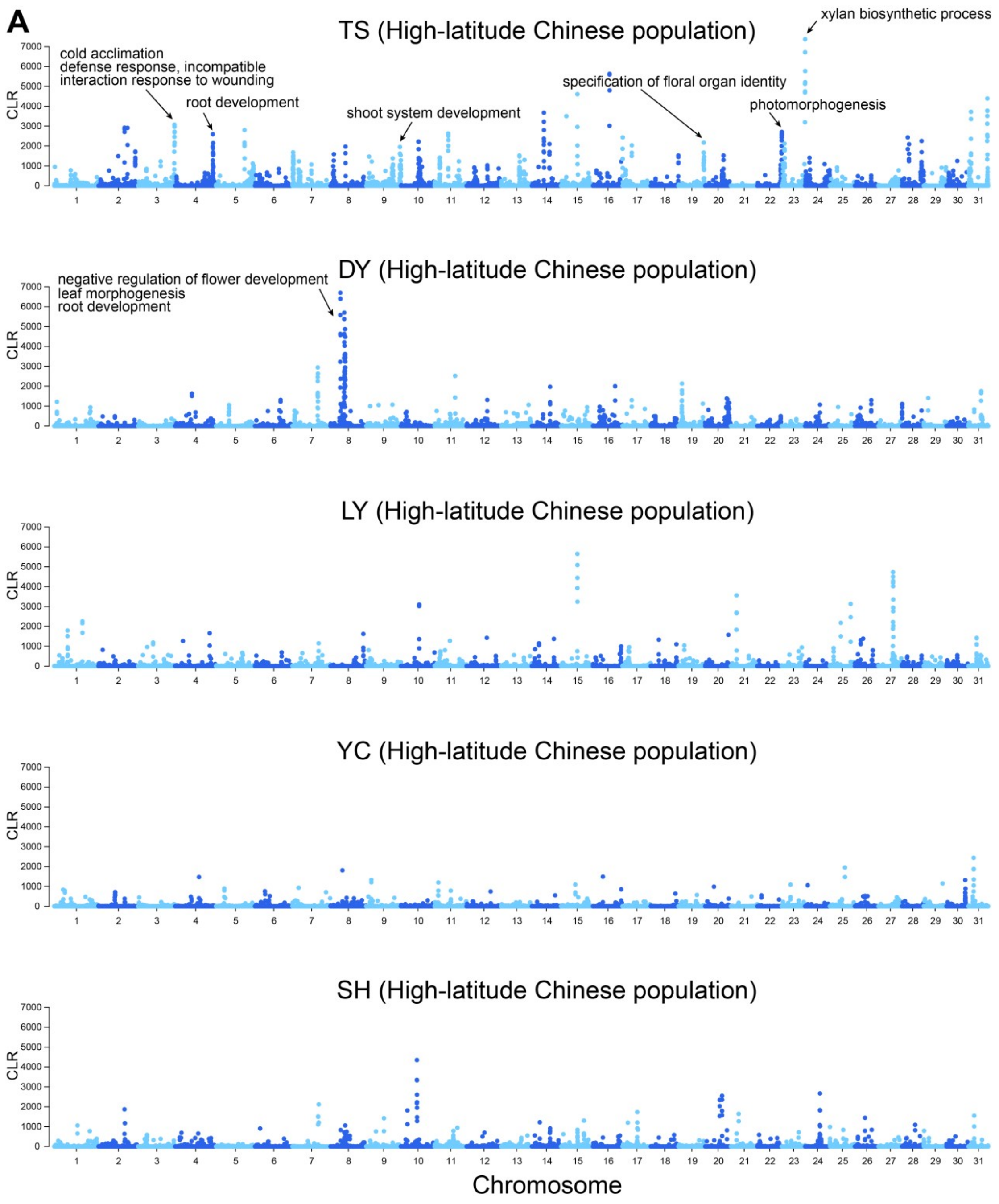
A
TS (High-latitude Chinese population)
cold acclimation
defense response, incompatible interaction response to wounding
root development
shoot system development
specification of floral organ identity
photomorphogenesis
xylan biosynthetic process
DY (High-latitude Chinese population)
negative regulation of flower development
leaf morphogenesis
root development
LY (High-latitude Chinese population)
YC (High-latitude Chinese population)
SH (High-latitude Chinese population)
CLR
Chromosome

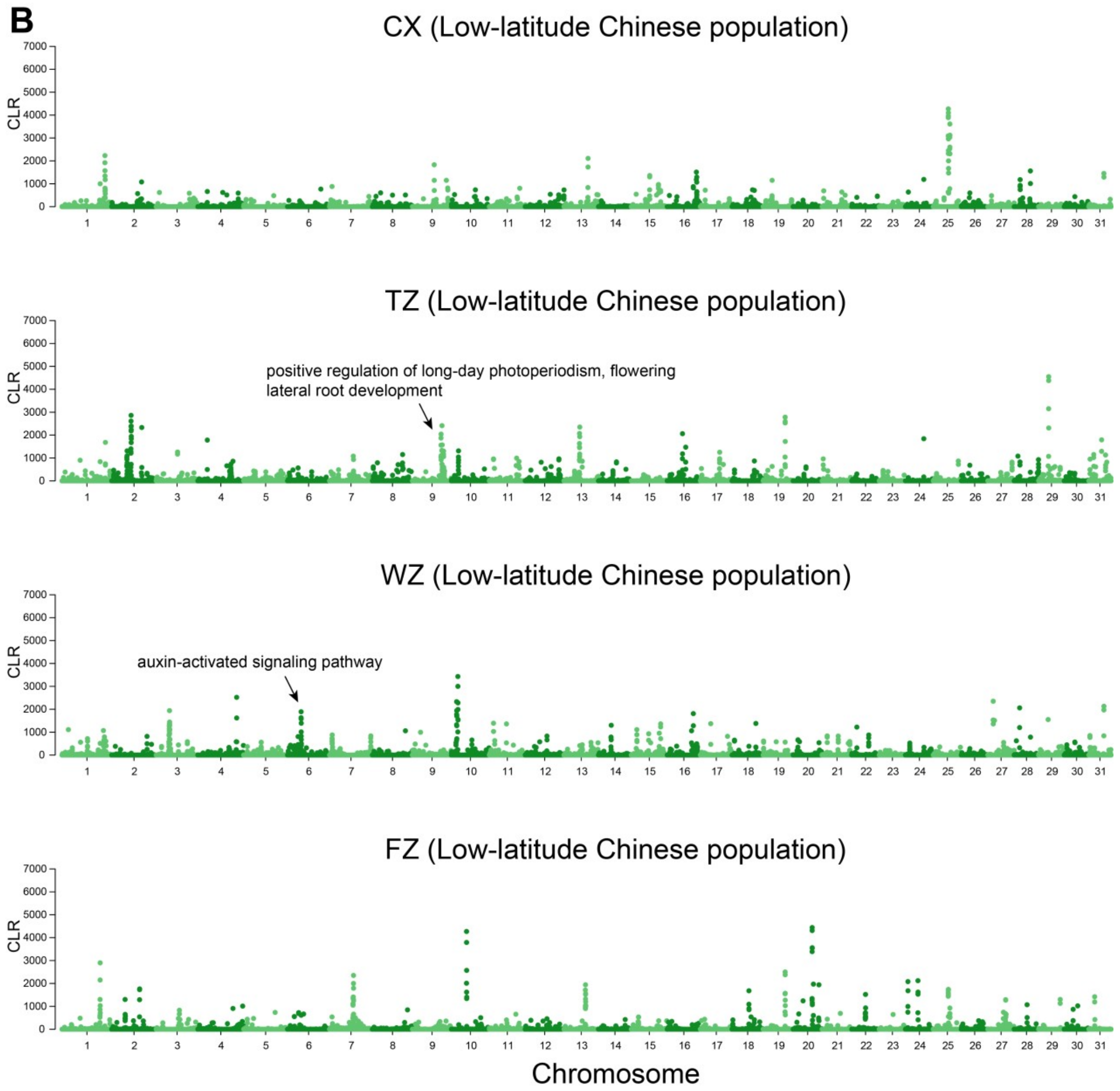
B
CX (Low-latitude Chinese population)
TZ (Low-latitude Chinese population)
positive regulation of long-day photoperiodism, flowering
lateral root development
WZ (Low-latitude Chinese population)
auxin-activated signaling pathway
FZ (Low-latitude Chinese population)
CLR
Chromosome

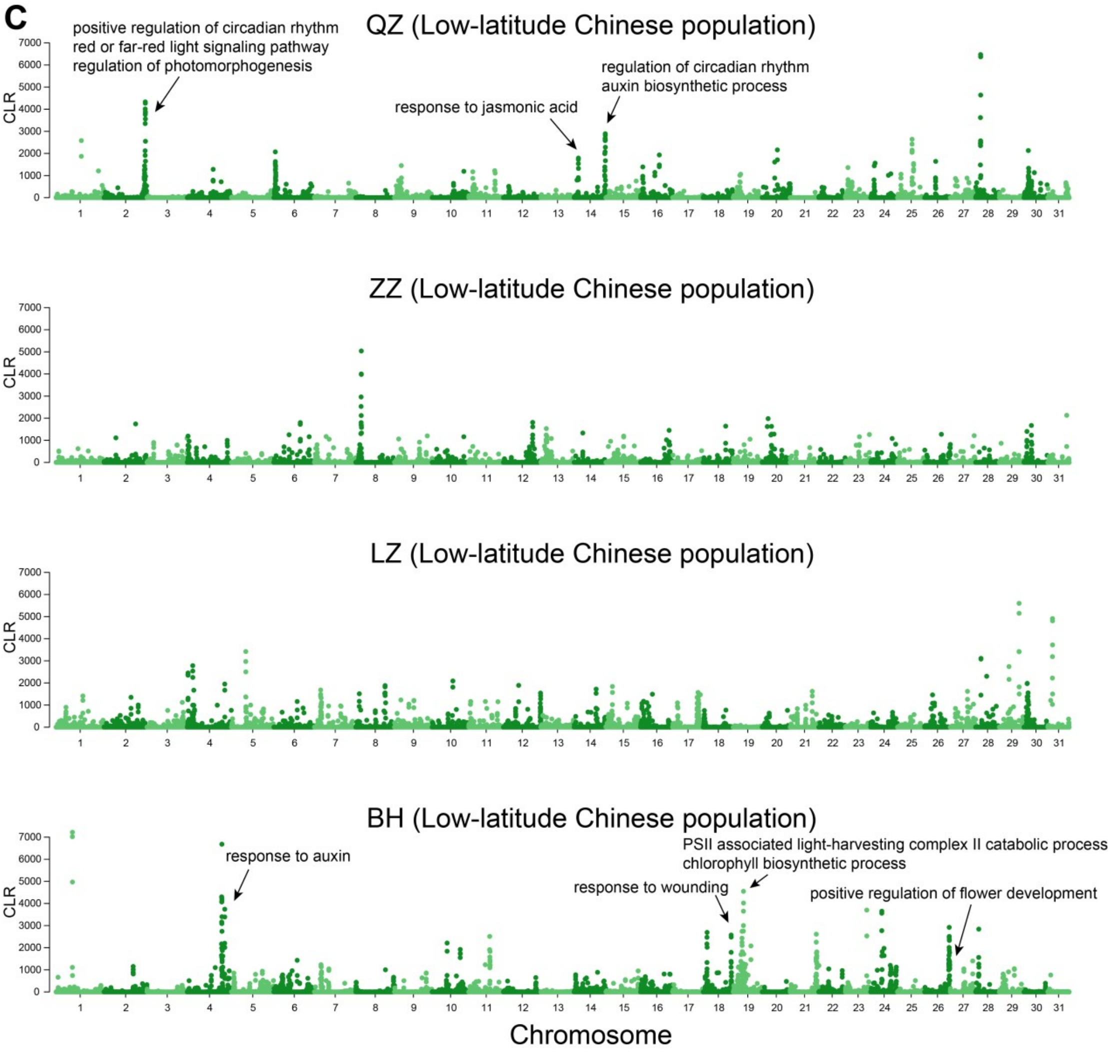
C
QZ (Low-latitude Chinese population)
positive regulation of circadian rhythm
red or far-red light signaling pathway
regulation of photomorphogenesis
response to jasmonic acid
regulation of circadian rhythm
auxin biosynthetic process
ZZ (Low-latitude Chinese population)
LZ (Low-latitude Chinese population)
BH (Low-latitude Chinese population)
response to auxin
PSII associated light-harvesting complex II catabolic process
chlorophyll biosynthetic process
response to wounding
positive regulation of flower development
CLR
Chromosome

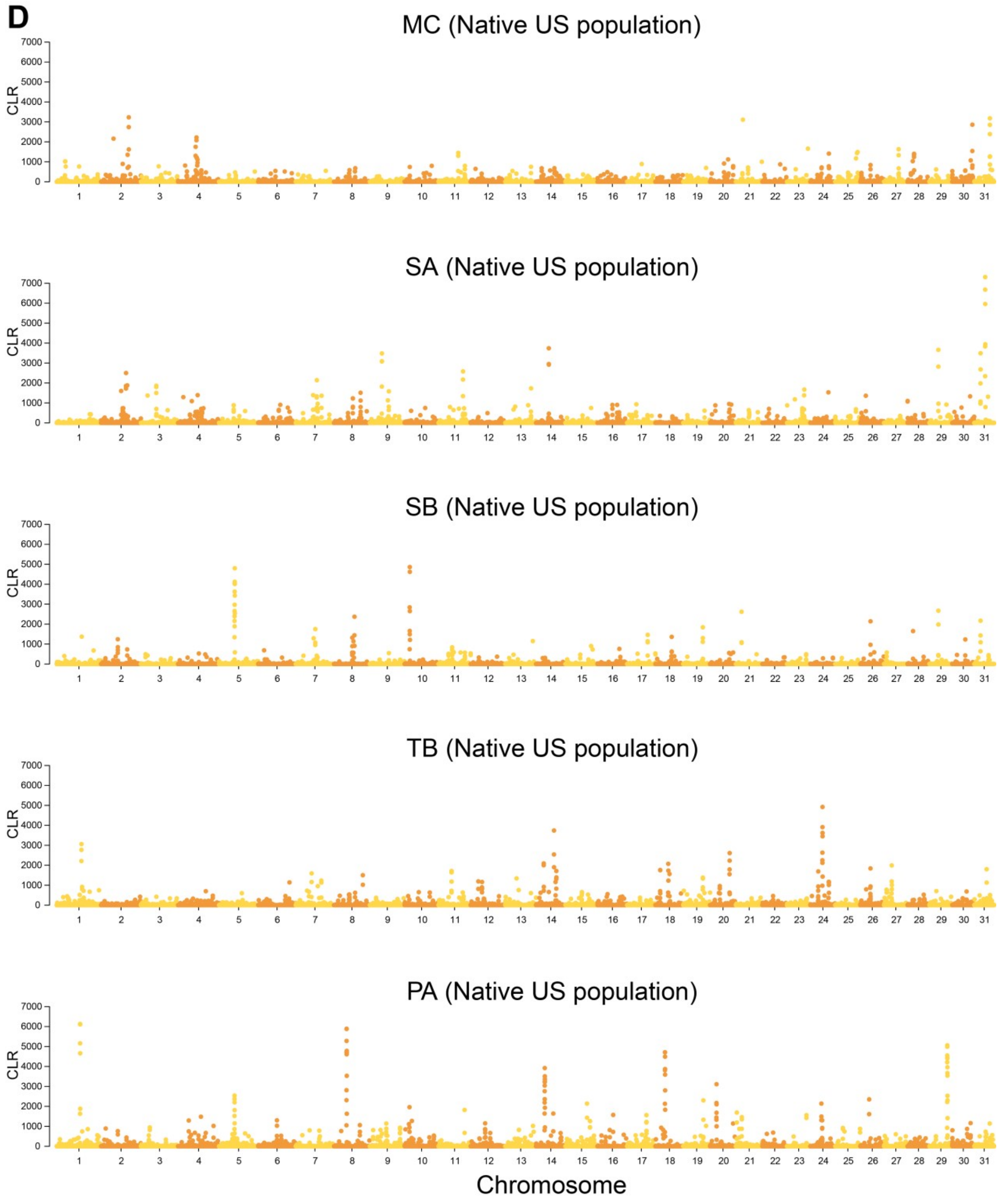

Supplementary Figure 13. Selective genomic regions along the chromosomes of *S. alterniflora* that identified by SweeD in high-latitude Chinese (**A**), low-latitude Chinese (**B**-**C**) and native US populations (**D**). Values on y axes represent the composite likelihood ratio (CLR). Each dot is a 50-Kb genomic region on the chromosome. Population names are the same as Supplementary Table S16.

BIO1: Annual Mean Temperature

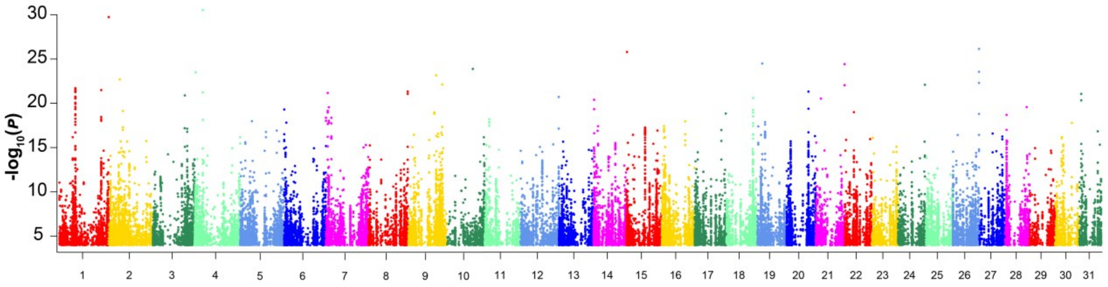

BIO2: Mean Diurnal Range (Mean of monthly (max temp - min temp))

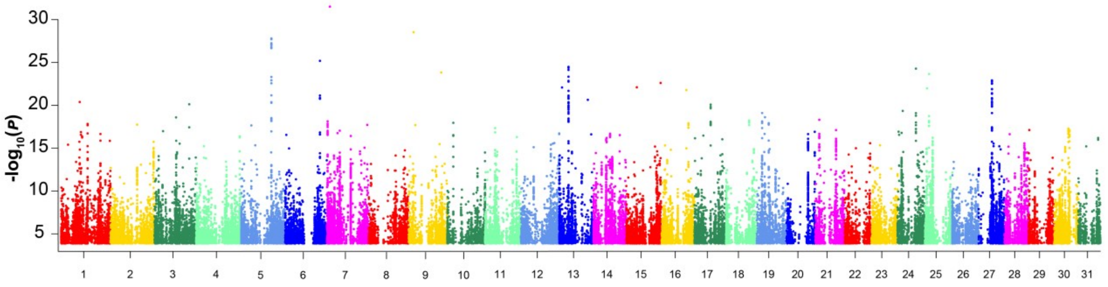

BIO3: Isothermality

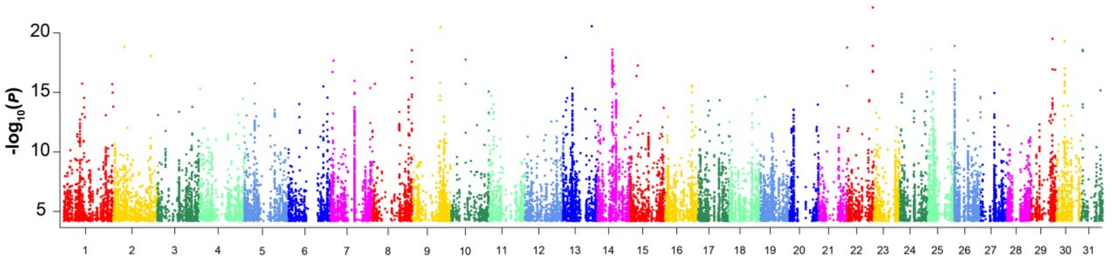

BIO4: Temperature Seasonality (standard deviation ×100)

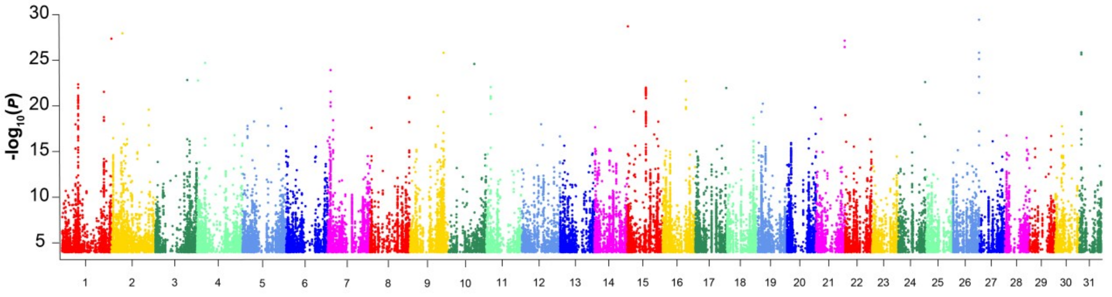

BIO5: Max Temperature of Warmest Month

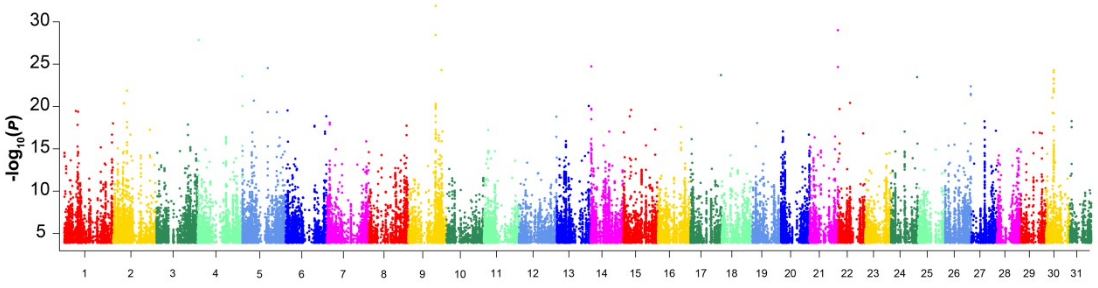

BIO6: Min Temperature of Coldest Month

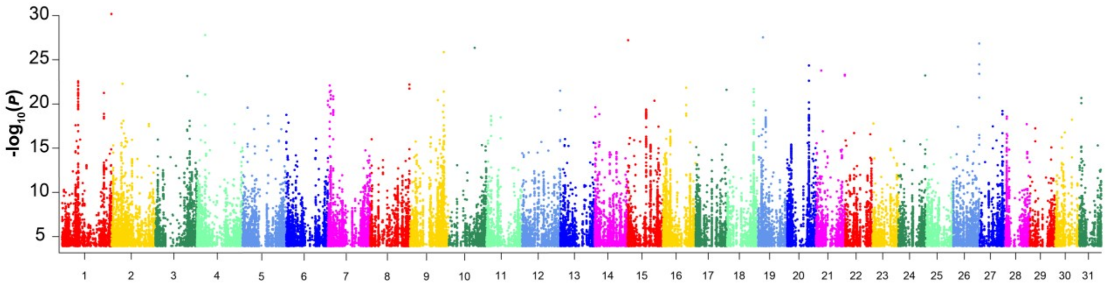

BIO7: Temperature Annual Range

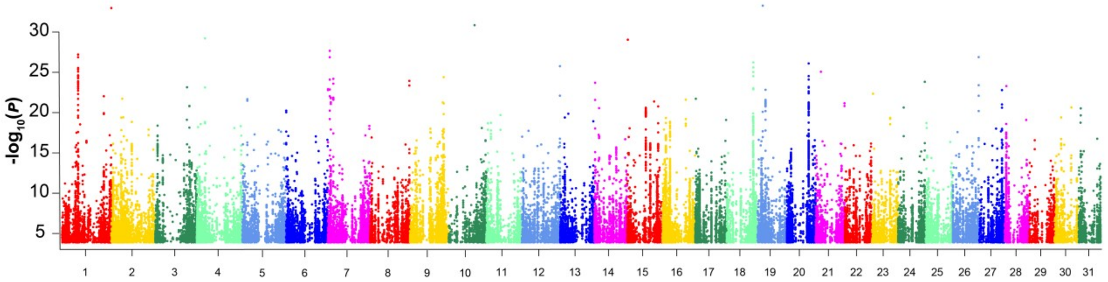

BIO8: Mean Temperature of Wettest Quarter

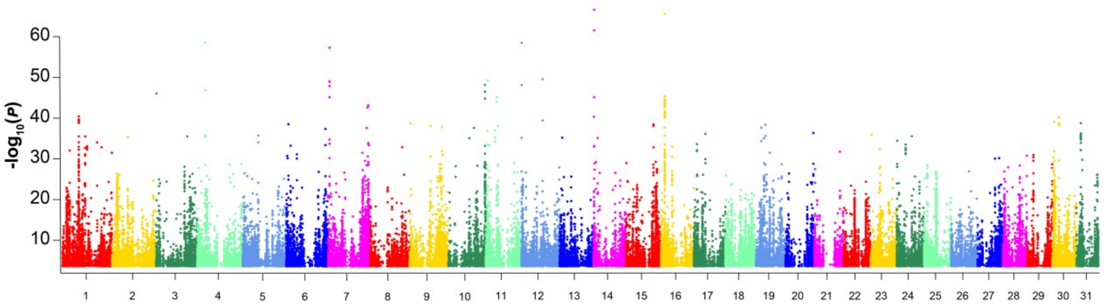

BIO9: Mean Temperature of Driest Quarter

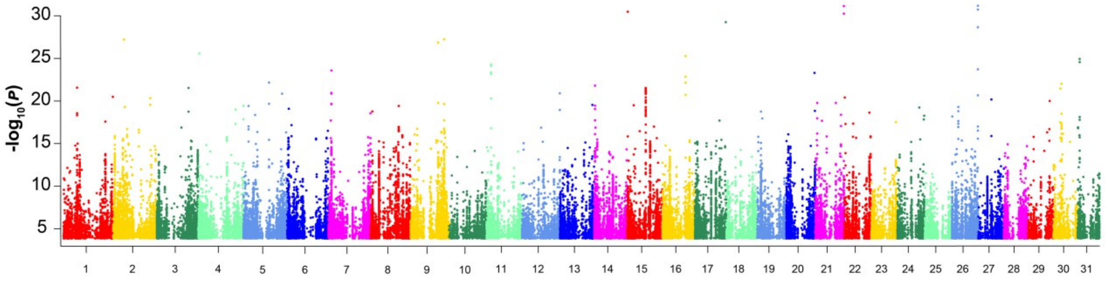

BIO10: Mean Temperature of Warmest Quarter

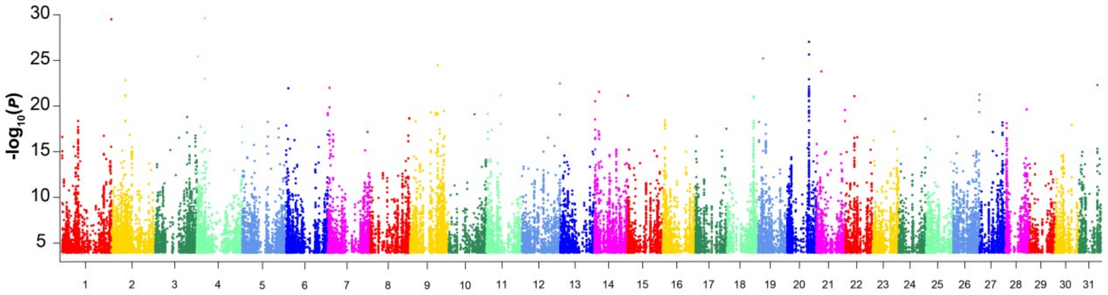

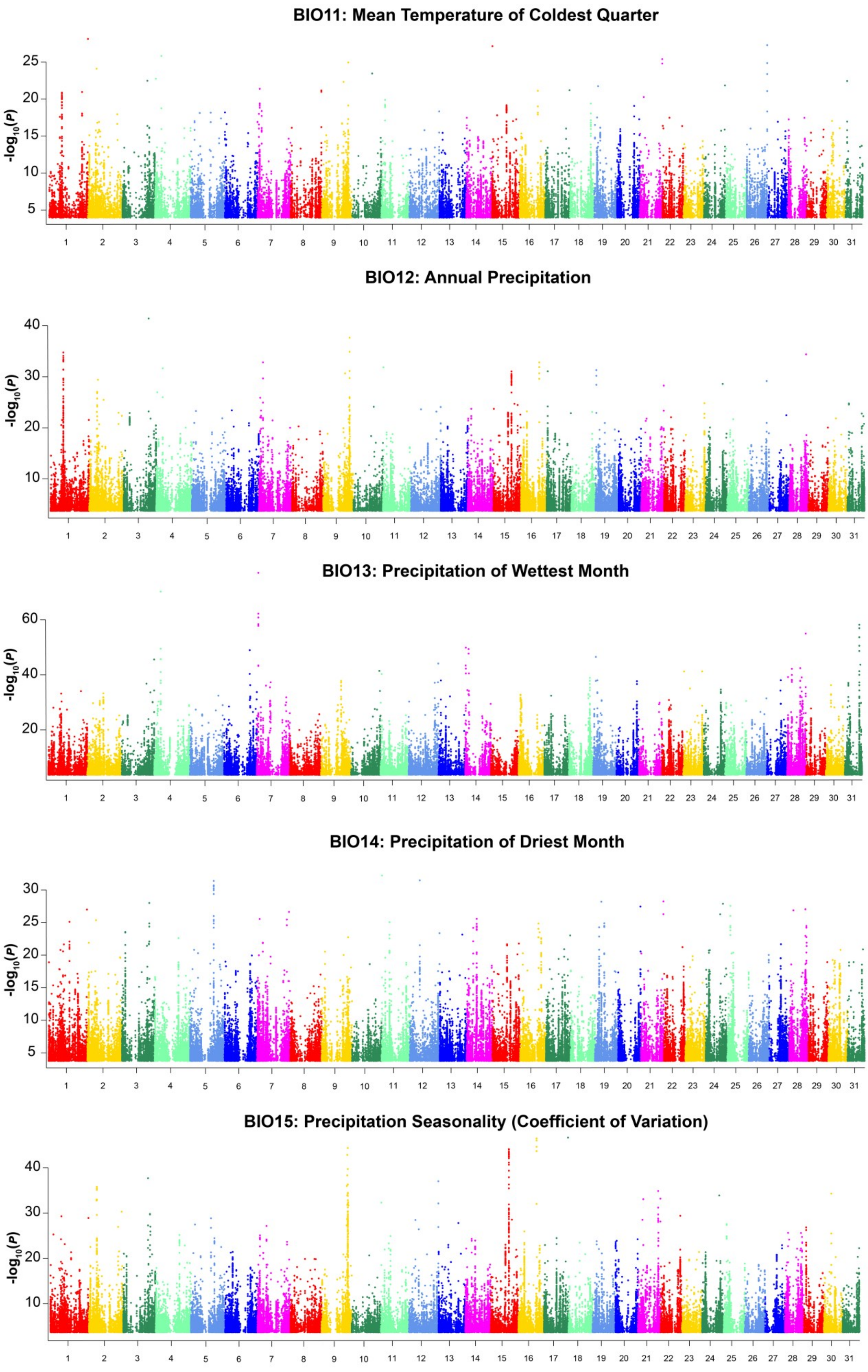
BIO11: Mean Temperature of Coldest Quarter
-log10(P)
BIO12: Annual Precipitation
-log10(P)
BIO13: Precipitation of Wettest Month
-log10(P)
BIO14: Precipitation of Driest Month
-log10(P)
BIO15: Precipitation Seasonality (Coefficient of Variation)
-log10(P)
1 2 3 4 5 6 7 8 9 10 11 12 13 14 15 16 17 18 19 20 21 22 23 24 25 26 27 28 29 30 31

BIO16: Precipitation of Wettest Quarter

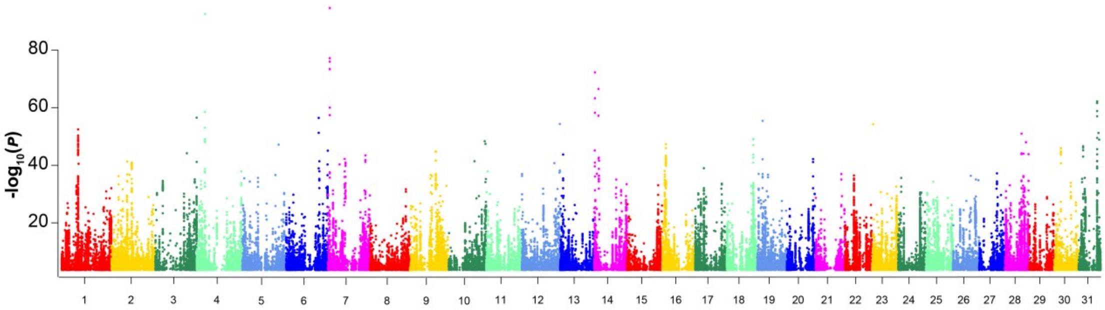

BIO17: Precipitation of Driest Quarter

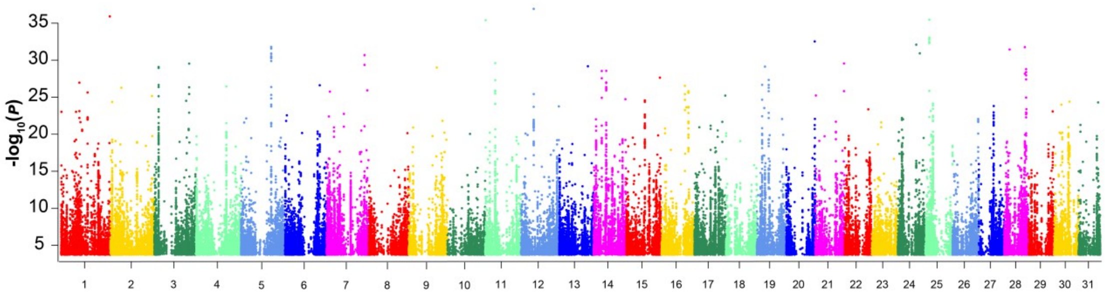

BIO18: Precipitation of Warmest Quarter

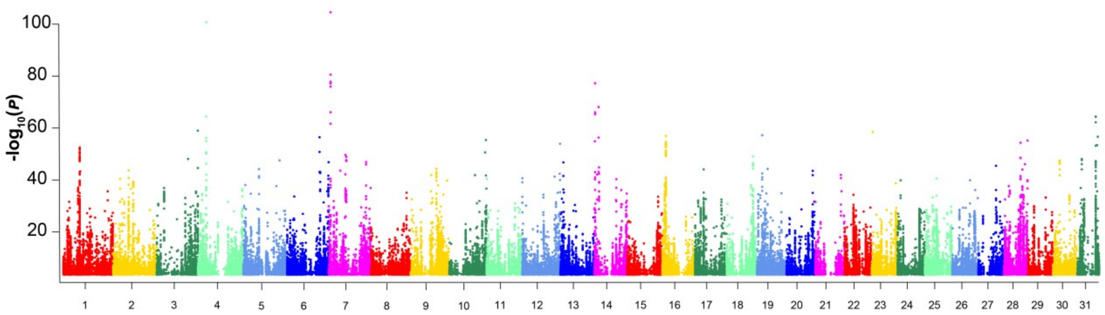

BIO19: Precipitation of Coldest Quarter

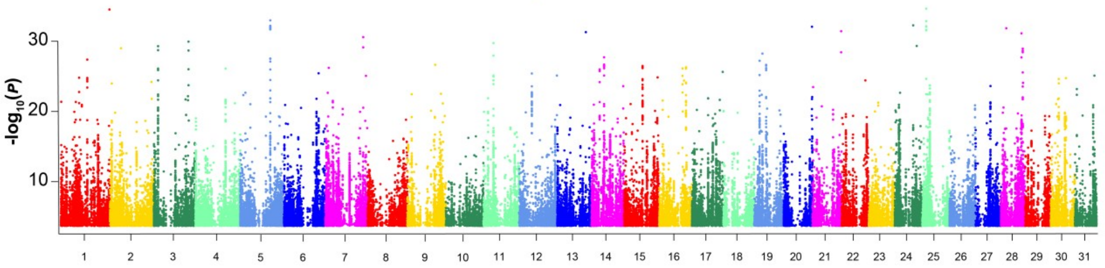

BIO20: Actual Evapotranspiration

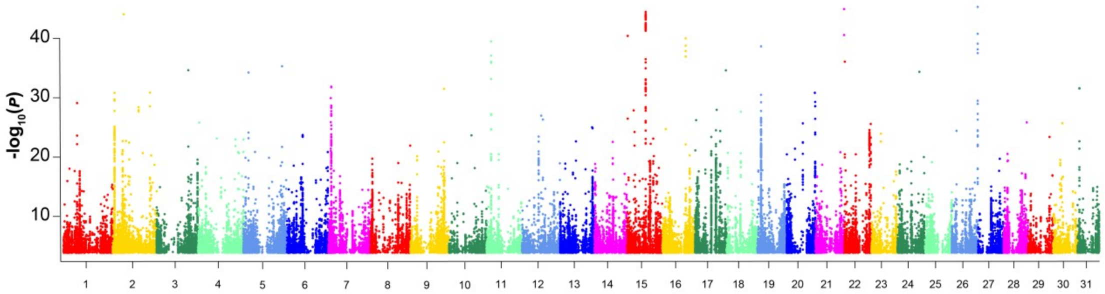

BIO21: Climate Water Deficit

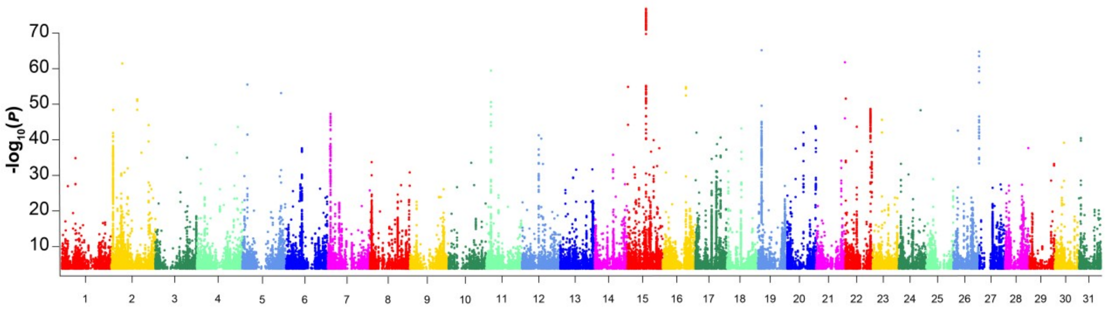

BIO22: Palmer Drought Severity Index

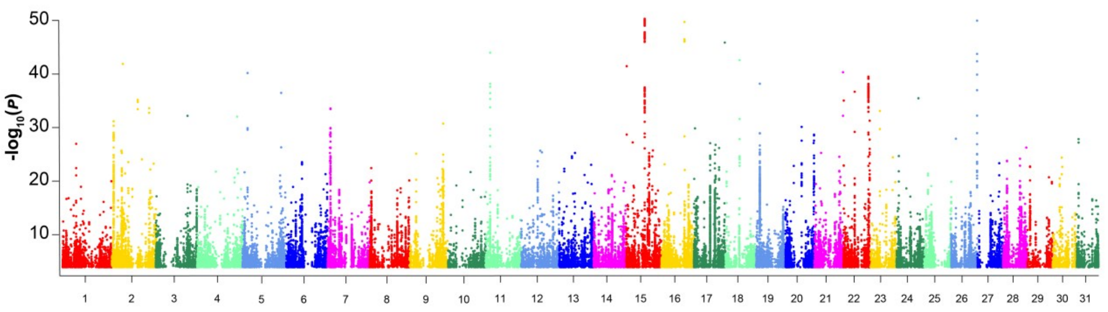

BIO23: Precipitation Accumulation

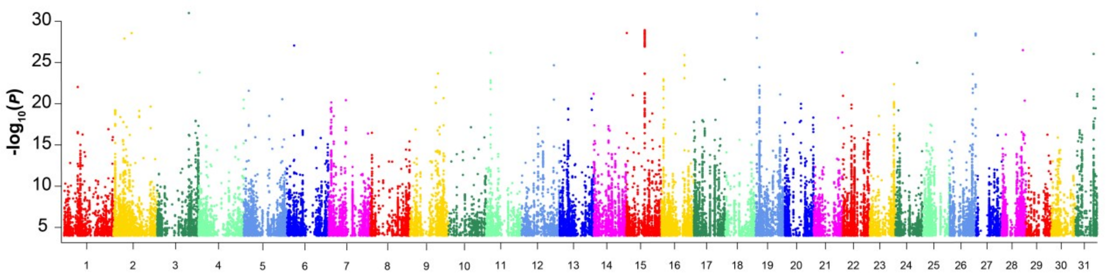

BIO24: Soil Moisture

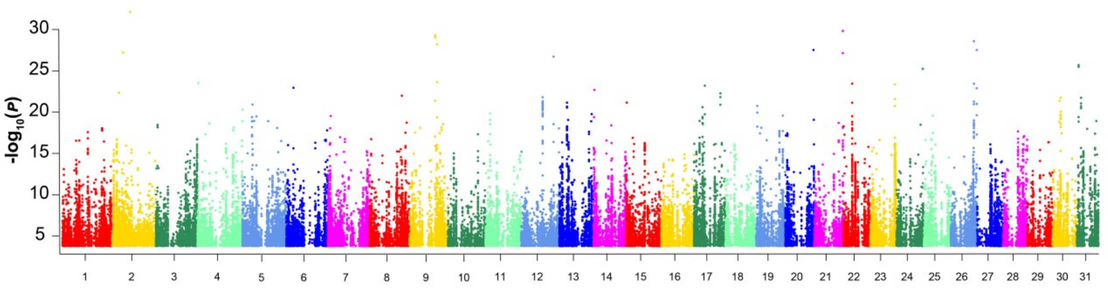

BIO25: Downward Surface Shortwave Radiation

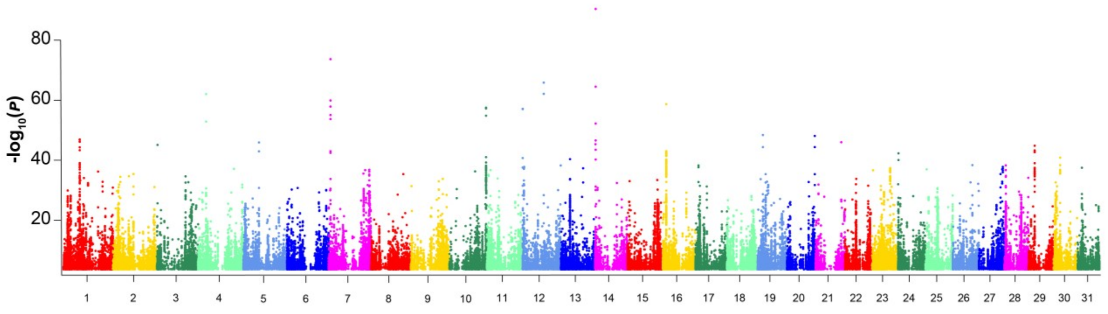

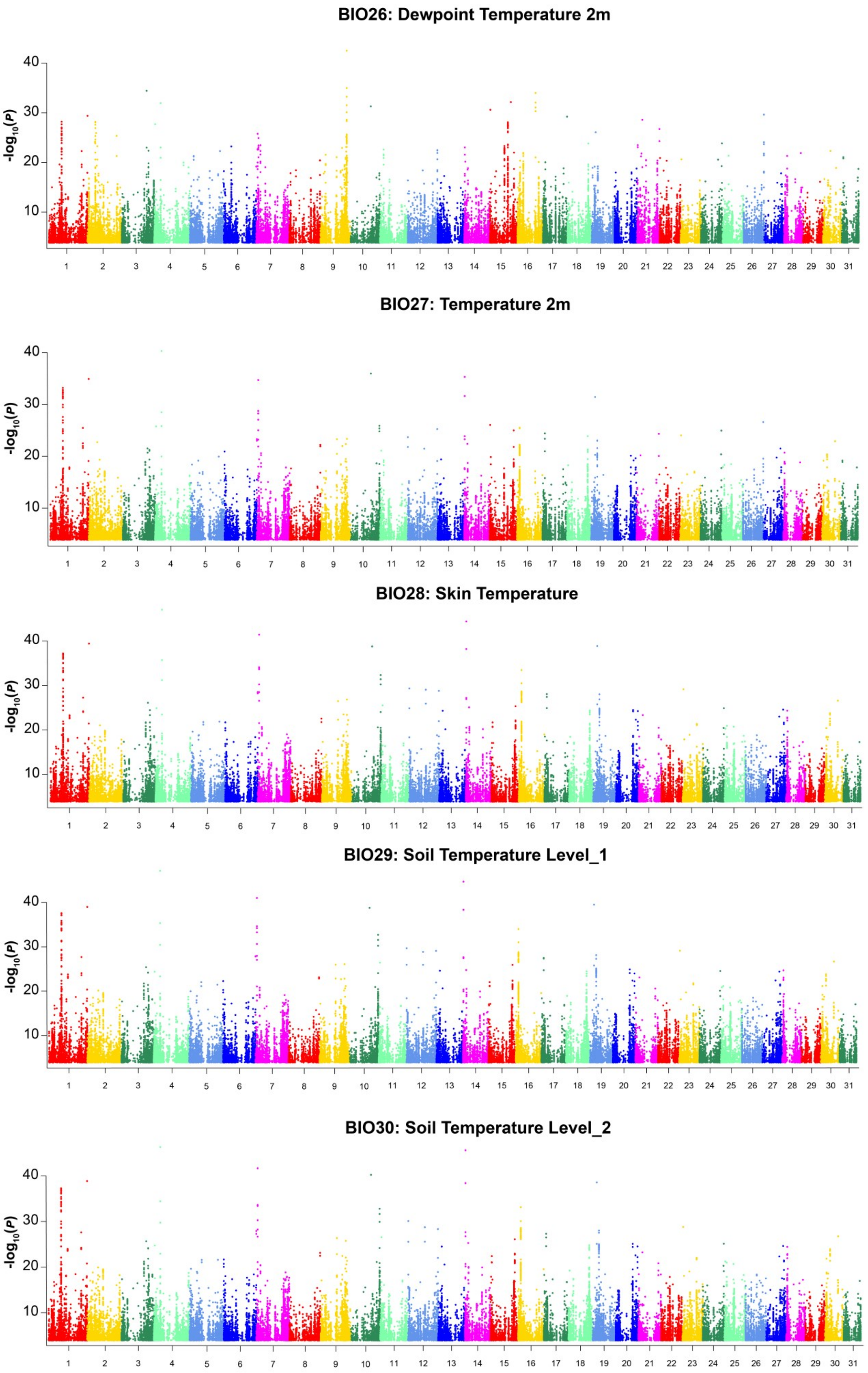
BIO26: Dewpoint Temperature 2m
BIO27: Temperature 2m
BIO28: Skin Temperature
BIO29: Soil Temperature Level_1
BIO30: Soil Temperature Level_2
-log10(P)

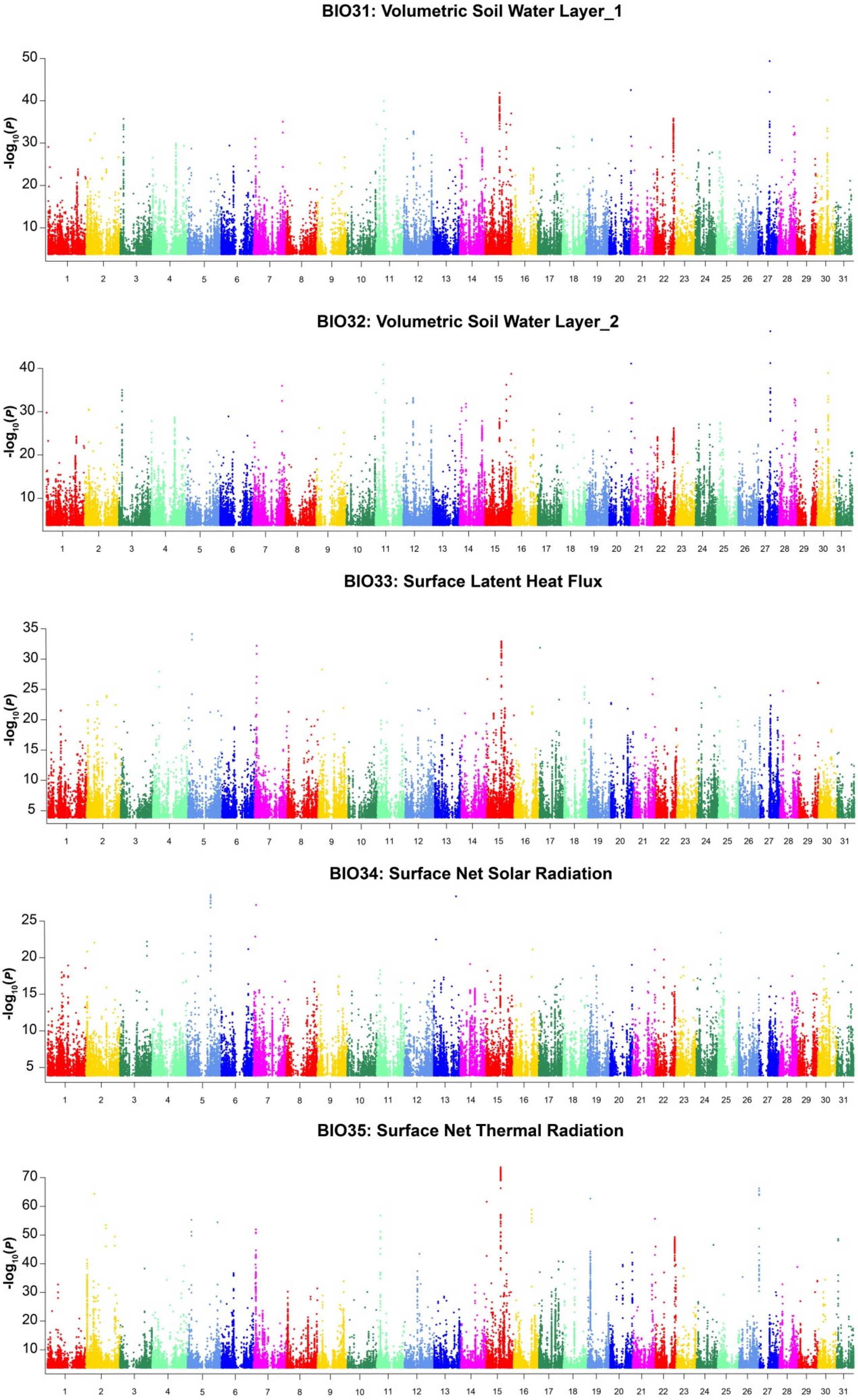
BIO31: Volumetric Soil Water Layer_1
-log10(P)
10 20 30 40 50
1 2 3 4 5 6 7 8 9 10 11 12 13 14 15 16 17 18 19 20 21 22 23 24 25 26 27 28 29 30 31
BIO32: Volumetric Soil Water Layer_2
-log10(P)
10 20 30 40
1 2 3 4 5 6 7 8 9 10 11 12 13 14 15 16 17 18 19 20 21 22 23 24 25 26 27 28 29 30 31
BIO33: Surface Latent Heat Flux
-log10(P)
5 10 15 20 25 30 35
1 2 3 4 5 6 7 8 9 10 11 12 13 14 15 16 17 18 19 20 21 22 23 24 25 26 27 28 29 30 31
BIO34: Surface Net Solar Radiation
-log10(P)
5 10 15 20 25
1 2 3 4 5 6 7 8 9 10 11 12 13 14 15 16 17 18 19 20 21 22 23 24 25 26 27 28 29 30 31
BIO35: Surface Net Thermal Radiation
-log10(P)
10 20 30 40 50 60 70
1 2 3 4 5 6 7 8 9 10 11 12 13 14 15 16 17 18 19 20 21 22 23 24 25 26 27 28 29 30 31

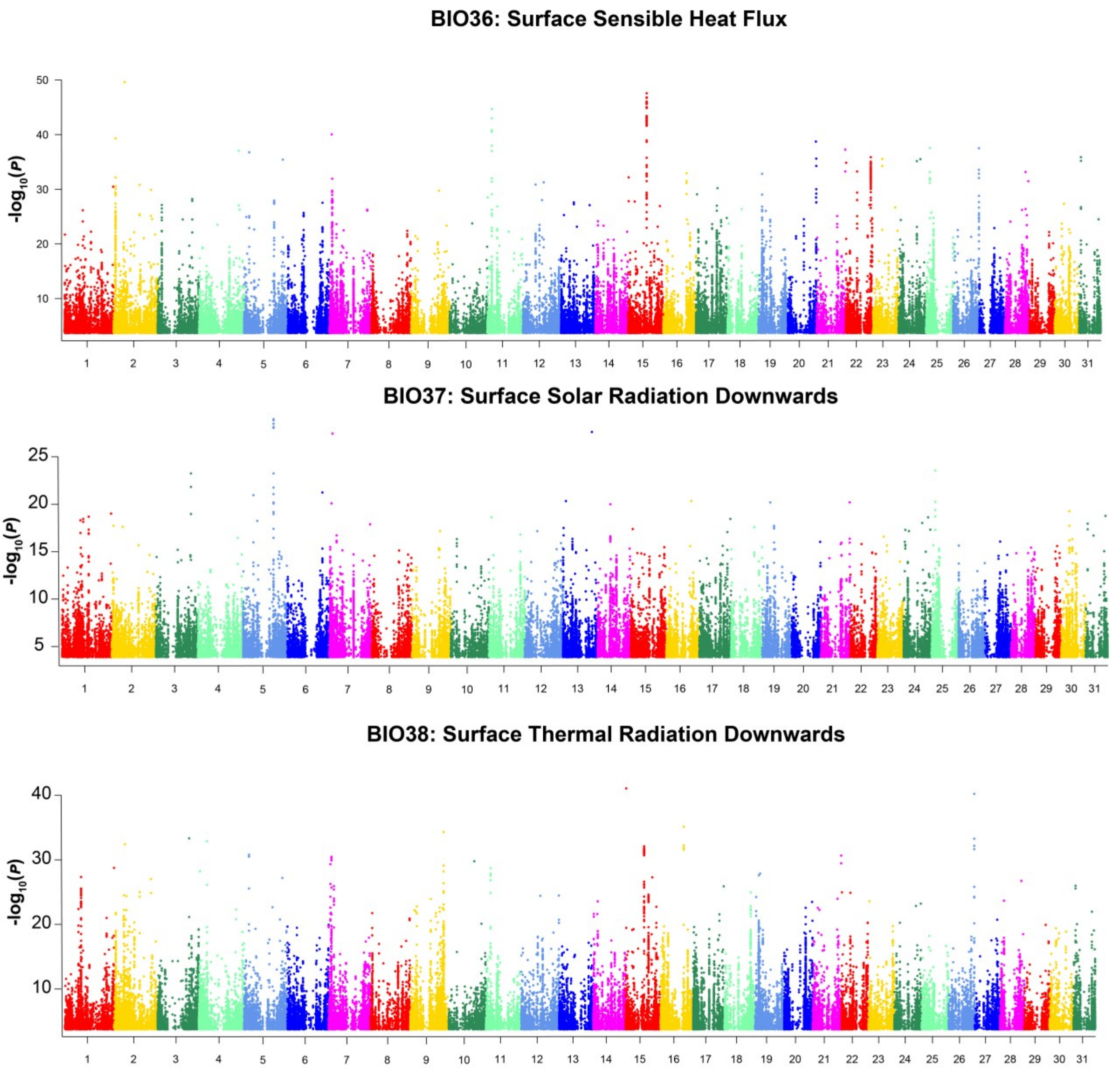

Supplementary Figure 14. Manhattan plot for SNPs associated with the 38 environmental factors. X and y axes are the chromosome number and significance of environmental factor-associated SNPs, respectively. The name of each environmental factor indicated on the top for each subpanel. Different chromosomes are shown in different colors.

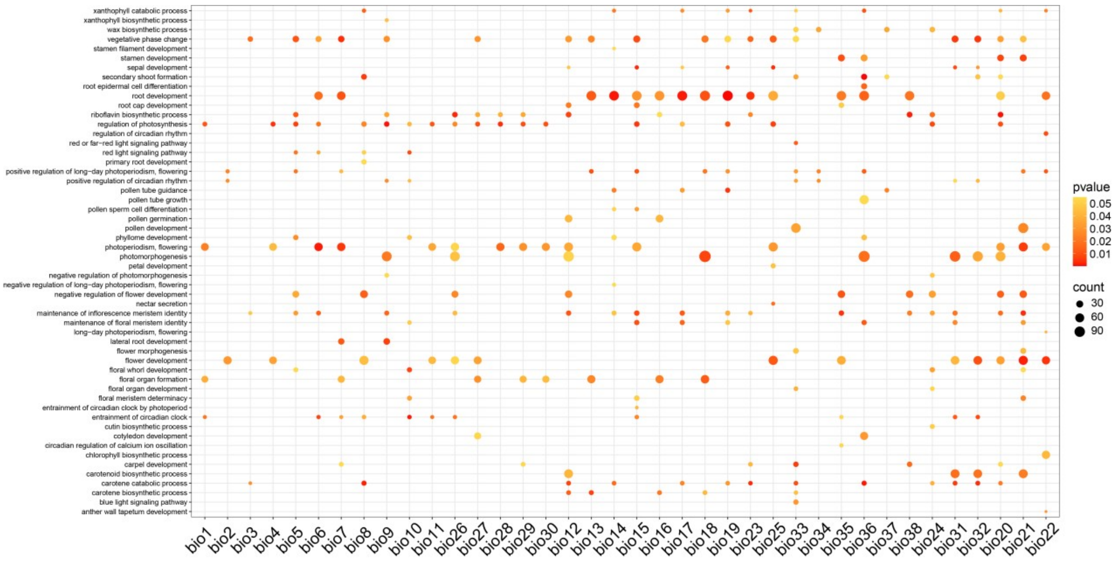

Supplementary Figure 15. Selective GO terms of the candidate genes that showed associations with the 38 environmental factors. Each column represents environmental factor associated GO terms. Circle size indicates the number of genes for each GO terms. Colors from orange to red are significant values. GO term IDs are shown on the y axes. Full GO terms of the 38 environmental factors associated genes were included in Supplementary Table 12.

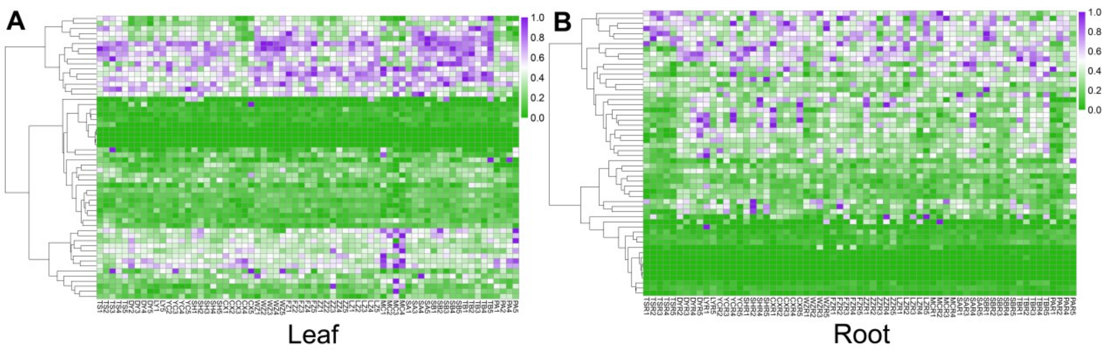

Supplementary Figure 16. Expression pattern of the flowering-related genes in leaf (**A**) and root (**B**) that showed high genetic differentiation between native US, low- and high-latitude Chinese populations.

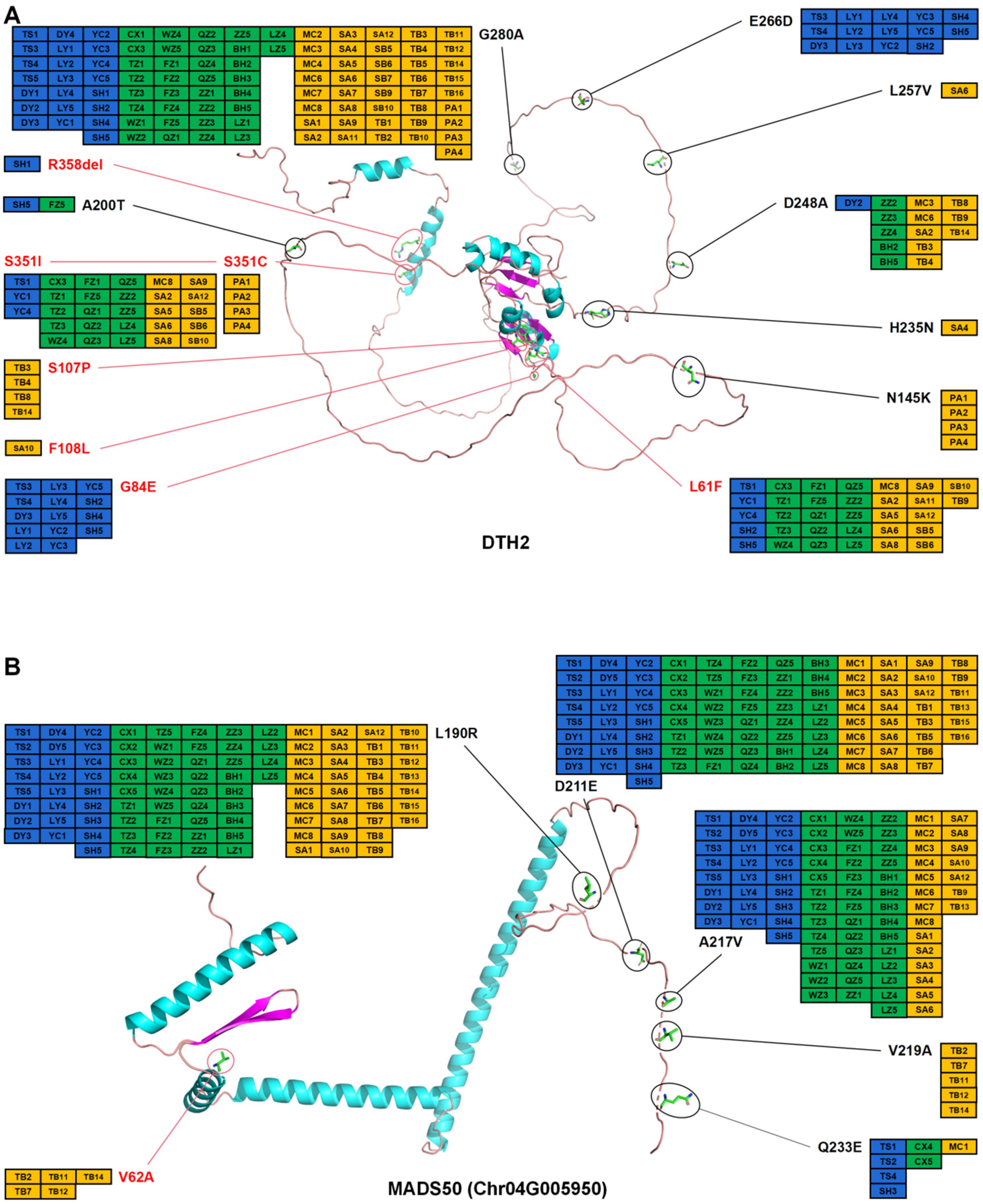
A
TS1 DY4 YC2 CX1 WZ4 QZ2 ZZ5 LZ4 MC2 SA3 SA12 TB3 TB11
TS3 LY1 YC3 CX3 WZ5 QZ3 BH1 LZ5 MC3 SA4 SB5 TB4 TB12
TS4 LY2 YC4 TZ1 FZ1 QZ4 BH2 MC4 SA5 SB6 TB5 TB14
TS5 LY3 YC5 TZ2 FZ2 QZ5 BH3 MC6 SA6 SB7 TB6 TB15
DY1 LY4 SH1 TZ3 FZ3 ZZ1 BH4 MC7 SA7 SB9 TB7 TB16
DY2 LY5 SH2 TZ4 FZ4 ZZ2 BH5 MC8 SA8 SB10 TB8 PA1
DY3 YC1 SH4 WZ1 FZ5 ZZ3 LZ1 SA1 SA9 TB1 TB9 PA2
SH5 WZ2 QZ1 ZZ4 LZ3 SA2 SA11 TB2 TB10 PA3
PA4
G280A
E266D
TS3 LY1 LY4 YC3 SH4
TS4 LY2 LY5 YC5 SH5
DY3 LY3 YC2 SH2
L257V
SA6
SH1
R358del
SH5 FZ5
A200T
D248A
DY2 ZZ2 MC3 TB8
ZZ3 MC6 TB9
ZZ4 SA2 TB14
BH2 TB3
BH5 TB4
S351I
S351C
TS1 CX3 FZ1 QZ5 MC8 SA9 PA1
YC1 TZ1 FZ5 ZZ2 SA2 SA12 PA2
YC4 TZ2 QZ1 ZZ5 SA5 SB5 PA3
TZ3 QZ2 LZ4 SA6 SB6 PA4
WZ4 QZ3 LZ5 SA8 SB10
H235N
SA4
TB3 TB4 TB8 TB14
S107P
N145K
PA1 PA2 PA3 PA4
SA10
F108L
G84E
TS3 LY3 YC5
TS4 LY4 SH2
DY3 LY5 SH4
LY1 YC2 SH5
LY2 YC3
L61F
TS1 CX3 FZ1 QZ5 MC8 SA9 SB10
YC1 TZ1 FZ5 ZZ2 SA2 SA11 TB9
YC4 TZ2 QZ1 ZZ5 SA5 SA12
SH2 TZ3 QZ2 LZ4 SA6 SB5
SH5 WZ4 QZ3 LZ5 SA8 SB6
DTH2
B
TS1 DY4 YC2 CX1 TZ5 FZ4 ZZ3 LZ2 MC1 SA2 SA12 TB10
TS2 DY5 YC3 CX2 WZ1 FZ5 ZZ4 LZ3 MC2 SA3 TB1 TB11
TS3 LY1 YC4 CX3 WZ2 QZ1 ZZ5 LZ4 MC3 SA4 TB3 TB12
TS4 LY2 YC5 CX4 WZ3 QZ2 BH1 LZ5 MC4 SA5 TB4 TB13
TS5 LY3 SH1 CX5 WZ4 QZ3 BH2 MC5 SA6 TB5 TB14
DY1 LY4 SH2 TZ1 WZ5 QZ4 BH3 MC6 SA7 TB6 TB15
DY2 LY5 SH3 TZ2 FZ1 QZ5 BH4 MC7 SA8 TB7 TB16
DY3 YC1 SH4 TZ3 FZ2 ZZ1 BH5 MC8 SA9 TB8
SH5 TZ4 FZ3 ZZ2 LZ1 SA1 SA10 TB9
L190R
TS1 DY4 YC2 CX1 TZ4 FZ2 QZ5 BH3 MC1 SA1 SA9 TB8
TS2 DY5 YC3 CX2 TZ5 FZ3 ZZ1 BH4 MC2 SA2 SA10 TB9
TS3 LY1 YC4 CX3 WZ1 FZ4 ZZ2 BH5 MC3 SA3 SA12 TB11
TS4 LY2 YC5 CX4 WZ2 FZ5 ZZ3 LZ1 MC4 SA4 TB1 TB13
TS5 LY3 SH1 CX5 WZ3 QZ1 ZZ4 LZ2 MC5 SA5 TB3 TB15
DY1 LY4 SH2 TZ1 WZ4 QZ2 ZZ5 LZ3 MC6 SA6 TB5 TB16
DY2 LY5 SH3 TZ2 WZ5 QZ3 BH1 LZ4 MC7 SA7 TB6
DY3 YC1 SH4 TZ3 FZ1 QZ4 BH2 LZ5 MC8 SA8 TB7
SH5
D211E
TS1 DY4 YC2 CX1 WZ4 ZZ2 MC1 SA7
TS2 DY5 YC3 CX2 WZ5 ZZ3 MC2 SA8
TS3 LY1 YC4 CX3 FZ1 ZZ4 MC3 SA9
TS4 LY2 YC5 CX4 FZ2 ZZ5 MC4 SA10
TS5 LY3 SH1 CX5 FZ3 BH1 MC5 SA12
DY1 LY4 SH2 TZ1 FZ4 BH2 MC6 TB9
DY2 LY5 SH3 TZ2 FZ5 BH3 MC7 TB13
DY3 YC1 SH4 TZ3 QZ1 BH4 MC8
SH5 TZ4 QZ2 BH5 SA1
TZ5 QZ3 LZ1 SA2
WZ1 QZ4 LZ2 SA3
WZ2 QZ5 LZ3 SA4
WZ3 ZZ1 LZ4 SA5
LZ5 SA6
A217V
V219A
TB2 TB7 TB11 TB12 TB14
Q233E
TS1 CX4 MC1
TS2 CX5
TS4
SH3
TB2 TB11 TB14
TB7 TB12
V62A
MADS50 (Chr04G005950)

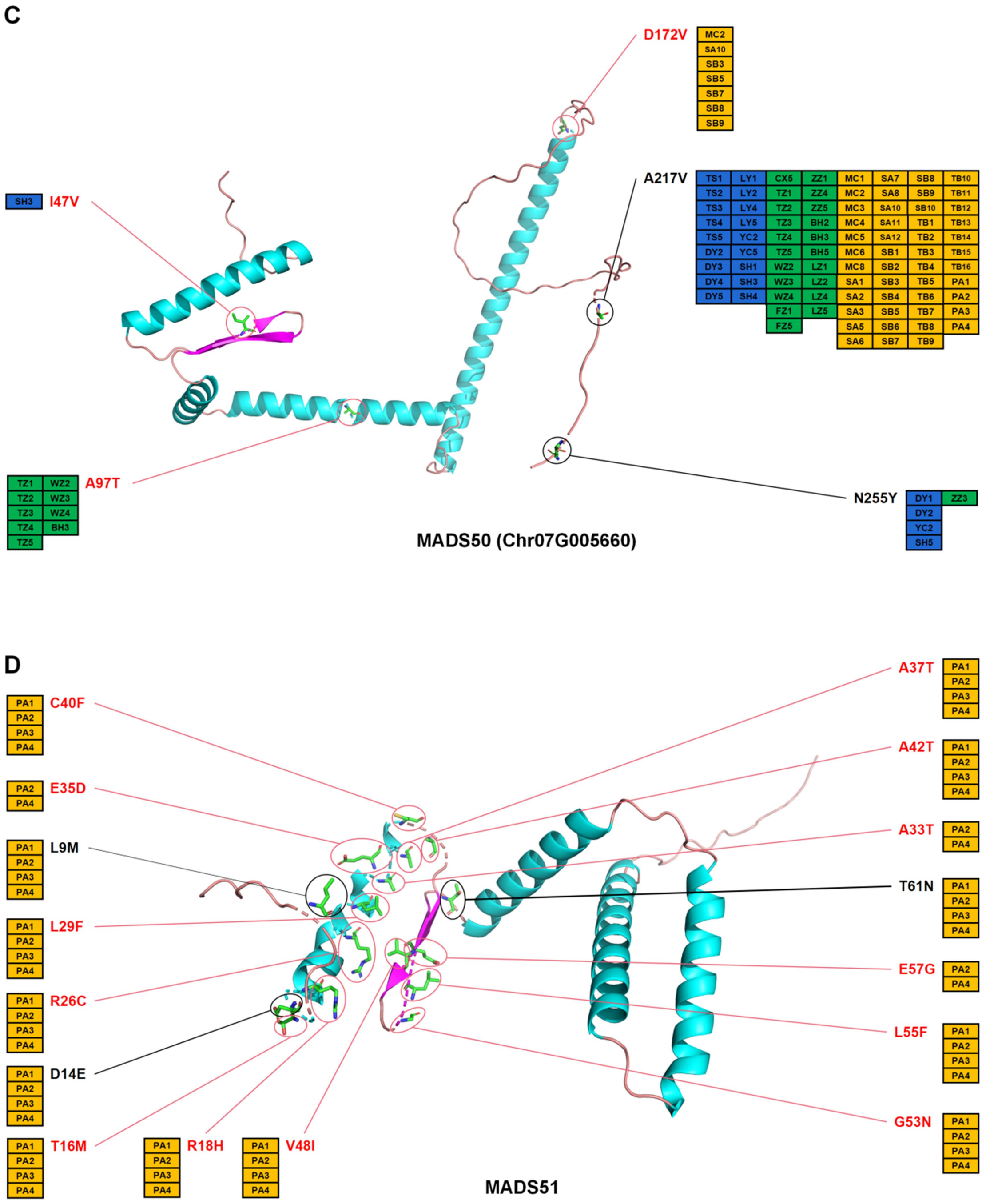
C
D172V
MC2 SA10 SB3 SB5 SB7 SB8 SB9
SH3
I47V
A217V
TS1 LY1 CX5 ZZ1 MC1 SA7 SB8 TB10
TS2 LY2 TZ1 ZZ4 MC2 SA8 SB9 TB11
TS3 LY4 TZ2 ZZ5 MC3 SA10 SB10 TB12
TS4 LY5 TZ3 BH2 MC4 SA11 TB1 TB13
TS5 YC2 TZ4 BH3 MC5 SA12 TB2 TB14
DY2 YC5 TZ5 BH5 MC6 SB1 TB3 TB15
DY3 SH1 WZ2 LZ1 MC8 SB2 TB4 TB16
DY4 SH3 WZ3 LZ2 SA1 SB3 TB5 PA1
DY5 SH4 WZ4 LZ4 SA2 SB4 TB6 PA2
FZ1 LZ5 SA3 SB5 TB7 PA3
FZ5 SA5 SB6 TB8 PA4
SA6 SB7 TB9
TZ1 WZ2
TZ2 WZ3
TZ3 WZ4
TZ4 BH3
TZ5
A97T
N255Y
DY1 ZZ3
DY2
YC2
SH5
MADS50 (Chr07G005660)
D
A37T PA1 PA2 PA3 PA4
C40F PA1 PA2 PA3 PA4
A42T PA1 PA2 PA3 PA4
E35D PA2 PA4
A33T PA2 PA4
L9M PA1 PA2 PA3 PA4
T61N PA1 PA2 PA3 PA4
L29F PA1 PA2 PA3 PA4
E57G PA2 PA4
R26C PA1 PA2 PA3 PA4
L55F PA1 PA2 PA3 PA4
D14E PA1 PA2 PA3 PA4
G53N PA1 PA2 PA3 PA4
T16M PA1 PA2 PA3 PA4
R18H PA1 PA2 PA3 PA4
V48I PA1 PA2 PA3 PA4
MADS51

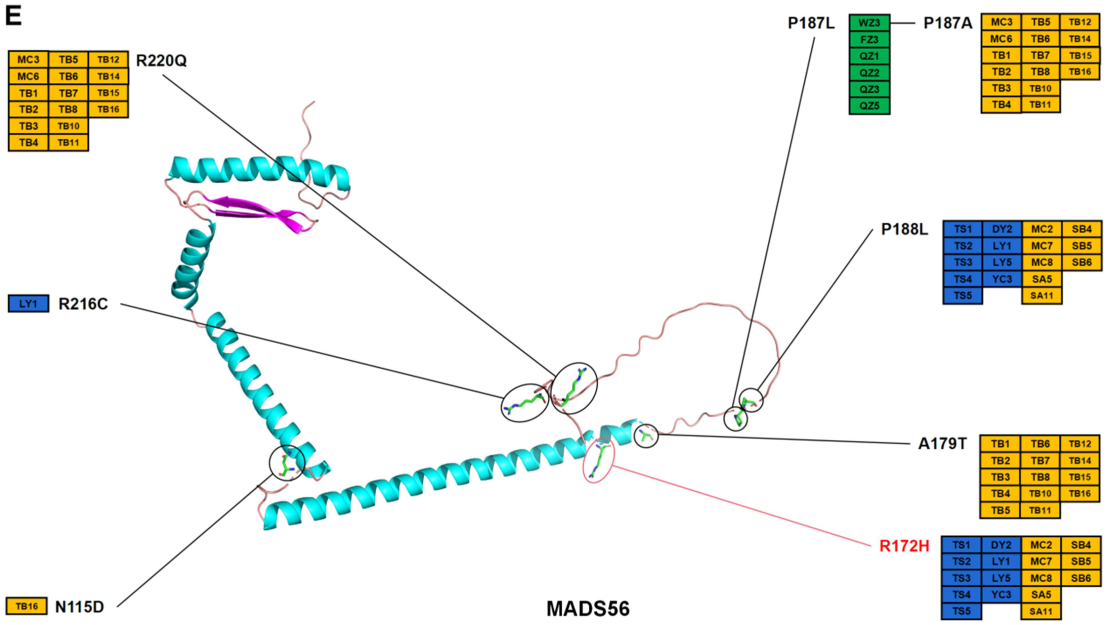

Supplementary Figure 17. Simulation of protein structure of the high divergence flowering genes. Non-synonymous mutations were indicated on left and right sides of each gene. Amino acid highlighted with red color indicated that these non-synonymous mutations lead to structural variation. Accessions numbers in blue, green and orange boxes represent high-, low-latitude Chinese and native US accessions, respectively.

Supplementary Table 1. Basic information of Pacbio and Nanopore long reads used in this study.

Parameter	PacBio reads	Nanopore reads
Reads number	71,810,411	9,935,868
Reads base (bp)	905,675,901,824	251,055,570,106
Reads N50 (bp)	13,929	34,411
Reads max (bp)	675,806	291,196

Supplementary Table 2. Features of the assembled genome of *S. alterniflora*.

Assembly	Parameter
Total length of scaffold (bp)	1,631,144,040
Scaffold number	763
Contig number	1,002
Scaffold N50 (bp)	50,761,989
Contig N50 (bp)	18,800,000
Scaffold max (bp)	73,030,954
Contig max (bp)	63,413,729
GC content (%)	44.95
Illumina reads mapping rate (%)	99.20
PacBio reads mapping rate (%)	99.95
CEGMA (%)	96.72
Complete and single-copy BUSCOs (%)	43.26
Complete and duplicated BUSCOs (%)	52.57
Fragmented BUSCOs (%)	0.42
Missing BUSCOs (%)	3.75
Hi-C reads (Gb)	251.49
Hi-C reads mapping rate (%)	97.73

Supplementary Table 3. Information of the protein-coding genes in *S. alterniflora* genome.

Annotation	Number
Number of protein-coding genes	73,711
Total length of protein-coding genes (bp)	282,156,950
Mean length of genes (bp)	3,827.88
Mean length of exons (bp)	1,432.95
Mean length of CDS (bp)	1,161.98
Mean length of intros (bp)	2,394.93
Number of pseudogenes	6,248
Number of miRNAs	233
Number of rRNAs	5,727
Number of tRNAs	978
GO_Annotation (%)	71.41
KEGG_Annotation (%)	33.53
KOG_Annotation (%)	50.19
TrEMBL_Annotation (%)	94.14
nr_Annotation (%)	94.04
All_Annotated (%)	94.32
