# Supplementary material for: Genomic and phenotypic signatures provide insights into the wide adaptation of a global plant invader
Source: Plant Commun. 2024 Jan 13;5(4):100820. doi: 10.1016/j.xplc.2024.100820 (PMC11009367; doi:10.1016/j.xplc.2024.100820)
Supplement: Document S2. Article plus supplemental information [file mmc8.pdf]

Plant Communications
Research article

CellPress Partner Journal

Genomic and phenotypic signatures provide insights into the wide adaptation of a global plant invader

Yan Hao[1], Xin-Feng Wang[2], Yaolin Guo[1], Tian-Yang Li[1], Ji Yang[1], Malika L. Ainouche[3], Armel Salmon[3], Rui-Ting Ju[1], Ji-Hua Wu[4,*], Lin-Feng Li[1,2,*] and Bo Li[1,5,*]

[1]National Observations and Research Station for Wetland Ecosystems of the Yangtze Estuary and Ministry of Education Key Laboratory for Biodiversity Science and Ecological Engineering, Institute of Biodiversity Science and Institute of Eco-Chongming, School of Life Sciences, Fudan University, Shanghai 200438, China

[2]State Key Laboratory of Biocontrol, Guangdong Provincial Key Laboratory of Plant Resources, School of Life Sciences, Sun Yat-sen University, Guangzhou 510275, China

[3]UMR CNRS 6553, Université of Rennes, Campus de Beaulieu, 35042 Rennes Cedex Paris, France

[4]State Key Laboratory of Herbage Improvement and Grassland Agro-ecosystems, College of Ecology, Lanzhou University, Lanzhou 730000, China

[5]Ministry of Education Key Laboratory for Transboundary Ecosecurity of Southwest China, Yunnan Key Laboratory of Plant Reproductive Adaptation and Evolutionary Ecology and Centre for Invasion Biology, Institute of Biodiversity, School of Ecology and Environmental Science, Yunnan University, Kunming, Yunnan 650504, China

***Correspondence: Bo Li (Bool@ynu.edu.cn), Ji-Hua Wu (wjh@lzu.edu.cn), Lin-Feng Li (lilinfeng@fudan.edu.cn)**

https://doi.org/10.1016/j.xplc.2024.100820

ABSTRACT

Invasive alien species are primary drivers of biodiversity loss and species extinction. Smooth cordgrass (*Spartina alterniflora*) is one of the most aggressive invasive plants in coastal ecosystems around the world. However, the genomic bases and evolutionary mechanisms underlying its invasion success have remained largely unknown. Here, we assembled a chromosome-level reference genome and performed phenotypic and population genomic analyses between native US and introduced Chinese populations. Our phenotypic comparisons showed that introduced Chinese populations have evolved competitive traits, such as early flowering time and greater plant biomass, during secondary introductions along China's coast. Population genomic and transcriptomic inferences revealed distinct evolutionary trajectories of low- and high-latitude Chinese populations. In particular, genetic mixture among different source populations, together with independent natural selection acting on distinct target genes, may have resulted in high genome dynamics of the introduced Chinese populations. Our study provides novel phenotypic and genomic evidence showing how smooth cordgrass rapidly adapts to variable environmental conditions in its introduced ranges. Moreover, candidate genes related to flowering time, fast growth, and stress tolerance (*i.e.*, salinity and submergence) provide valuable genetic resources for future improvement of cereal crops.

Key words: adaptation, natural selection, invasive species, population genomics, *Spartina alterniflora*

Hao Y., Wang X.-F., Guo Y., Li T.-Y., Yang J., Ainouche M.L., Salmon A., Ju R.-T., Wu J.-H., Li L.-F., and Li B. (2024). Genomic and phenotypic signatures provide insights into the wide adaptation of a global plant invader. Plant Comm. **5**, 100820.

INTRODUCTION

Invasive alien species are a leading threat to global biodiversity, ecosystem integrity, and ecological functioning (Ehrenfeld, 2003; Powell et al., 2011; Doherty et al., 2016). The wide-scale introduction and spread of invasive alien species alter terrestrial and aquatic ecosystems and their functions around the world, providing excellent models with which to address how alien species rapidly adapt to novel ranges (Sakai et al., 2001; Gurevitch and Padilla, 2004; Sax et al., 2007). However, although extensive attempts have been made to understand the mechanisms underpinning invasion success, they have produced mixed conclusions (MacDougall and Turkington, 2005). In particular, why some alien species are prone to become invasive, whereas others fail to even survive as sustainable populations, remains largely unknown (Barrett, 2015).

Published by the Plant Communications Shanghai Editorial Office in association with Cell Press, an imprint of Elsevier Inc., on behalf of CSPB and CEMPS, CAS.

© 2024 The Author(s).
This is an open access article under the CC BY license (http://creativecommons.org/licenses/by/4.0/).

Numerous hypotheses have been proposed to explain the mechanisms underlying the invasion success of alien species (Jeschke et al., 2014). The enemy release hypothesis posits that escape from specialized natural enemies in non-native ranges enables alien species to shift resources from defense (*i.e.*, anti-herbivore defense) toward competitive ability (*i.e.*, high resource acquisition, fast growth, and high reproductive rate) (Muller-Scharer et al., 2004; Doorduin and Vrieling, 2011; Kowarik et al., 2012). Indeed, empirical evidence has confirmed that invasive alien plants suffer less damage from herbivores and pathogens than native species (Muller-Scharer et al., 2004; Ebeling et al., 2008; Bieker et al., 2022). In line with this, the evolution of increased competitive ability (EICA) hypothesis proposes that invasive alien species often show increased competitiveness in their introduced ranges, including rapid seedling growth, larger inflorescences, and more/heavier seeds (Blossey and Notzold, 1995; Ebeling et al., 2008; van Kleunen et al., 2015; Callaway et al., 2022). In addition, the propagule pressure hypothesis argues that the invasion success of an alien species is determined mainly by the average number of release events per unit time and the number of individuals per release event (Lockwood et al., 2005; Simberloff, 2009; Blackburn et al., 2013; Jeschke et al., 2014). Under this hypothesis, alien species that have experienced multiple introductions from diverse source populations are expected to show high genetic diversity and phenotypic plasticity (Allendorf and Lundquist, 2003; Ghalambor et al., 2007; Bock et al., 2016).

Coastal salt marshes are the most productive and economically important ecosystems (Costanza, 1999; Barbier et al., 2011; He and Silliman, 2019). However, the biodiversity and functioning of coastal ecosystems are facing unprecedented changes due to alien species invasion, global climate change, and other drivers (Jackson et al., 2001; Hensel et al., 2021; Herbert-Read et al., 2022). In the salt marshes of North America, for example, frequent large-scale disturbances caused by climate change and anthropogenic activities have led to loss of the foundation species *Spartina alterniflora* (2n = 62) (hereafter called smooth cordgrass) (Stiven and Gardner, 1992; McKee et al., 2004; Alber et al., 2008; Angelini and Silliman, 2012; Hensel et al., 2021). In other parts of the world, however, smooth cordgrass has become one of the most aggressive invaders of coastal ecosystems (Wang et al., 2006; Strong and Ayres, 2013). In Europe, smooth cordgrass was introduced during the 19th century and underwent independent hybridizations with the congeneric native *Spartina maritima* (2n = 60) in England and France (Aïnouche et al., 2009). Genome doubling of the hybrid led to the formation of *Spartina anglica* (2n = 124), which is considered a threat to salt marshes worldwide (van der Weijden et al., 2007). In China, smooth cordgrass was deliberately introduced in 1979 from three source locations in the US (Morehead City, NC; Sapelo Island, GA; and Tampa Bay, FL) for ecological engineering applications, such as seashore stabilization and saline soil mitigation (Xu and Zhuo, 1985; Zhang et al., 2004; An et al., 2007). All seedlings and seeds of smooth cordgrass were initially grown in Luoyuan Bay of Fujian Province (26° 36′ N, 119° 36′ E) (Chung et al., 2004). However, its fast growth ability, together with secondary intentional introductions have resulted in a continuous distribution pattern of smooth cordgrass along the coast of China (Qin et al., 1985; Wang et al., 2006). High phenotypic plasticity and genetic diversity are proposed as key contributors to the invasion success of smooth cordgrass in China (Wang et al., 2012; Chen et al., 2015, 2021; Liu et al., 2016, 2020; Shang et al., 2019). Genetic mixture of different US source populations has also been proposed as a potential mechanism of invasion success (Qiao et al., 2019; Shang et al., 2019). However, the genomic basis and evolutionary mechanism(s) that underpin its invasion success have remained largely unclear.

In this study, we compared vegetative and reproductive traits between native US and introduced Chinese cordgrass populations. Our comparisons revealed that introduced Chinese populations have not only evolved earlier-flowering phenotypes in high-latitude regions but also exhibit greater plant height and biomass than native US populations. To further explore the genomic basis underlying the phenotypic determinants of invasion success, we assembled a high-quality reference genome of a US smooth cordgrass accession (S13) at the chromosome level. We then performed population genomic and transcriptomic comparisons between the native US and introduced Chinese populations. Our results show that genes related to flowering time and plant growth are associated with invasion success along China's coast. A combination of phenotypic and genomic data demonstrates that rapid evolution of competitive traits has conferred high adaptability, enabling smooth cordgrass to cope with varying environmental conditions in its novel ranges. Our study provides new insights into how an alien species rapidly adapts to non-native habitats. In addition, cordgrass species exhibit highly efficient sodium secretion in which excess absorbed sodium is removed through salt glands (Figure 1A). This attribute confers cordgrass species with high adaptability to waterlogging and salinity stress in coastal ecosystems. Candidate genes associated with salinity and submergence tolerance provide valuable genetic resources for improvement of cereal crops in response to flooding and salt stress.

RESULTS AND DISCUSSION

Genome assembly, annotation, and gene family evolution

Our flow cytometry and fluorescence *in situ* hybridization analysis confirmed the genome size (1.48–1.52 Gb) (Supplemental Figure 1A) and karyotype of smooth cordgrass (2n = 62) (Supplemental Figure 1B), as reported previously (Ainouche et al., 2004; Ayres et al., 2008). To obtain a high-quality genome assembly of the selected US sample (S13), 251.06 Gb (~154.02×) Nanopore and 905.68 Gb (~555.25×) PacBio long reads were produced using the Oxford Nanopore (ONT, UK) and PacBio Sequel (PacBio, USA) platforms (Supplemental Table 1). A genome survey revealed high complexity of genome features (genome size = 1.71 Gb, heterozygosity = 0.21%), with 1.03 Gb (60.23%) of the total genome consisting of repetitive sequences (Supplemental Figure 1C). The draft genome assembly was 1.63 Gb in length (Table 1) with a contig N50 of 28.25 Mb, which was close to the genome size estimated by flow cytometry and the genome survey. Chromosome-scale scaffolding was performed using 251.49 Gb (154.29× genome coverage) paired-end high-throughput chromosome conformation capture (Hi-C) short reads, with 1.59 Gb (97.73% of the total) contigs successfully assigned to 31 pseudomolecules (Supplemental Table 2). The contig

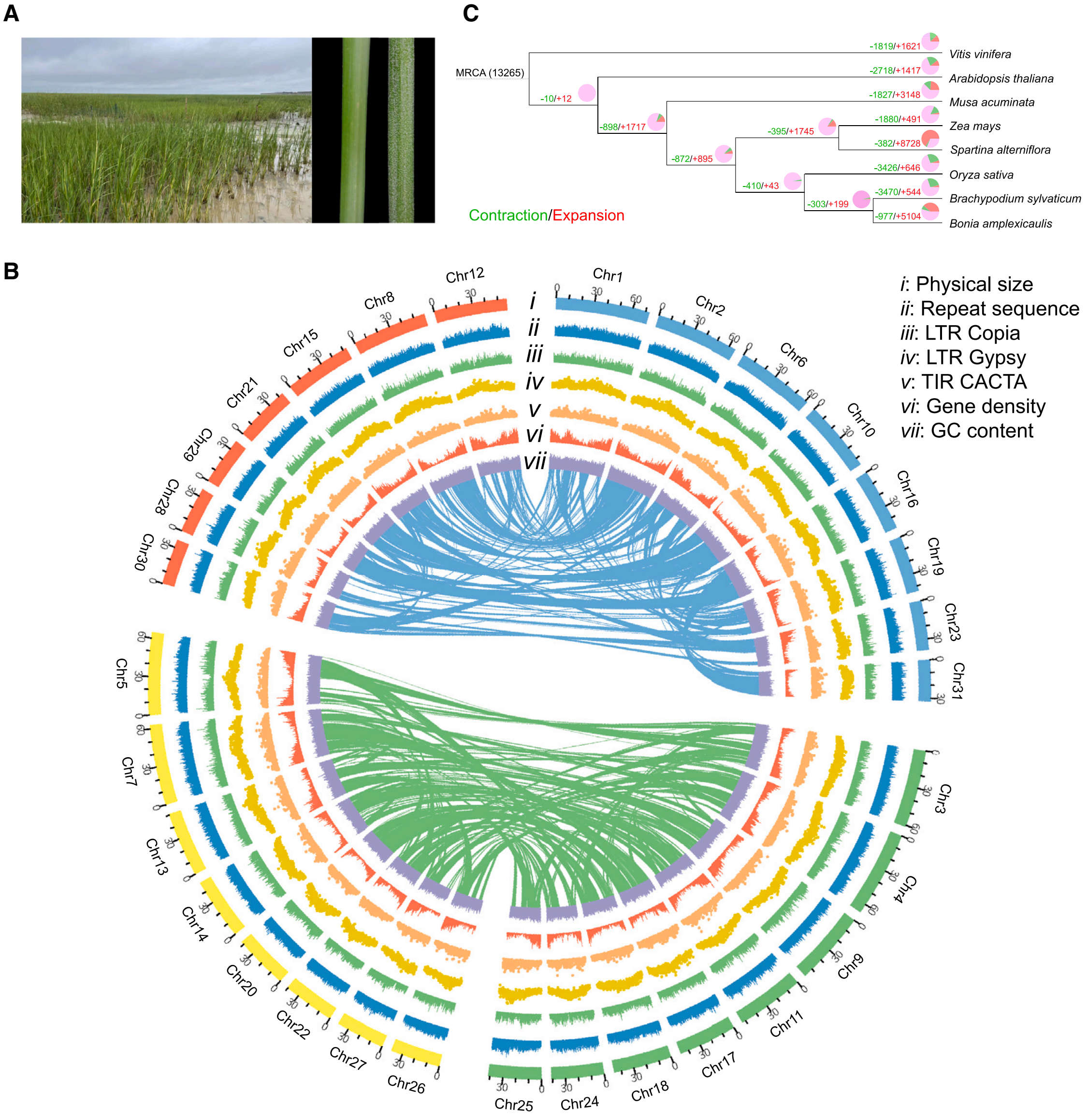

Figure 1. Natural habitat in salt marshes, genome features, and gene family evolution of smooth cordgrass.
(A) Left: the natural habitat of smooth cordgrass in Beihai (southern China). Right: sodium secretion of smooth cordgrass at 10% salt concentration (right leaf) compared with fresh water (left leaf). The small white dots on the right leaf are secreted salt.
(B) The tracks of the circular diagram from outside to inside represent (i) the physical size of chromosomes, with tick marks placed at 30-Mb intervals; (ii) the distribution of total repetitive sequences; (iii) *Copia*-like retrotransposon density; (iv) *Gypsy*-like retrotransposon density; (v) CACTA DNA transposon density; (vi) density of high-confidence genes; and (vii) GC content (percent).
(C) Gene family expansions (red) and contractions (green) in smooth cordgrass and seven other plant species. Numbers of expanded and contracted gene families are shown on the branches. Red, green, and pink colors in the pie charts indicate proportions of expanded, contracted, and balanced gene families, respectively. MRCA, most recent common ancestor of the eight species.

N50 of the final genome assembly was 18.80 Mb (Supplemental Table 2).

A total of 73 711 protein-coding genes corresponding to 282.16 Mb (17.30%) of the genome were annotated, 69 524 (94.32%) of which had homology with known genes in public databases (Supplemental Table 3). We also annotated 6938 noncoding RNAs, including 233 microRNAs, 5727 rRNAs, and 978 tRNAs (Supplemental Table 3). The majority of protein-coding genes were distributed at telomeric regions across all 31 chromosomes (Figure 1B). By contrast, both *Copia* and *Gypsy* transposons clustered at centromeric regions. However, GC content maintained a similar distribution pattern (44.95%) along all the chromosomes. Quality control of the assembled genome revealed that 99.20% of the Illumina short reads and 99.95% of the PacBio long reads were successfully mapped onto the reference genome (Supplemental Table 2). Genome completeness based on annotated protein-coding genes also confirmed the high quality of the assembled genome (CEGMA: Core Eukaryotic Genes Mapping Approach = 96.72% and BUSCO: Benchmarking Universal Single-Copy Orthologs = 95.83%) (Table 1 and Supplemental Table 2).

Assembly feature	Value
Estimated genome size (Gb)	1.71
Flow cytometry genome size (Gb)	1.48–1.52
Assembled genome size (Gb)	1.63
Number of contigs	1002
Total length of contigs (bp)	1 631 120 140
N50 of contigs (bp)	18 800 000
Masked repetitive sequence length (bp)	1 192 775 858
Number of genes	73 711
Complete BUSCOs (%)	95.83
CEGMA (%)	96.72

Table 1. Features of the *S. alterniflora* genome assembly.

We performed comparative analysis of the protein-coding genes in smooth cordgrass, four grass family species (rice, maize, false brome, and bamboo), and three other species (banana, *Arabidopsis*, and grape). A total of 28 028 gene families were identified in the 8 species, 1886 of which were specific to smooth cordgrass. Among the identified gene families, 382 and 8728 had experienced significant (adjusted $p > 0.05$) contraction or expansion in smooth cordgrass, respectively (Figure 1C). Gene Ontology (GO) enrichment analysis of the cordgrass-specific gene families identified some adaptation-related pathways, such as salt tolerance (trehalose biosynthetic process), sexual reproduction, maintenance of shoot apical meristem identity, and telomere maintenance (Supplemental Table 4). For example, a gene cluster containing 7 copies of the trehalose 6-phosphate phosphatase (TPS) gene was identified in the cordgrass-specific gene families. Given that trehalose acts as a stress-protective agent to reduce damage to plant tissues, multiple copies of the *TPS* gene are potentially associated with salinity tolerance in salt marsh habitats. Likewise, the expanded gene families were functionally enriched in submergence and salinity tolerance (*i.e.*, response to reactive oxygen species, cell redox homeostasis, aerenchyma formation, and calcium-mediated signaling), phytohormone biosynthesis and regulation (*i.e.*, gibberellin biosynthetic process and jasmonic acid- and salicylic acid-mediated signaling pathway), photomorphogenesis (*i.e.*, photoperiodism and far-red light phototransduction), photosynthesis (*i.e.*, chlorophyll binding and photosystem II assembly), and plant development (*i.e.*, seed dormancy process and root and flower development) (Supplemental Table 5). Contracted gene families tended to be enriched in lateral root development, regulation of unidimensional cell growth, and de-etiolation (Supplemental Table 6). These findings together suggest that expanded and specific gene families in smooth cordgrass may contribute to its adaptability to salinity and submergence tolerance in salt marsh habitats.

Morphological differences between native and introduced populations

We performed common garden experiments to compare 12 vegetative and reproductive traits between native US and introduced Chinese populations (Figure 2D). All introduced Chinese populations originated from the same four US source populations (MC: Morehead City, SA: Sapelo Island, SB: Sapelo Island, and TB: Tampa Bay) in three locations (Morehead City, NC; Sapelo Island, GA; and Tampa Bay, FL). If all introduced Chinese populations had evolved neutrally during expansion along the coast of China, then we would expect to observe a similar pattern of variation for these morphological traits in introduced Chinese and US source populations. However, our comparisons revealed that introduced Chinese populations differed significantly from US source populations in the majority of these morphological traits (Figure 2D and Supplemental Figure 2). For example, Chinese populations had a longer flowering duration than US source populations, showing differences in the number of days to first flowering, peak flowering day, and length of the flowering season. Likewise, most Chinese populations also had greater numbers of ramets, percentages of effective ramets (the ratio of flowering tillers at the end of the growing season), and plant height and biomass (*i.e.*, above- and belowground biomass).

During invasion processes along the coast of China, low- and high-latitude Chinese populations evolved phenotypic differences in these vegetative and reproductive traits. For example, compared with high-latitude populations, low-latitude Chinese populations showed higher fitness of vegetative traits, including greater plant height and above- and belowground biomass (Figure 2D and Supplemental Figure 2). By contrast, high-latitude Chinese populations exhibited earlier flowering time than low-latitude populations. These observations suggest that introduced Chinese populations evolved advantageous phenotypes during expansion processes.

Phylogeny, genetic population structure, and demographic history

Population genomic structures of all US and Chinese accessions were inferred from genome-wide SNP datasets. If only two ancestral genetic clusters were assumed, then the Chinese populations exhibited genomic constitutions similar to three of the four US source populations (MC, SA, and SB) (Figure 3A, $K = 2$; Supplemental Table 7). When more ancestral genetic clusters were considered, low-latitude Chinese populations exhibited genetic constitutions distinct from those of both high-latitude Chinese and US source populations (Figure 3A, $K = 2–5$). For example, the low-frequency genetic cluster (*i.e.*, green in $K = 4$) in US source populations became a major genetic cluster in

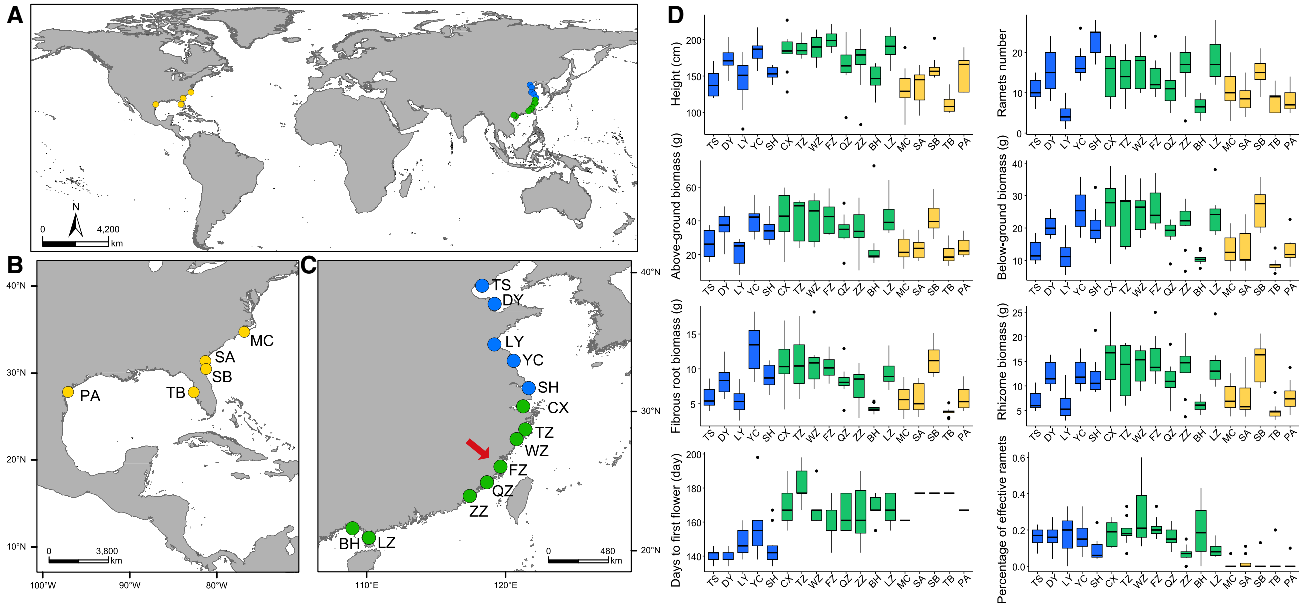

Figure 2. Sampling information and phenotypes of native US and introduced Chinese populations.

(A–C) Geographic locations of the 18 populations collected from the US and China. Yellow, blue, and green dots represent native US and high- and low-latitude Chinese populations, respectively. Population names are shown beside the colored dots. The red arrow indicates the location of the FZ population, where US-sourced cordgrass was grown.

(D) Vegetative and reproductive traits of native US and introduced Chinese populations. Yellow, blue, and green represent US and high- and low-latitude Chinese populations, respectively.

Detailed sampling information and phenotypic data can be found in Supplemental Tables 16 and 17.

low-latitude Chinese populations. By contrast, the high-latitude Chinese populations tended to contain multiple genetic clusters from the US source populations. In particular, a genetic mixture pattern that recombined distinct genetic clusters from the three US source populations was observed in some introduced Chinese accessions (Figure 3A).

A similar phenomenon was observed in the phylogenetic inference, in which the cordgrass accessions were separated into three clades corresponding to native US and low- and high-latitude Chinese populations (Figure 3B). The majority of cordgrass accessions collected from the US source population TB and the US non-source population PA (Port Aransas) showed distinct genetic clusters (K = 3–5) and phylogenetic relationships to the other three US source populations (MC, SA, and SB) and the introduced Chinese populations (Figure 3A and 3B). In line with these observations, the overall genetic differentiation (F_{ST}) between native and introduced low-latitude populations (F_{ST} = 0.103) was also greater than that between native and introduced high-latitude populations (F_{ST} = 0.069) (Supplemental Figure 3A). At the population level, the TB source and PA non-source US populations exhibited higher genetic differentiation (F_{ST} = 0.338–0.556) to all Chinese populations compared to the three US source populations (MC, SA, and SB) (F_{ST} = 0.050-0.251) (Figure 3C; Supplemental Table 8).

We also performed analysis of isolation by distance for native US and introduced Chinese populations. Although a significant association between genetic differentiation and geographic distance (Mantel test, p = 0.0056) was observed among all introduced Chinese populations, we did not identify significance for native US populations (Mantel test, p = 0.0833) or the high-latitude (Mantel test, p = 0.2333) and low-latitude (Mantel test, p = 0.1115) Chinese populations. In addition, our estimates of population split and mixture events based on allele frequency identified long-distance flow events, such as from high-latitude populations TS (TangShan)/LY (LianYunGang) to low-latitude populations ZZ (ZhangZhou)/BH (BeiHai) (Supplemental Figure 3B). These genomic features indicate that the geographic structure of the high- and low-latitude Chinese populations likely formed through secondary intentional introductions from the same source populations.

Linkage disequilibrium (LD) decay was used to estimate the recombination rate for all US and Chinese populations. Our results revealed a lower degree of LD decay in both the low- and high-latitude Chinese populations compared with US populations (Supplemental Figure 4), indicating that introduced Chinese populations may have undergone a genetic bottleneck or natural selection during the process of local adaptation. However, genome-wide estimates of nucleotide diversity (π) revealed that almost all of the Chinese populations (except BH) maintained levels of π similar to those of source and non-source US populations (Figure 3D; Supplemental Table 9). It is therefore highly likely that natural selection, rather than genetic bottlenecks, might have been responsible for the reduced recombination rates in introduced Chinese populations. It is notable that high-latitude Chinese populations (10 699 851, 14.87% of the total SNPs) harbored more specific SNPs than low-latitude Chinese (2 156 611, 3.00%) and native US (1 025 890, 1.43%) populations (Supplemental Figure 5A).

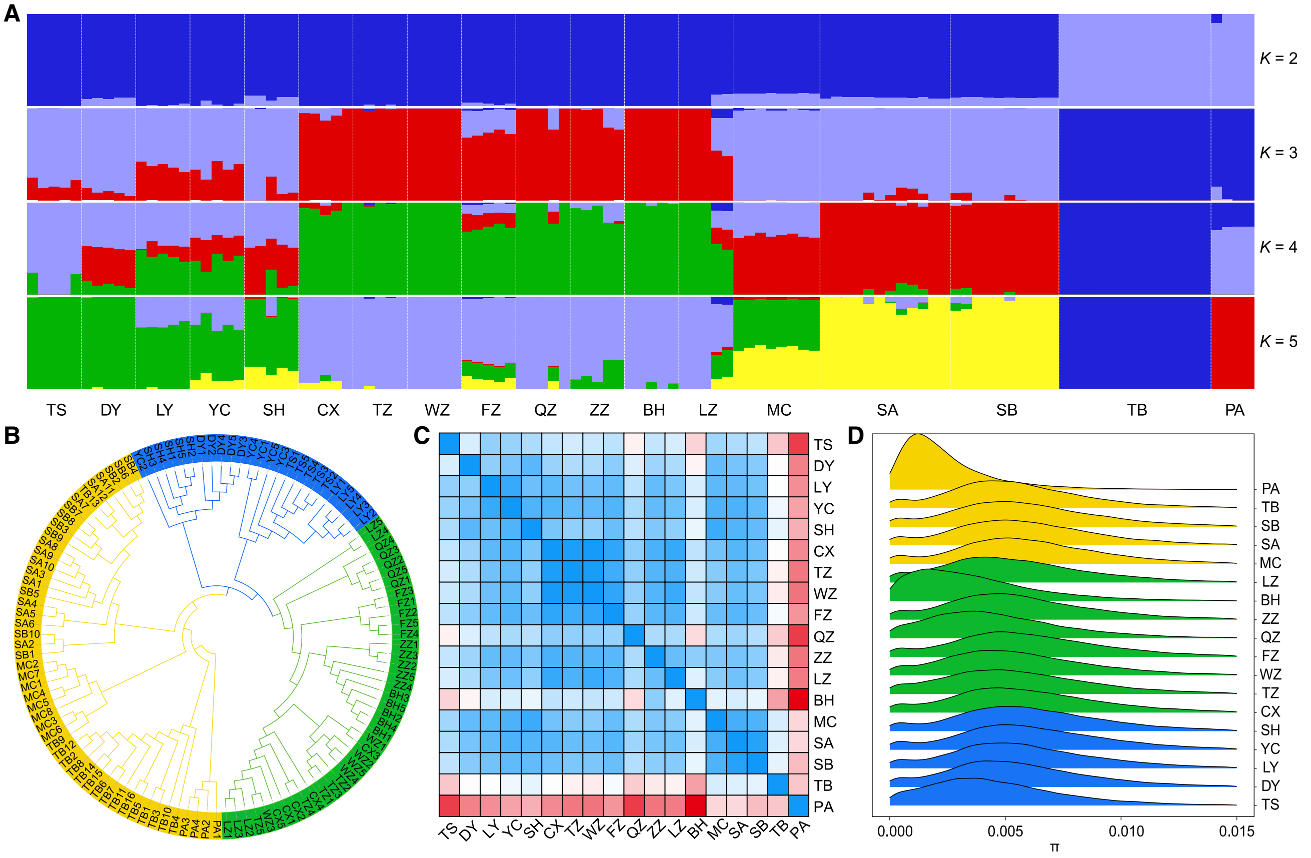

Figure 3. Population structure, phylogeny, and nucleotide diversity of native US and introduced Chinese populations.

(A) Proportions of genetic clusters for each accession from K = 2–5. Different colors represent distinct ancestral genetic clusters. K = 4 is the best genetic cluster.

(B) Neighbor-joining tree of native US and introduced Chinese accessions based on the genome-wide SNP dataset. Yellow, blue, and green represent US and high- and low-latitude Chinese populations, respectively. The accession IDs are the same as those in Supplemental Table 16.

(C) Pairwise genetic differentiation (F_{ST}) among the 18 populations. Colors from blue and white to red indicate low, medium, and high genetic differentiation.

(D) Density of nucleotide diversity (π) (in 50-kb sliding windows) of 18 US and Chinese populations based on the genome-wide SNP dataset. Yellow, blue, and green represent US and high- and low-latitude Chinese populations, respectively.

Likewise, the proportion of genes with specific SNPs was higher in high-latitude Chinese populations (15 436, 24.71% of the total genes) than in low-latitude Chinese (2429, 3.89%) and native US (760, 1.22%) populations (Supplemental Figure 5B). However, both specific and shared SNPs were scattered randomly along the chromosomes in native US and low- and high-latitude Chinese populations (Supplemental Figure 6), suggesting the possibility that the two introduced groups might have inherited distinct genetic variants from US source populations. We also calculated the inbreeding coefficient (Fis) for all Chinese and US accessions (Supplemental Figure 7). The majority of native US accessions had higher Fis values than the invasive Chinese accessions.

Gene expression patterns of the US and Chinese populations

Gene expression patterns of leaf and root tissues were evaluated for common garden samples of the native US and introduced Chinese populations. In contrast to the genetic inferences above, principal-component analysis based on all expressed genes did not identify expression-level divergence between native US and introduced Chinese populations in leaf or root tissues (Supplemental Figure 8). Instead, US and Chinese accessions showed a mixed pattern in both leaf and root tissues. Our analysis also identified differentially expressed genes (DEGs) among native US and low- and high-latitude Chinese populations. However, differential expression of these DEGs was mainly identified in a part of accessions from the three groups (native US and low- and high-latitude Chinese populations). In other words, these DEGs did not show consistent higher or lower expression in all accessions of one group compared with the other two groups (Supplemental Figure 9). A similar pattern was also observed for genes with tissue-specific expression; a majority of these genes were not differentially expressed between Chinese and US populations in the two tissues (Supplemental Figure 10). On the other hand, in root tissue, introduced Chinese groups (except the low-latitude populations) exhibited higher individual-level expression divergence than the native US groups (all US, source US, and non-source US) (Supplemental Figure 11). By contrast, an opposite expression pattern was observed for leaf tissue between Chinese and US populations, with all three

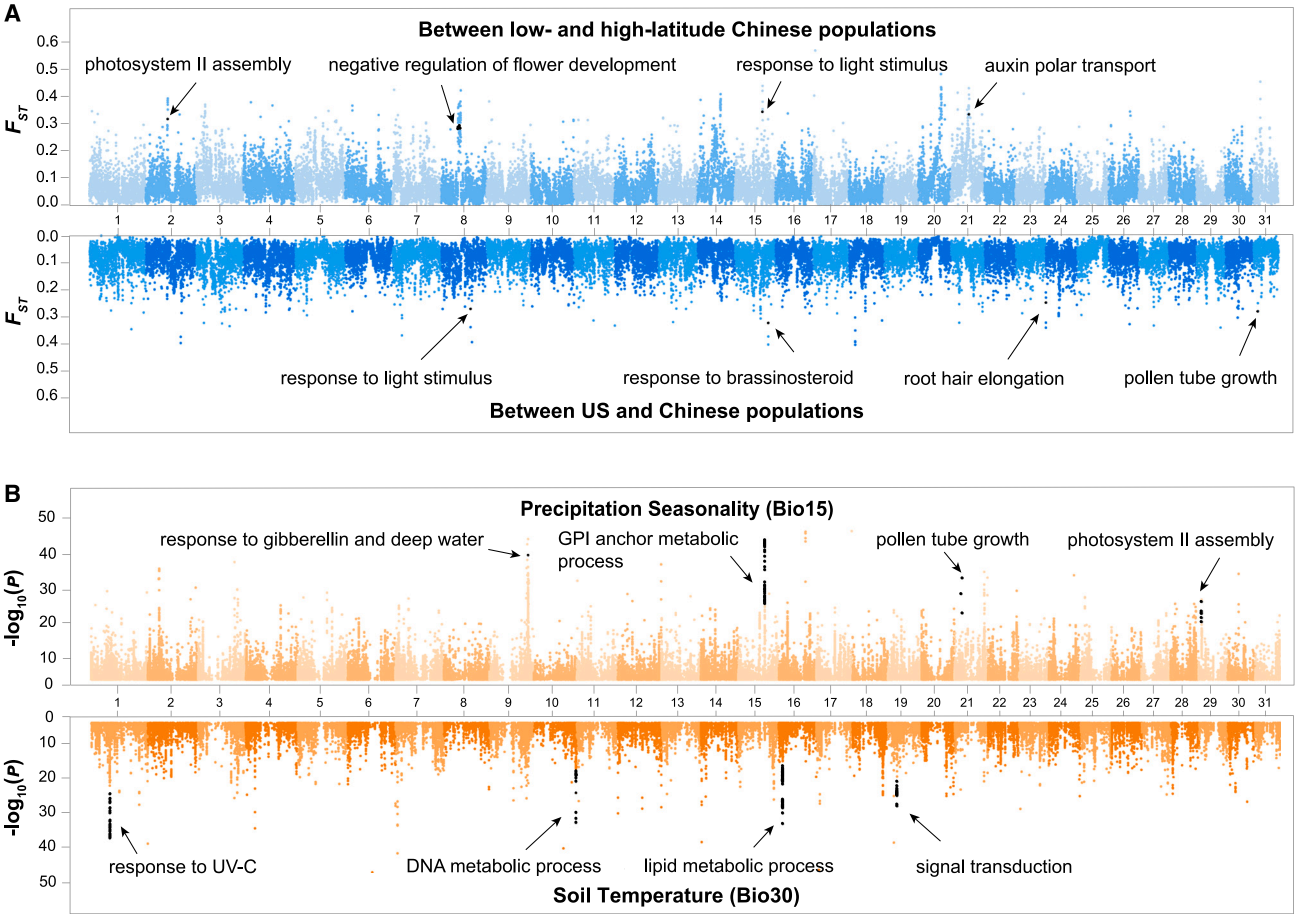

Figure 4. Genome-wide scan for high-differentiation genomic regions and environmental factor-associated variants in native US and introduced Chinese populations.

(A) Genetic differentiation between low- and high-latitude Chinese populations (top) and between US and all Chinese populations (bottom) in 50-kb sliding windows. Numbers on the x and y axes are the chromosome numbers and F_{ST} values, respectively.

(B) Manhattan plot for variants associated with precipitation seasonality (BIO15) (top) and soil temperature (BIO30) (bottom). The x and y axes indicate the chromosome numbers and the significance of environmental factor–associated SNPs, respectively. GO terms associated with the candidate genes are indicated with black arrows. The full list of GO terms can be found in Supplemental Tables S10–S12.

Chinese groups (all Chinese, high latitude, and low latitude) showing relatively low individual-level expression divergence compared with the three US groups. These observations suggest that differential expression of protein-coding genes is less likely to be a major mechanism associated with the phenotypic divergence observed in common garden experiments.

Identification of candidate genes under selection during invasion processes

Our population genomic inferences indicated that natural selection may have acted on Chinese cordgrass populations during their invasion processes. We next used three strategies to identify candidate genes under selection. First, we calculated pairwise F_{ST} values between native US and low- and high-latitude introduced Chinese populations. Our results showed that, although all Chinese populations were introduced from the same US source populations, the low- and high-latitude populations had evolved highly divergent genomic regions (HDGRs), both relative to each other and to the US source populations (Figure 4A and Supplemental Figure 12). A large number of HDGRs were specific to either low- or high-latitude Chinese populations, presumably owing to independent selection acting on distinct genomic regions during the expansion processes. Candidate genes identified within these HDGRs were functionally associated with photosynthesis (*i.e.*, regulation of photosynthesis), photomorphogenesis (*i.e.*, detection of light stimulus), plant growth (*i.e.*, response to auxin), development (*i.e.*, regulation of pollen tube growth and lateral root and flower development), and plant defense (Supplemental Table 10).

Second, we performed genome-wide scans to identify genomic regions associated with selective sweeps in each of these native US and introduced Chinese populations. Broadly consistent with the distribution pattern of HDGRs, the majority of the selective sweep regions (top 1% of the total and composite likelihood ratio > 1000) were specific to each of the populations (Supplemental Figure 13). In the highest-latitude population, TS, for example, 23 of the 31 chromosomes contained selective sweep regions. Protein-coding genes within selective sweep regions were functionally associated with cold acclimation, root development, photomorphogenesis, and response to defense and salt stress (Supplemental Figure 13; Supplemental

Table 11). Likewise, candidate genes under selection identified in the low-latitude population BH were functionally associated with photosynthesis (*i.e.*, photosystem II light-harvesting complex and chlorophyll and carotenoid biosynthesis), photomorphogenesis, and plant growth and development (*i.e.*, response to auxin and regulation of flower development). In the mid-latitude population QZ, which is geographically close to Luoyuan Bay (where cordgrass was initially introduced in China), candidate genes identified within selective sweep regions were functionally associated with circadian rhythm, photomorphogenesis, plant growth (*i.e.*, auxin biosynthetic process), and defense (response to jasmonic acid). By contrast, although selective sweep regions were also identified in native US populations, candidate genes under selection were not enriched in the functional pathways identified in the introduced Chinese populations.

Third, because the genomic scan identified group- and population-specific genes under selection, we performed genome-wide ecological association analysis to determine whether any genetic variants were correlated with environmental factors. Our results showed that several environmental factors related to temperature and precipitation, such as maximum temperature of the warmest month (BIO5), precipitation seasonality (BIO15), soil moisture (BIO24), and soil temperature (BIO30), were associated with some genetic variants (Figure 4B and Supplemental Figure 14). Candidate genes that contained these genetic variants were functionally enriched in a number of important pathways, including photosynthesis, photomorphogenesis, and root and floral organ development (Supplemental Figure 15; Supplemental Table 12). Given that these environmental factors vary gradually along the coast of China, it is likely that they were potential determinants that shaped genomic architecture following invasion processes. Finally, we determined which candidate genes were identified by more than one of our three approaches. A total of 7020 candidate genes (15.2% of the total) were identified by 2 or 3 approaches (Supplemental Table 13). In particular, 104 (71.2%) of the 146 flowering-related genes were identified as candidate genes, 19 of which were identified by 2 or 3 approaches.

Genetic variants, gene expression, and structural differences in flowering-regulatory genes

Phenotypic comparisons revealed that high-latitude Chinese populations had earlier flowering times (*i.e.*, first day of flowering and peak day of flowering) than low-latitude Chinese and native US populations (Figure 2D). Our population genomic inferences identified functionally important pathways that were potentially correlated with these adaptive phenotypes, such as photomorphogenesis, gravitropism, and flower and meristem development (Supplemental Tables 10–12). On the basis of these findings, we next asked whether high-latitude Chinese populations harbored distinct genetic variants in flowering-related genes compared with low-latitude Chinese and native US populations. Our results showed that several genes in the flowering-regulatory network, such as *PhyA, MADS50, MADS51, and MADS56*, were highly divergent between native US and introduced Chinese populations (Figure 5A; Supplemental Table 14). We also identified several highly divergent flowering-related genes between high- and low-latitude Chinese populations, including *MADS51*, *ELF3*, *GI*, and *DTH2*. For example, high-latitude populations harbored several non-synonymous mutations in *DTH2* and *ELF3* compared with low-latitude Chinese and native US populations (Figure 5A and Supplemental Data 1). However, we did not observe significant differential expression of these candidate genes between native US and introduced Chinese populations or between high- and low-latitude Chinese populations (Supplemental Figure 16; Supplemental Table 15). Non-synonymous mutations identified in these genes (*i.e.*, *DTH2*) resulted in structural changes between the encoded proteins of earlier flowering (high-latitude Chinese populations) and later flowering accessions (low-latitude Chinese and native US populations) (Figure 5B and Supplemental Figure 17).

Discussion

Invasive alien species are those that have been introduced or spread outside of their natural past or present distribution range (Zenetos et al., 2010). To survive, alien species must cope with the growth–defense trade-off in novel environments. As a consequence, introduced alien species commonly show reduced damage from specialist herbivores and pathogens in their non-native ranges (Ebeling et al., 2008; Bieker et al., 2022). In turn, reallocation of resources due to relaxed selection for defense leads to EICA (Blossey and Notzold, 1995; Callaway et al., 2022). Broadly consistent with these predictions, introduced Chinese smooth cordgrass shows increased resistance but decreased tolerance to generalist herbivores compared with native US populations (Ju et al., 2019). Here, our phenotypic comparisons showed that, compared with native US populations, introduced Chinese populations have evolved advantageous vegetative and reproductive traits such as greater plant biomass, more ramets, and earlier flowering time. Rapid growth rate, greater plant biomass, and increased fecundity are common competitive traits that have been observed in diverse invasive alien species relative to native species (Callaway et al., 2022). Rapid evolution of strong competitive ability but decreased tolerance to generalists indicates the possibility of reallocation of resources from defense to growth during invasion processes along the coast of China. It is interesting that, in the salt marshes of North America, native smooth cordgrass co-occurs with invasive *Phragmites* species in the high intertidal zone (Langston et al., 2022). Along the coastal regions of China, however, introduced smooth cordgrass either competes with native *Phragmites* species in the high intertidal zone or colonizes the low intertidal zone alone (Wang, 2007; Gan et al., 2009). Different roles in native–invasive interactions may have resulted in the distinct plant performance of smooth cordgrass in North America and China. Together, these observations support the EICA hypothesis that invasive species exhibit greater competitive effects in non-native environments.

Introduced smooth cordgrass populations exhibit latitudinal clines in plant height, clonal propagation, number of spikelets, and seed set in natural habitats along the coast of China (Liu et al., 2016, 2020). The same latitudinal gradient is observed in smooth cordgrass populations along the eastern coast of its native range in North America (Pennings and Bertness, 2001; Richards et al., 2005; Zerebecki et al., 2021). However, our common garden experiment revealed high phenological

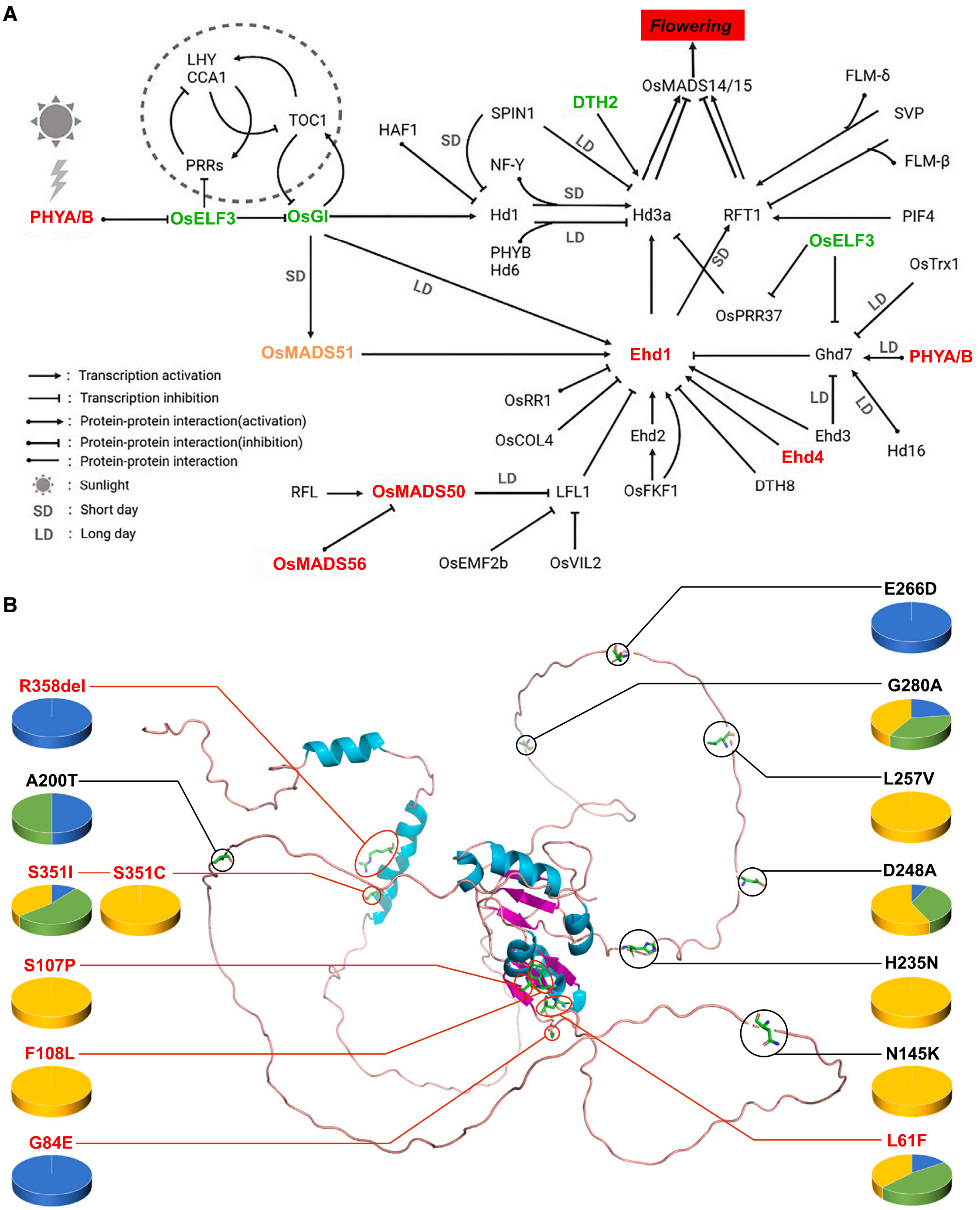

Figure 5. Regulatory network of flowering time and structure of the protein encoded by *DTH2*.

(A) Candidate genes involved in the regulation of flowering time in the grass family. Genes in red, green, and yellow show high genetic differentiation between native US and introduced Chinese populations, between high- and low-latitude Chinese populations, and in both comparisons, respectively. **(B)** Simulation of DTH2 protein structure. All non-synonymous mutations identified in US and Chinese accessions are indicated. Amino acid mutations that result in large structural changes are highlighted in red. Orange, blue, and green in the pie chart indicate native US and high- and low-latitude Chinese accessions, respectively.

divergence (*i.e.*, days to first flowering) between low- and high-latitude Chinese populations. A similar phenomenon was also observed in previous studies, in which no latitudinal clines were observed in common garden experiments (Liu et al., 2016, 2020). Differences in plant performance between common garden and natural conditions have been explained by the phenotypic plasticity of smooth cordgrass in its non-native range (Liu et al., 2017). However, phenotypic plasticity is the ability of the same genotype, which harbors the same genetic variants, to produce distinct phenotypes in response to

different environmental conditions (Sommer, 2020). Yet all introduced Chinese populations originated from the same US source populations and were primarily grown in a low-latitude region of China (FZ: FuZhou, Fujian province) (Xia et al., 2020). Our population genomic inferences clearly revealed high genetic differentiation between early- (high-latitude) and late-flowering (low-latitude) populations. However, gene expression patterns of the common garden samples were not associated with this phenological divergence. Given that both the high- and low-latitude Chinese populations originated from the same source populations, the rapid evolution of fitness traits (*i.e.*, earlier flowering time) in introduced Chinese populations is more likely to have been determined by distinct genetic variants during secondary or tertiary intentional introductions along the coast of China.

Our phenotypic comparisons demonstrated that reallocation of resources from defense to growth and development, together with independent secondary introductions along the coast of China, promoted the evolution of high competitive ability in introduced Chinese populations. These attributes provide an ideal system in which to investigate the genetic bases underpinning the invasion success of smooth cordgrass. The propagule pressure hypothesis posits that invasion success of an alien species is determined by the total numbers of introduced individuals and events in non-native environments (Lockwood et al., 2005; Simberloff, 2009; Blackburn et al., 2013; Jeschke et al., 2014). Alien species that are introduced from diverse source populations tend to show high genetic diversity and phenotypic plasticity, which are beneficial for rapid adaptation in the early stages of invasion (Allendorf and Lundquist, 2003; Ghalambor et al., 2007; Bock et al., 2016). In China, high genetic diversity and mixed population structure have been proposed as important determinants of the invasion success of smooth cordgrass along coastal regions (Wang et al., 2012; Chen et al., 2015; Qiao et al., 2019; Shang et al., 2019). However, these observations relied mainly on several cpDNA (chloroplast DNA) fragments and microsatellites. In this study, our population genomic inferences based on genome-wide SNPs clearly revealed high π and genetic mixture in introduced Chinese populations, supporting the previously proposed hypothesis that high genetic diversity is an important contributor to the invasion success of smooth cordgrass in China. In addition, both intra- and interspecific hybridization are crucial evolutionary driving forces that promote species adaptation and diversification (Huxel, 1999; Hall et al., 2006; Soltis and Soltis, 2009; Wang et al., 2022, 2023; Wu et al., 2022). Under this hypothesis, genetic mixture (or intraspecific hybridization) among distinct US source populations is a potential mechanism that may have contributed to the successful invasion of smooth cordgrass along the coast of China. However, the insufficient sample size of the US source populations limits further exploration of exactly how this intraspecific hybridization shaped the mixed structure of introduced Chinese populations. On the other hand, rapid evolution of self-fertility has been proposed as an adaptive mechanism underlying the invasion of smooth cordgrass along the Pacific coast of North America (Daehler and Strong, 1997; Daehler, 1998; 1999; Davis et al., 2004; Davis, 2005; Sloop et al., 2009). However, our Fis estimates indicate that changes in mating system, such as from outcrossing to self-fertility, are less likely to have been an underlying mechanism promoting rapid adaptation in coastal regions of China.

Consistent with the results of phenotypic comparisons, candidate genes under selection were functionally associated with flowering time (*i.e.*, photomorphogenesis and pollen and ovule development), photosynthesis (*i.e.*, chloroplast organization), plant growth (*i.e.*, response to auxin), and defense (*i.e.*, response to wounding). In the grass family, flowering time is initiated by a combination of light receptors (*i.e.*, *PhyA*, *PhyB*, and *Cry1*) and temperature sensors (*i.e.*, *PhyB*, *PIF4*, and *DTH8*), which interact with circadian clock genes to regulate floral meristem development (Cao et al., 2021; Wang et al., 2021; Zhou et al., 2021). In natural habitats along the coast of China, low-latitude populations of smooth cordgrass flower earlier than high-latitude populations (Chen et al., 2021). Our common garden experiments, however, revealed that the earlier flowering time of the high-latitude populations compared to the low-latitude Chinese and native US populations, might be a consequence of the shorter growing season of high-latitude populations. In cultivated rice, different alleles of the same gene, such as *Ehd4*, *Ghd7*, and *DTH2*, strongly influence flowering time along a latitudinal gradient (Wu et al., 2013; Zhang et al., 2015; Matsubara and Yano, 2018). A typical example is the *DTH2* gene, in which artificial selection acting on different *A4* alleles is thought to be associated with local adaptation of cultivated rice during the migration from low- to high-latitude regions (Wu et al., 2013). Here, our genetic analysis also identified several flowering-regulating genes that displayed high genetic differentiation between native US and introduced Chinese populations (*i.e.*, *Ehd4*, *MADS50*, *MADS51*, *MADS56*, and *PHYA*) or between low- and high-latitude Chinese populations (*i.e.*, *DTH2*, *MADS51*, *GI*, and *ELF3*). Some of these candidate genes (*i.e.*, *DTH2*) harbored non-synonymous mutations that were specific to either early- or late-flowering populations and were associated with changes in protein structure, suggesting that these causal genetic variants may have contributed to the adaptive evolution of introduced populations along the coast of China. However, because smooth cordgrass is a non-model species, we are not able to examine the molecular functions of these candidate genes. Further studies focusing on functional validation would provide solid evidence for their roles in the invasion success of smooth cordgrass.

In summary, smooth cordgrass is an invasive species in coastal ecosystems, whose invasion has serious ecological consequences for intertidal mudflats and salt marshes. Here, phenotypic comparisons showed that both low- and high-latitude Chinese populations have evolved competitive traits during secondary intentional introductions along the coast of China. An integrated investigation based on genomic and transcriptomic data revealed distinct evolutionary trajectories and genes under selection in the two introduced Chinese groups. These phenotypic and genomic features suggest that prioritization of resource allocation toward growth–defense trade-off and high genome plasticity are the underlying mechanisms of invasion success in smooth cordgrass. Our results also showed that expanded and specific gene families identified in smooth cordgrass were functionally associated with salinity and submergence tolerance. Salinity and flooding are major abiotic stresses that limit crop growth and productivity in many areas

of the world. Candidate genes for salt and submergence tolerance provide valuable genetic resources for further improvement of cereal crops.

METHODS

Plant materials and DNA and RNA extraction

A total of 115 accessions from 18 field populations were collected from the US and China (Supplemental Table 16). For the 13 Chinese populations, 5 accessions were sampled from non-overlapping plots (5 × 5 m) that were more than 1 km away from each other. Of the 5 US populations, 4–16 accessions were collected from each of the source and non-source populations. All collected accessions were subjected to deep whole-genome resequencing. One accession from the US Sapelo Island (S13) population was selected to assemble the reference genome. Seventy-five accessions were chosen for transcriptome sequencing of leaf and root tissues. All plants used in this study were grown in a greenhouse at Fudan University (Shanghai, China). Genomic DNA was extracted from leaf tissue using a modified cetyltrimethylammonium bromide protocol (Murray and Thompson, 1980). Total RNA was extracted from fresh leaf and root tissues of each accession using RNA extraction kits (Tiangen, Beijing, China).

Phenotyping of vegetative and reproductive traits

Twelve vegetative and reproductive traits were measured for all 115 US and Chinese accessions, including plant height, shoot number, aboveground biomass, fibrous root biomass, rhizome biomass, belowground biomass, first flowering day, and percentage of ramets that flowered (Supplemental Table 17). In brief, 15 seedlings were randomly chosen from each of the 18 populations and planted in plastic pots (16 cm in diameter and 17.5 cm in height) containing a mixture of vermiculite and soil (1:3). All selected seedlings were grown in a greenhouse with 10‰ salinity concentration. The first flowering day was determined by counting the number of days from root sprouting to the appearance of flowering. Plant height, number of ramets, and fibrous roots were estimated at the end of the growing season. All plants were harvested independently for measurement of aboveground and belowground biomass, including fibrous root biomass and rhizome biomass. All collected plants were dried to constant weight in an oven at 55°C and then weighed with an electronic balance. Effective tillers were estimated by calculating the ratio of flowering tillers at the end of the growing season. Plant height of each accession was measured for the highest ramet. ANOVA was used to examine phenotypic differences between native and introduced populations with SPSS Statistics (v.20.0) (IBM SPSS, 2011).

Genome assembly and annotation

The genome size of *S. alterniflora* was estimated using flow cytometry. In brief, fresh leaves were placed in 0.8 ml of ice-cold mGb dissociation solution and cut into small pieces. The leaf sample was placed on ice in dissociation solution for 10 min and filtered through a 40-μm aperture filter screen to obtain the nuclear suspension. An appropriate volume of precooled propidium iodide and RNAase solution was added to the nuclear suspension, and the mixture was placed on ice in the dark for 0.5–1 h. The prepared sample was used for genome size estimation using a BD FACSCalibur flow cytometer (Becton Dickinson, San Jose, CA, USA) with tomato (genome size = 900 Mb) as the internal reference. Genome features were estimated on the basis of Illumina short reads using Jellyfish (Marçais and Kingsford, 2011). A total of 218.01 Gb (127.33× coverage) Illumina short reads were generated using the Illumina NovaSeq 6000 platform (Illumina, CA, USA). The karyotype was examined by counting chromosome numbers using fluorescence *in situ* hybridization. A young root tip was incubated in ice water for 24 h and fixed with 90% acetic acid for 10 min. The preserved root was then dissociated in 0.02 M HCl solution overnight. The karyotype of the root sample was examined under a fluorescence microscope (Leica, DM2500).

We performed deep sequencing of the *S. alterniflora* genome using the PacBio Sequel (PacBio) and Nanopore (ONT) platforms. PacBio and Nanopore long reads were used to assemble a draft genome using Hi-Fiasm (v.0.12) (Cheng et al., 2021). Chromosome scaffolding was performed by Hi-C using LACHESIS (Burton et al., 2013). A total of 251.49 Gb (154.29× genome coverage) Hi-C short reads were generated on the Illumina NovaSeq 6000 platform. Genome completeness was assessed by CEGMA v.2.5 (Parra et al., 2007) and BUSCO v.2.0 (Simao et al., 2015).

Protein-coding genes were annotated by *de novo* prediction, homology search, and transcript-based assembly. The *de novo* gene models were predicted using Genscan (Burge and Karlin, 1997), Augustus v.2.4 (Stanke and Waack, 2003), GlimmerHMM v.3.0.4 (Majoros et al., 2004), GeneID v.1.4 (Blanco et al., 2007), and SNAP (v.2006-07-28) (Korf, 2004). Homolog-based annotation was performed using GeMoMa v.1.3.1 (Keilwagen et al., 2016, 2018), and transcript-based prediction was performed using TransDecoder v.2.0 (Haas and Papanicolaou, 2017) and GeneMarkS-T v.5.1 (Tang et al., 2015). Gene models from these three approaches were combined using EVM v.1.1.1 (Haas et al., 2008). Gene functions were assigned according to the best match of alignments to the NCBI Non-Redundant (Marchler-Bauer et al., 2010), Eukaryotic Orthologous Groups (Koonin et al., 2004), GO (Dimmer et al., 2012), Kyoto Encyclopedia of Genes and Genomes (Kanehisa and Goto, 2000), and Translated EMBL Nucleotide Sequence Database (Boeckmann et al., 2003) using BLAST v.2.2.31 (Altschul et al., 1990). Repetitive sequences were identified using LTR_FINDER (v.1.0.6) (Xu and Wang, 2007) and RepeatScout (v.1.0.6) (Price et al., 2005) and further annotated using RepeatMasker (v.1.0.6) (Chen, 2004). BLASTn was used with the the Rfam database (Griffiths-Jones et al., 2005) to identify microRNAs and rRNAs by genome-wide comparison. tRNAs were predicted with tRNAscan-SE (v.1.3.1) (Lowe and Eddy, 1997). Expansion and contraction of gene families were inferred using Café 5 (Mendes et al., 2020). The genome sequences of seven other species were obtained from Phytozome (https://phytozome-next.jgi.doe.gov) and the Germplasm Bank of Wild Species (http://www.genobank.org/bamboo#2).

DNA and RNA sequencing, SNP calling, and gene expression

High-quality genomic DNA extracted from fresh leaves was used for 350-bp Illumina library preparation following the manufacturer's protocol. High-throughput sequencing was performed on the Illumina NovaSeq 6000 platform. High-quality paired-end short reads from resequenced samples were mapped onto the reference genome using BWA v.0.7.12 (Li, 2013) with the parameters "mem -t 4 -k 32 –M." PCR and optical duplicates were identified and removed using SAMtools v.1.3.1(Li et al., 2009). SNPs were identified using a UnifiedGenotyper approach as implemented in the Genome Analysis Toolkit package (v.3.7-0-gcfedb67) (McKenna et al., 2010). Only high-quality SNPs that passed quality control (mapping quality >30, read depth $\geq$5, frequency of missing allele >0.05, and allele frequency >0.05) were retained for subsequent population genomic inference, including analysis of π, genetic differentiation, and environmental factor associations. SNP datasets used for phylogenetic and ancestral genetic cluster analysis were further filtered using PLINK v.1.90b6.26 (Purcell et al., 2007) with the parameters "indep-pairwise, 50 10 0.2." Functional annotation of candidate SNPs was performed with the ANNOVAR package (v.2013-06-21) (Wang et al., 2010), including exon regions, intron regions, splicing sites, upstream and downstream regions, and intergenic regions.

Total RNA was isolated from two tissues using RNA extraction kits (Tiangen) and quantified with a NanoDrop 2000C spectrophotometer (Thermo Scientific, Waltham, MA, USA). Paired-end sequencing was performed using the Illumina NovaSeq 6000 platform. Gene transcript levels were

estimated as fragments per kilobase of transcript per million mapped reads using StringTie v.2.1.4 (Pertea et al., 2015). DESeq2 (Love et al., 2014) was used to identify DEGs. Genes with at least a 2-fold difference in expression ($p < 0.05$) were defined as DEGs. Functional annotation of the DEGs was performed using GO databases (Consortium, 2004) and visualized with the R package clusterProfiler v.4.6.0 (Wu et al., 2021). The DEG heatmap was plotted using the R package pheatmap v.1.0.12 (Kolde, 2019).

Phylogeny, population structure, and demographic history

Phylogenetic relationships of the 115 accessions were inferred from high-quality SNPs using a neighbor-joining analysis in MEGA v.10.2.3 (Kumar et al., 2012) with the Kimura 2-parameter model and 1000 bootstrap replicates. The phylogenetic tree was visualized using the online tool ITOL (https://itol.embl.de). The same SNP dataset was also used to infer the genetic structure of the US and Chinese populations. Population genetic structure was inferred using ADMIXTURE v.1.3.0 (Alexander et al., 2009). The best genetic cluster was determined by cross-validation for K values from 1 to 7. The $K = 4$ value was chosen because clusters maximized the marginal likelihood. LD decay of native US, introduced high-latitude, and introduced low-latitude groups was calculated using PopLDdecay v.3.40 (Zhang et al., 2019). Squared correlation coefficients between pairwise SNPs are shown in the LD decay plot. Genome-wide π and Fis values were calculated with at least five individuals using VCFtools v.0.1.17 (Danecek et al., 2011) and PLINK v.1.90b6.26 (Purcell et al., 2007). Isolation by distance among the native US and invasive Chinese populations was estimated using the R package vegan (Oksanen et al., 2022). Population split and mixture events were inferred on the basis of allele frequency using Treemix (Pickrell and Pritchard, 2012).

Identification of candidate genes associated with local adaptation

Candidate genes potentially under selection were identified by population genomic inference and environmental factor association. We identified genomic regions under selection using SweeD v.4.0.0 (Pavlidis et al., 2013) with sliding windows of 50 kb. The top 1% of regions with a composite likelihood ratio greater than 1000 were defined as selected regions. To identify HDGRs between native US and introduced Chinese populations, we calculated F_{ST} on the basis of genome-wide SNPs using VCFtools (v.0.1.17) (Danecek et al., 2011) with a 50-kb sliding window. The top 1% highest divergent genomic regions were considered HDGRs. The program qqman (v.0.1.8) (Turner, 2014) was used to plot F_{ST} values.

Thirty-eight environmental variables related to temperature, precipitation, radiation, and humidity were retrieved from WorldClim (https://www.worldclim.org), ERA5-Land (https://developers.google.com/earth-engine/datasets/catalog/ECMWF_ERA5_LAND_HOURLY?hl=en), and TerraClimate (https://www.climatologylab.org/terraclimate.html). To explore the contribution of environmental variables to standing genetic variation, we used a univariate latent-factor linear mixed model implemented in the R package LEA (v.3.2.0) (Frichot and François, 2015) to investigate associations between allele frequencies and the 38 environmental variables. On the basis of the population genetic structure evaluated with the program snmf, we ran the latent-factor linear mixed model with eight latent factors to increase the power to detect true associations. GO term enrichment analysis was performed with the R package clusterProfiler v.4.6.0 (Wu et al., 2021). Protein structures encoded by selected genes were simulated using AlphaFold 2 (https://www.bkunyun.com) (Jumper et al., 2021).

DATA AVAILABILITY

All data supporting the findings of this study are available in the paper and the supplemental information files. Raw sequence data and genome assemblies have been deposited at the National Genomics Data Center under project numbers PRJCA016449 and PRJCA016599.

SUPPLEMENTAL INFORMATION

Supplemental information is available at *Plant Communications Online*.

FUNDING

This study was supported by the National Key Research and Development Program of China (2022YFC2601100 to B.L.) and the Natural Science Foundation of China (32030067 to J.-H.W., 31970235 to L.-F.L., 32171661 to R.-T.J., and 31961133028 to B.L.).

AUTHOR CONTRIBUTIONS

L.-F.L., R.-T.J., J.-H.W., J.Y., M.L.A., A.S., and B.L. conceived this project. L.-F.L., Y.H., and B.L. designed and supervised the project. Y.H., X.-F.W., T.-Y.L., and Y.G. conducted the experiments and analyzed the data. Y.H., X.-F.W., R.-T.J., J.Y., J.-H.W., L.-F.L., M.L.A., A.S., and B.L. conceptualized and drafted the manuscript. All authors discussed the results and approved the manuscript.

ACKNOWLEDGMENTS

No conflict of interest is declared.

Received: September 8, 2023
Revised: December 18, 2023
Accepted: January 10, 2024
Published: January 13, 2024

REFERENCES

Ainouche, M.L., Baumel, A., Salmon, A., and Yannic, G. (2004). Hybridization, polyploidy and speciation in *Spartina* (Poaceae). New Phytol. **161**:165–172.

Aïnouche, M.L., Fortune, P.M., Salmon, A., Parisod, C., Grandbastien, M.A., Fukunaga, K., Ricou, M., and Misset, M.T. (2009). Hybridization, polyploidy and invasion: lessons from *Spartina* (Poaceae). Biol. Invasions **11**:1159–1173.

Alber, M., Swenson, E.M., Adamowicz, S.C., and Mendelssohn, I.A. (2008). Salt Marsh Dieback: An overview of recent events in the US. Estuarine, Coastal and Shelf. Sci **80**:1–11.

Alexander, D.H., Novembre, J., and Lange, K. (2009). Fast model-based estimation of ancestry in unrelated individuals. Genome Res. **19**:1655–1664.

Allendorf, F.W., and Lundquist, L.L. (2003). Introduction: population biology, evolution, and control of invasive species. Conserv. Biol. **17**:24–30.

Altschul, S.F., Gish, W., Miller, W., Myers, E.W., and Lipman, D.J. (1990). Basic local alignment search tool. J. Mol. Biol. **215**:403–410.

An, S.Q., Gu, B.H., Zhou, C.F., Wang, Z.S., Deng, Z.F., Zhi, Y.B., Li, H.L., Chen, L., Yu, D.H., and Liu, Y.H. (2007). *Spartina* invasion in China: implications for invasive species management and future research. Weed Res. **47**:183–191.

Angelini, C., and Silliman, B.R. (2012). Patch size-dependent community recovery after massive disturbance. J. Ecol. **93**:101–110.

Ayres, D.R., Grotkopp, E., Zaremba, K., Sloop, C.M., Blum, M.J., Bailey, J.P., Anttila, C.K., and Strong, D.R. (2008). Hybridization between invasive *Spartina densiflora* (Poaceae) and native *S. foliosa* in San Francisco Bay, California, USA. Am. J. Bot. **95**:713–719.

Barbier, E.B., Hacker, S.D., Kennedy, C., Koch, E.W., Stier, A.C., and Silliman, B.R. (2011). The value of estuarine and coastal ecosystem services. Ecol. Monogr. **81**:169–193.

Barrett, S.C.H. (2015). Foundations of invasion genetics: the Baker and Stebbins legacy. Mol. Ecol. **24**:1927–1941.

Bieker, V.C., Battlay, P., Petersen, B., Sun, X., Wilson, J., Brealey, J.C., Bretagnolle, F., Nurkowski, K., Lee, C., Barreiro, F.S., et al. (2022). Uncovering the genomic basis of an extraordinary plant invasion. Sci. Adv. **8**, eabo5115.

Blackburn, T.M., Prowse, T.A.A., Lockwood, J.L., and Cassey, P. (2013). Propagule pressure as a driver of establishment success in deliberately introduced exotic species: fact or artefact? Biol. Invasions **15**:1459–1469.

Blanco, E., Parra, G., and Guigó, R. (2007). Using geneid to identify genes. Curr. Protoc. Bioinf. **18**:e56.

Blossey, B., and Notzold, R. (1995). Evolution of increased competitive ability in invasive nonindigenous plants: a hypothesis. J. Ecol. **83**:887–889.

Bock, D.G., Caseys, C., Cousens, R.D., Hahn, M.A., Heredia, S.M., Hübner, S., Turner, K.G., Whitney, K.D., and Rieseberg, L.H. (2016). What we still don't know about invasion genetics. Mol. Ecol. **24**:2277–2297.

Boeckmann, B., Bairoch, A., Apweiler, R., Blatter, M.C., Estreicher, A., Gasteiger, E., Martin, M.J., Michoud, K., O'Donovan, C., Phan, I., et al. (2003). The SWISS-PROT protein knowledgebase and its supplement TrEMBL in 2003. Nucleic Acids Res. **31**:365–370.

Burge, C., and Karlin, S. (1997). Prediction of complete gene structures in human genomic DNA. J. Mol. Biol. **268**:78–94.

Burton, J.N., Adey, A., Patwardhan, R.P., Qiu, R., Kitzman, J.O., and Shendure, J. (2013). Chromosome-scale scaffolding of de novo genome assemblies based on chromatin interactions. Nat. Biotechnol. **31**:1119–1125.

Callaway, R.M., Lucero, J.E., Hierro, J.L., and Lortie, C.J. (2022). The EICA is dead? Long live the EICA. Ecol. Lett. **25**:2289–2302.

Cao, S., Luo, X., Xu, D., Tian, X., Song, J., Xia, X., Chu, C., and He, Z. (2021). Genetic architecture underlying light and temperature mediated flowering in *Arabidopsis*, rice, and temperate cereals. New Phytol. **230**:1731–1745.

Chen, N. (2004). Using Repeat Masker to identify repetitive elements in genomic sequences. Curr. Protoc. Bioinf. **5**:4.

Chen, X., Liu, W., Pennings, S.C., and Zhang, Y. (2021). Plasticity and selection drive hump-shaped latitudinal patterns of flowering phenology in an invasive intertidal plant. J. Ecol. **102**, e03311.

Chen, X.Y., Wang, X.Y., Jiao, J., and Schmid, B. (2015). Complementarity effects do not necessarily result in significant transgressive over-performance in mixtures. Biol. Invasions **17**:529–535.

Cheng, H., Concepcion, G.T., Feng, X., Zhang, H., and Li, H. (2021). Haplotype-resolved de novo assembly using phased assembly graphs with hifiasm. Nat. Methods **18**:170–175.

Chung, C.H., Zhuo, R.Z., and Xu, G.W. (2004). Creation of *Spartina* plantations for reclaiming Dongtai, China, tidal flats and offshore sands. Ecol. Eng. **23**:135–150.

Consortium, G.O, Harris, M.A., Clark, J., Ireland, A., Lomax, J., Ashburner, M., Foulger, R., Eilbeck, K., Lewis, S., Marshall, B., Mungall, C., et al. (2004). The Gene Ontology (GO) database and informatics resource. Nucleic Acids Res. **32**:D258–D261.

Costanza, R. (1999). The ecological, economic, and social importance of the oceans. Ecol. Econ. **31**:199–213.

Daehler, C.C. (1998). Variation in self-fertility and the reproductive advantage of self-fertility for an invading plant (*Spartina alterniflora*). Evol. Ecol. **12**:553–568.

Daehler, C.C. (1999). Inbreeding depression in smooth cordgrass (*Spartina alterniflora*, Poaceae) invading San Francisco Bay. Am. J. Bot. **86**:131–139.

Daehler, C.C., and Strong, D.R. (1997). Reduced herbivore resistance in introduced smooth cordgrass (*Spartina alterniflora*) after a century of herbivore-free growth. Oecologia **110**:99–108.

Danecek, P., Auton, A., Abecasis, G., Albers, C.A., Banks, E., DePristo, M.A., Handsaker, R.E., Lunter, G., Marth, G.T., Sherry, S.T., et al. (2011). The variant call format and VCFtools. Bioinformatics **27**:2156–2158.

Davis, H.G. (2005). r-Selected traits in an invasive population. Evol. Ecol. **19**:255–274.

Davis, H.G., Taylor, C.M., Lambrinos, J.G., and Strong, D.R. (2004). Pollen limitation causes an Allee effect in a wind-pollinated invasive grass (*Spartina alterniflora*). Proc. Natl. Acad. Sci. USA **101**:13804–13807.

Dimmer, E.C., Huntley, R.P., Alam-Faruque, Y., Sawford, T., O'Donovan, C., Martin, M.J., Bely, B., Browne, P., Mun Chan, W., Eberhardt, R., et al. (2012). The UniProt-GO annotation database in 2011. Nucleic Acids Res. **40**:D565–D570.

Doherty, T.S., Glen, A.S., Nimmo, D.G., Ritchie, E.G., and Dickman, C.R. (2016). Invasive predators and global biodiversity loss. Proc. Natl. Acad. Sci. USA **113**:11261–11265.

Doorduin, L.J., and Vrieling, K. (2011). A review of the phytochemical support for the shifting defense hypothesis. Phytochemistry Rev. **10**:99–106.

Ebeling, S.K., Hensen, I., and Auge, H. (2008). The invasive shrub *Buddleja davidii* performs better in its introduced range. Divers. Distrib. **14**:225–233.

Ehrenfeld, J.G. (2003). Effects of Exotic Plant Invasions on Soil Nutrient Cycling Processes. Ecosystems **6**:503–523.

Frichot, E., and François, O. (2015). LEA: An R package for landscape and ecological association studies. Methods Ecol. Evol. **6**:925–929.

Gan, X., Cai, Y., Choi, C., Ma, Z., Chen, J., and Li, B. (2009). Potential impacts of invasive *Spartina alterniflora* on spring bird communities at Chongming Dongtan, a Chinese wetland of international importance. Estuar. Coast Shelf Sci. **83**:211–218.

Ghalambor, C.K., McKay, J.K., Carroll, S.P., and Reznick, D.N. (2007). Adaptive versus non-adaptive phenotypic plasticity and the potential for contemporary adaptation in new environments. Funct. Ecol. **21**:394–407.

Griffiths-Jones, S., Moxon, S., Marshall, M., Khanna, A., Eddy, S.R., and Bateman, A. (2005). Rfam: annotating non-coding RNAs in complete genomes. Nucleic Acids Res. **33**:D121–D124.

Gurevitch, J., and Padilla, D.K. (2004). Are invasive species a major cause of extinctions? Trends Ecol. Evol. **19**:470–474.

Haas, B.J., and Papanicolaou, A. (2017). TransDecoder (Find Coding Regions within Transcripts). https://github.com/TransDecoder/TransDecoder.

Haas, B.J., Salzberg, S.L., Zhu, W., Pertea, M., Allen, J.E., Orvis, J., White, O., Buell, C.R., and Wortman, J.R. (2008). Automated eukaryotic gene structure annotation using EVidenceModeler and the Program to Assemble Spliced Alignments. Genome Biol. **9**:R7–R22.

Hall, R.J., Hastings, A., and Ayres, D.R. (2006). Explaining the explosion: modelling hybrid invasions. Proceeding B **273**:1385–1389.

He, Q., and Silliman, B.R. (2019). Climate change, human Impacts, and coastal Ecosystems in the Anthropocene. Curr. Biol. **29**:R1021–R1035.

Hensel, M.J.S., Silliman, B.R., van de Koppel, J., Hensel, E., Sharp, S.J., Crotty, S.M., and Byrnes, J.E.K. (2021). A large invasive consumer reduces coastal ecosystem resilience by disabling positive species interactions. Nat. Commun. **12**:6290.

Herbert-Read, J.E., Thornton, A., Amon, D.J., Birchenough, S.N.R., Côté, I.M., Dias, M.P., Godley, B.J., Keith, S.A., McKinley, E., Peck, L.S., et al. (2022). A global horizon scan of issues impacting

marine and coastal biodiversity conservation. Nat. Ecol. Evol. **6**:1262–1270.

Huxel, G.R. (1999). Rapid displacement of native species by invasive species: effects of hybridization. Biol. Conserv. **89**:143–152.

IBM SPSS. (2011). IBM SPSS Statistics for Windows, Version 20.0 (IBM Corp).

Jackson, J.B., Kirby, M.X., Berger, W.H., Bjorndal, K.A., Botsford, L.W., Bourque, B.J., Bradbury, R.H., Cooke, R., Erlandson, J., Estes, J.A., et al. (2001). Historical overfishing and the recent collapse of coastal ecosystems. Science **293**:629–637.

Jeschke, J.M., Bacher, S., Blackburn, T.M., Dick, J.T.A., Essl, F., Evans, T., Gaertner, M., Hulme, P.E., Kühn, I., Mrugała, A., et al. (2014). Defining the impact of non-native species. Conserv. Biol. **28**:1188–1194.

Ju, R.T., Ma, D., Siemann, E., Liu, X., Wu, J.H., and Li, B. (2019). Invasive *Spartina alterniflora* exhibits increased resistance but decreased tolerance to a generalist insect in China. J. Pest. Sci. **92**:823–833.

Jumper, J., Evans, R., Pritzel, A., Green, T., Figurnov, M., Ronneberger, O., Tunyasuvunakool, K., Bates, R., Žídek, A., Potapenko, A., et al. (2021). Highly accurate protein structure prediction with AlphaFold. Nature **596**:583–589.

Kanehisa, M., and Goto, S. (2000). KEGG: kyoto encyclopedia of genes and genomes. Nucleic Acids Res. **28**:27–30.

Keilwagen, J., Hartung, F., Paulini, M., Twardziok, S.O., and Grau, J. (2018). Combining RNA-seq data and homology-based gene prediction for plants, animals and fungi. BMC Bioinf. **19**:1–12.

Keilwagen, J., Wenk, M., Erickson, J.L., Schattat, M.H., Grau, J., and Hartung, F. (2016). Using intron position conservation for homology-based gene prediction. Nucleic Acids Res. **44**:e89.

Kolde, R. (2019). Pheatmap: Pretty Heatmaps. R Package Version 1 0. 12. https://CRAN.R-project.org/package=pheatmap.

Koonin, E.V., Fedorova, N.D., Jackson, J.D., Jacobs, A.R., Krylov, D.M., Makarova, K.S., Mazumder, R., Mekhedov, S.L., Nikolskaya, A.N., Rao, B.S., et al. (2004). A comprehensive evolutionary classification of proteins encoded in complete eukaryotic genomes. Genome Biol. **5**:R7–R28.

Korf, I. (2004). Gene finding in novel genomes. BMC Bioinf. **5**. 59–9.

Kowarik, I., Pyšek, P., and Rejmánek, M. (2012). The first steps towards unifying concepts in invasion ecology were made one hundred years ago: revisiting the work of the Swiss botanist Albert Thellung. Divers. Distrib. **18**:1243–1252.

Kumar, S., Stecher, G., Peterson, D., and Tamura, K. (2012). MEGA-CC: computing core of molecular evolutionary genetics analysis program for automated and iterative data analysis. Bioinformatics **28**:2685–2686.

Langston, A.K., Coleman, D.J., Jung, N.W., Shawler, J.L., Smith, A.J., Williams, B.L., Wittyngham, S.S., Chambers, R.M., Perry, J.E., and Kirwan, M.L. (2022). The effect of marsh age on ecosystem function in a rapidly transgressing marsh. Ecosystems **25**:252–264.

Li, H. (2013). Aligning sequence reads, clone sequences and assembly contigs with BWA-MEM. Preprint at arXiv. https://doi.org/10.48550/arXiv.1303.3997.

Li, H., Handsaker, B., Wysoker, A., Fennell, T., Ruan, J., Homer, N., Marth, G., Abecasis, G., and Durbin, R.; 1000 Genome Project Data Processing Subgroup (2009). The sequence Alignment/Map format and SAMtools. Bioinformatics **25**:2078–2079.

Liu, W., Strong, D.R., Pennings, S.C., and Zhang, Y. (2017). Provenance-by-environment interaction of reproductive traits in the invasion of *Spartina alterniflora* in China. J. Ecol. **98**:1591–1599.

Liu, W., Maung-Douglass, K., Strong, D.R., Pennings, S.C., and Zhang, Y. (2016). Geographical variation in vegetative growth and sexual reproduction of the invasive *Spartina alterniflora* in China. J. Ecol. **104**:173–181.

Liu, W., Zhang, Y., Chen, X., Maung-Douglass, K., Strong, D.R., and Pennings, S.C. (2020). Contrasting plant adaptation strategies to latitude in the native and invasive range of *Spartina alterniflora*. New Phytol. **226**:623–634.

Lockwood, J.L., Cassey, P., and Blackburn, T. (2005). The role of propagule pressure in explaining species invasions. Trends Ecol. Evol. **20**:223–228.

Love, M.I., Huber, W., and Anders, S. (2014). Moderated estimation of fold change and dispersion for RNA-seq data with DESeq2. Genome Biol. **15**:1–21.

Lowe, T.M., and Eddy, S.R. (1997). tRNAscan-SE: a program for improved detection of transfer RNA genes in genomic sequence. Nucleic Acids Res. **25**:955–964.

MacDougall, A.S., and Turkington, R. (2005). Are Invasive Species the Drivers or Passengers of Change in Degraded Ecosystems? J. Ecol. **86**:42–55.

Majoros, W.H., Pertea, M., and Salzberg, S.L. (2004). TigrScan and GlimmerHMM: two open source ab initio eukaryotic gene-finders. Bioinformatics **20**:2878–2879.

Marçais, G., and Kingsford, C. (2011). A fast, lock-free approach for efficient parallel counting of occurrences of k-mers. Bioinformatics **27**:764–770.

Marchler-Bauer, A., Lu, S., Anderson, J.B., Chitsaz, F., Derbyshire, M.K., DeWeese-Scott, C., Fong, J.H., Geer, L.Y., Geer, R.C., Gonzales, N.R., et al. (2010). CDD: a Conserved Domain Database for the functional annotation of proteins. Nucleic Acids Res. **39**:D225–D229.

Matsubara, K., and Yano, M. (2018). Genetic and Molecular Dissection of Flowering Time Control in Rice. Rice Genomics, Genetics and Breeding, 177–190.

McKee, K.L., Mendelssohn, I.A., D Materne, M., and M.. (2004). Acute salt marsh dieback in the Mississippi River deltaic plain: a drought-induced phenomenon? Global Ecol. Biogeogr. **13**:65–73.

McKenna, A., Hanna, M., Banks, E., Sivachenko, A., Cibulskis, K., Kernytsky, A., Garimella, K., Altshuler, D., Gabriel, S., Daly, M., et al. (2010). The Genome Analysis Toolkit: a MapReduce framework for analyzing next-generation DNA sequencing data. Genome Res. **20**:1297–1303.

Mendes, F.K., Vanderpool, D., Fulton, B., and Hahn, M.W. (2020). CAFE 5 models variation in evolutionary rates among gene families. Bioinformatics **36**:5516–5518.

Müller-Schärer, H., Schaffner, U., and Steinger, T. (2004). Evolution in invasive plants: implications for biological control. Trends Ecol. Evol. **19**:417–422.

Murray, M.G., and Thompson, W.F. (1980). Rapid isolation of high molecular weight plant DNA. Nucleic Acids Res. **8**:4321–4325.

Oksanen, J., Blanchet, F.G., Friendly, M., Kindt, R., Legendre, P., McGlinn, D., Minchin, P., O'hara, R., Simpson, G., and Solymos, P. (2022). Vegan: Community Ecology Package. R Package Version 2.5-7. https://CRAN.R-project.org/package=vegan.

Parra, G., Bradnam, K., and Korf, I. (2007). CEGMA: a pipeline to accurately annotate core genes in eukaryotic genomes. Bioinformatics **23**:1061–1067.

Pavlidis, P., Živkovic, D., Stamatakis, A., and Alachiotis, N. (2013). SweeD: likelihood-based detection of selective sweeps in thousands of genomes. Mol. Biol. Evol. **30**:2224–2234.

Pennings, S.C., and Bertness, M.D. (2001). Salt marsh communities. Marine community ecology **11**:289–316.

Pertea, M., Pertea, G.M., Antonescu, C.M., Chang, T.C., Mendell, J.T., and Salzberg, S.L. (2015). StringTie enables improved reconstruction of a transcriptome from RNA-seq reads. Nat. Biotechnol. **33**:290–295.

Pickrell, J.K., and Pritchard, J.K. (2012). Inference of population splits and mixtures from genome-wide allele frequency data. PLoS Genet. **8**, e1002967.

Powell, K.I., Chase, J.M., and Knight, T.M. (2011). A Synthesis of Plant Invasion Effects on Biodiversity across Spatial Scales. Am. J. Bot. **98**:539–548.

Price, A.L., Jones, N.C., and Pevzner, P.A. (2005). De novo identification of repeat families in large genomes. Bioinformatics **21**:i351–i358.

Purcell, S., Neale, B., Todd-Brown, K., Thomas, L., Ferreira, M.A.R., Bender, D., Maller, J., Sklar, P., de Bakker, P.I.W., Daly, M.J., et al. (2007). PLINK: a tool set for whole-genome association and population-based linkage analyses. Am. J. Hum. Genet. **81**:559–575.

Qiao, H., Liu, W., Zhang, Y., Zhang, Y.Y., and Li, Q.Q. (2019). Genetic admixture accelerates invasion via provisioning rapid adaptive evolution. Mol. Ecol. **28**:4012–4027.

Qin, P., Jin, M.D., and Xie, M. (1985). The comparison of community biomass among the three eotypes of Spartina alternifora on the beach of estuary Luoyuan Bay, Fujian. J. Nanjing Univ. Nat. Sci. 226–236.

Richards, C.L., Pennings, S.C., and Donovan, L.A. (2005). Habitat range and phenotypic variation in salt marsh plants. Plant Ecol. **176**:263–273.

Sakai, A.K., Allendorf, F.W., Holt, J.S., Lodge, D.M., Molofsky, J., With, K.A., Baughman, S., Cabin, R.J., Cohen, J.E., Ellstrand, N.C., et al. (2001). The Population Biology of Invasive Species. Annu. Rev. Ecol. Systemat. **32**:305–332.

Sax, D.F., Stachowicz, J.J., Brown, J.H., Bruno, J.F., Dawson, M.N., Gaines, S.D., Grosberg, R.K., HastingS, A., Holt, R.D., Mayfield, M.M., et al. (2007). Ecological and evolutionary insights from species invasions. Trends Ecol. Evol. **22**:465–471.

Shang, L., Li, L.F., Song, Z.P., Wang, Y., Yang, J., Wang, C.C., Qiu, S.Y., Huang, J.X., Nie, M., Wolfe, L.M., and Li, B. (2019). High genetic diversity with weak phylogeographic structure of the invasive *Spartina alterniflora* (Poaceae) in China. Front. Plant Sci. **10**:1467.

Simão, F.A., Waterhouse, R.M., Ioannidis, P., Kriventseva, E.V., and Zdobnov, E.M. (2015). BUSCO: assessing genome assembly and annotation completeness with single-copy orthologs. Bioinformatics **31**:3210–3212.

Simberloff, D. (2009). The Role of Propagule Pressure in Biological Invasions. Annu. Rev. Ecol. Evol. Syst. **40**:81–102.

Sloop, C.M., Ayres, D.R., and Strong, D.R. (2009). The rapid evolution of self-fertility in *Spartina* hybrids (*Spartina alterniflora* × *foliosa*) invading San Francisco Bay, CA. Biol. Invasions **11**:1131–1144.

Soltis, P.S., and Soltis, D.E. (2009). The role of hybridization in plant speciation. Annu. Rev. Plant Biol. **60**:561–588.

Sommer, R.J. (2020). Phenotypic plasticity: from theory and genetics to current and future challenges. Genetics **215**:1–13.

Stanke, M., and Waack, S. (2003). Gene prediction with a hidden Markov model and a new intron submodel. Bioinformatics **19**:215–225.

Stiven, A.E., and Gardner, S.A. (1992). Population processes in the ribbed mussel *Geukensia demissa* (Dillwyn) in a North Carolina salt marsh tidal gradient: spatial pattern, predation, growth and mortality. J. Exp. Mar. Biol. Ecol. **160**:81–102.

Strong, D.R., and Ayres, D.R. (2013). Ecological and evolutionary misadventures of *Spartina*. Annu. Rev. Ecol. Evol. Syst. **44**:389–410.

Tang, S., Lomsadze, A., and Borodovsky, M. (2015). Identification of protein coding regions in RNA transcripts. Nucleic Acids Res. **43**:e78.

Turner, S.D. (2014). qqman: an R package for visualizing GWAS results using Q–Q and manhattan plots. Preprint at bioRxiv. https://doi.org/10.1101/005165.

van Kleunen, M., Dawson, W., Essl, F., Pergl, J., Winter, M., Weber, E., Kreft, H., Weigelt, P., Kartesz, J., Nishino, M., et al. (2015). Global exchange and accumulation of non-native plants. Nature **525**:100–103.

Wang, K., Li, M., and Hakonarson, H. (2010). ANNOVAR: functional annotation of genetic variants from high-throughput sequencing data. Nucleic Acids Res. **38**:e164.

Wang, Q. (2007). The Dynamics of Plant Community Distribution of the Salt Marshes in the Yangtze River Estuary as Influenced by Spartina Alterniflora Invasions (Fudan University). PhD Thesis.

Wang, Q., Wang, C.H., Zhao, B., Ma, Z.J., Luo, Y.Q., Chen, J.K., and Li, B. (2006). Effects of growing conditions on the growth of and interactions between salt marsh plants: Implications for invasibility of habitats. Biol. Invasions **8**:1547–1560.

Wang, X.F., Zhang, Y.X., Niu, Y.Q., Sha, Y., Wang, Z.H., Zhang, Z.B., Yang, J., Liu, B., and Li, L.F. (2023). Post-hybridization introgression and natural selection promoted genomic divergence of *Aegilops speltoides* and the four S*-genome diploid species. Plant J. **115**:1500–1513.

Wang, X.Y., Shen, D.W., Jiao, J., XU, N.N., Yu, S., Zhou, X.F., Shi, M.M., and Chen, X.Y. (2012). Genotypic diversity enhances invasive ablity of *Spartina alterniflora*. Mol. Ecol. **21**:2542–2551.

Wang, Y., Wang, Y., Liu, X., and Tang, W. (2021). Research progress in photoperiodic control of flowering in rice. Chin. J. Rice Sci. **35**:207.

Wang, Z., Kang, M., Li, J., Zhang, Z., Wang, Y., Chen, C., Yang, Y., and Liu, J. (2022). Genomic evidence for homoploid hybrid speciation between ancestors of two different genera. Nat. Commun. **13**:1987.

Wu, S., Wang, Y., Wang, Z., Shrestha, N., and Liu, J. (2022). Species divergence with gene flow and hybrid speciation on the Qinghai–Tibet Plateau. New Phytol. **234**:392–404.

van der Weijden, W., Leewis, R., and Bol, P. (2007). 100 of the world's worst invasive alien species. Biological Globalisation, 206–208.

Wu, T., Hu, E., Xu, S., Chen, M., Guo, P., Dai, Z., Feng, T., Zhou, L., Tang, W., Zhan, L., et al. (2021). clusterProfiler 4.0: A universal enrichment tool for interpreting omics data. Innov. J. **2**, 100141.

Wu, W., Zheng, X.M., Lu, G., Zhong, Z., Gao, H., Chen, L., Wu, C., Wang, H.J., Wang, Q., Zhou, K., et al. (2013). Association of functional nucleotide polymorphisms at *DTH2* with the northward expansion of rice cultivation in Asia. Proc. Natl. Acad. Sci. USA **110**:2775–2780.

Xia, L., Geng, Q., and An, S. (2020). Rapid genetic divergence of an invasive species, *Spartina alterniflora*, in China. Front. Genet. **11**:284.

Xu, G.W., and Zhuo, R.Z. (1985). Preliminary studies of introduced *Spartina alterniflora* Loisel in China. J. Nanjing For. Univ. (Nat. Sci. Ed.) **40**:212–225.

Xu, Z., and Wang, H. (2007). LTR_FINDER: an efficient tool for the prediction of full-length LTR retrotransposons. Nucleic Acids Res. **35**:W265–W268.

Zenetos, A., Gofas, S., Verlaque, M., Çinar, M.E., García Raso, J.E., Bianchi, C., Morri, C., Azzurro, E., Bilecenoglu, M., Froglia, C., et al. (2010). Alien species in the Mediterranean Sea by 2010. A contribution to the application of European Union's Marine Strategy Framework Directive (MSFD). Part I. Spatial distribution. Mediterr. Mar. Sci. **11**:381–493.

Zerebecki, R.A., Sotka, E.E., Hanley, T.C., Bell, K.L., Gehring, C., Nice, C.C., Richards, C.L., and Hughes, A.R. (2021). Repeated genetic and adaptive phenotypic divergence across tidal elevation in a foundation plant species. Am. Nat. **198**:E152–E169.

Zhang, C., Dong, S.S., Xu, J.Y., He, W.M., and Yang, T.L. (2019). PopLDdecay: a fast and effective tool for linkage disequilibrium decay analysis based on variant call format files. Bioinformatics **35**:1786–1788.

Zhang, J., Zhou, X., Yan, W., Zhang, Z., Lu, L., Han, Z., Zhao, H., Liu, H., Song, P., Hu, Y., et al. (2015). Combinations of the *Ghd7, Ghd8* and *Hd1* genes largely define the ecogeographical adaptation and yield potential of cultivated rice. New Phytol. **208**:1056–1066.

Zhang, R., Shen, Y., Lu, L., Yan, S., Wang, Y., Li, J., and Zhang, Z. (2004). Formation of *Spartina alterniflora* salt marshes on the coast of Jiangsu Province, China. Ecol. Eng. **23**:95–105.

Zhou, S., Zhu, S., Cui, S., Hou, H., Wu, H., Hao, B., Cai, L., Xu, Z., Liu, L., Jiang, L., et al. (2021). Transcriptional and post-transcriptional regulation of heading date in rice. New Phytol. **230**:943–956.

 © 2024 The Author(s).

Plant Communications, Volume *5*

Supplemental information

Genomic and phenotypic signatures provide insights into the wide adaptation of a global plant invader

Yan Hao, Xin-Feng Wang, Yaolin Guo, Tian-Yang Li, Ji Yang, Malika L. Ainouche, Armel Salmon, Rui-Ting Ju, Ji-Hua Wu, Lin-Feng Li, and Bo Li

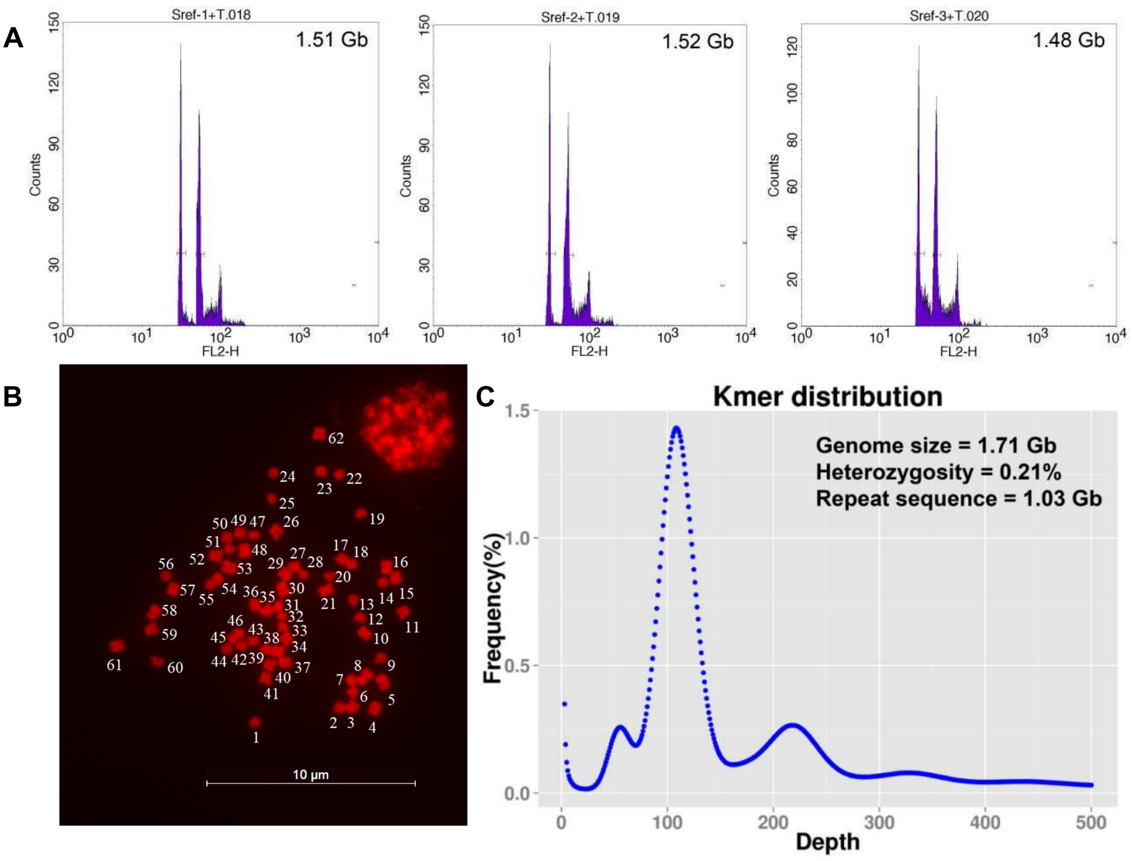

Supplementary Figure 1. (**A**) Genome size of *S. alterniflora* estimated by flow cytometry. Samples from left (1.51 Gb), middle (1.52 Gb) to right (1.48 Gb) are three biological replicates. (**B**) Karyotype of the US sample used to assemble the reference genome. A total of 62 chromosomes were identified. (**C**) K-mer (k = 19) analysis of the *S. alterniflora* genome based on Illumina short reads. A total of 218.01 Gb short reads were used to perform K-mer analysis. The heterozygosity rate is 0.21%.

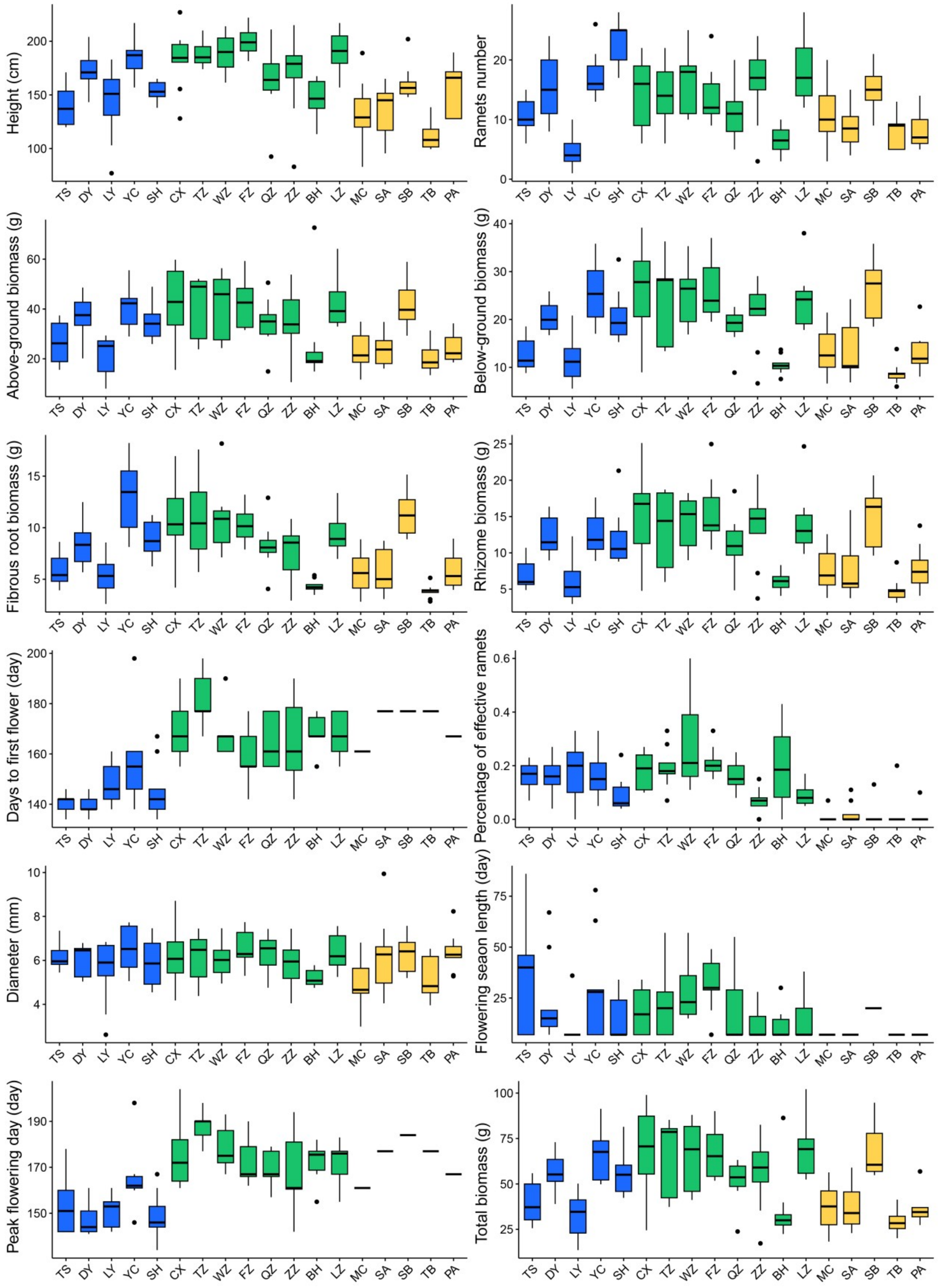

Supplementary Figure 2. Vegetative and reproductive traits of invasive Chinese and native US populations. All plants were planted in plastic pots (16 cm in diameter and 17.5 cm in height) containing a mixture of vermiculite and soil (1:3). All these selected seedlings were grown in greenhouse with 10‰ salinity concentration.

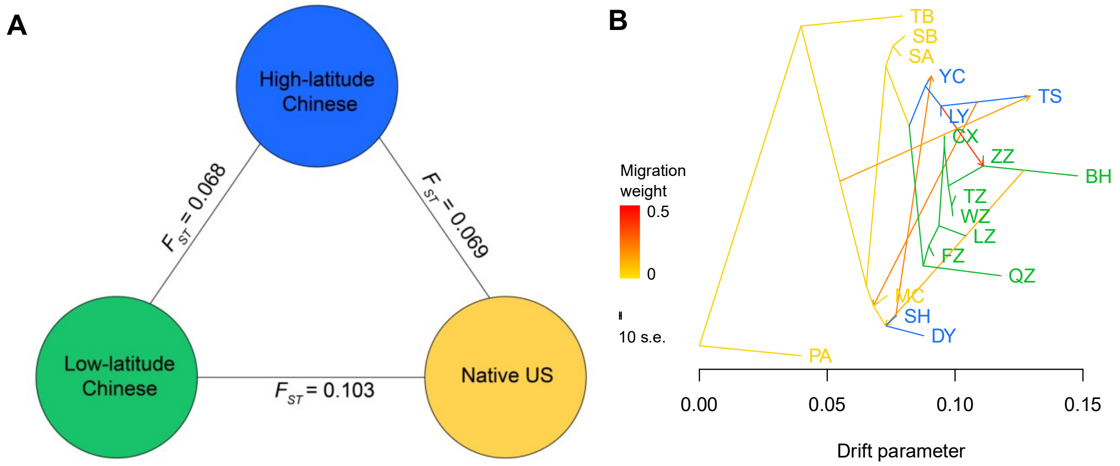

Supplementary Figure 3. (**A**) Genetic differentiation (F_{ST}) at the whole genome level among native US, high- and low-latitude Chinese populations. (**B**) Population split and mixture events inferred based on whole genome SNP dataset. Yellow, blue and green colors represent native US, high-latitude Chinese and low-latitude Chinese populations, respectively. Colored arrows indicate gene flow events among these native and invasive smooth cordgrass populations. Colors from orange to red indicate migration weight from weak to strong.

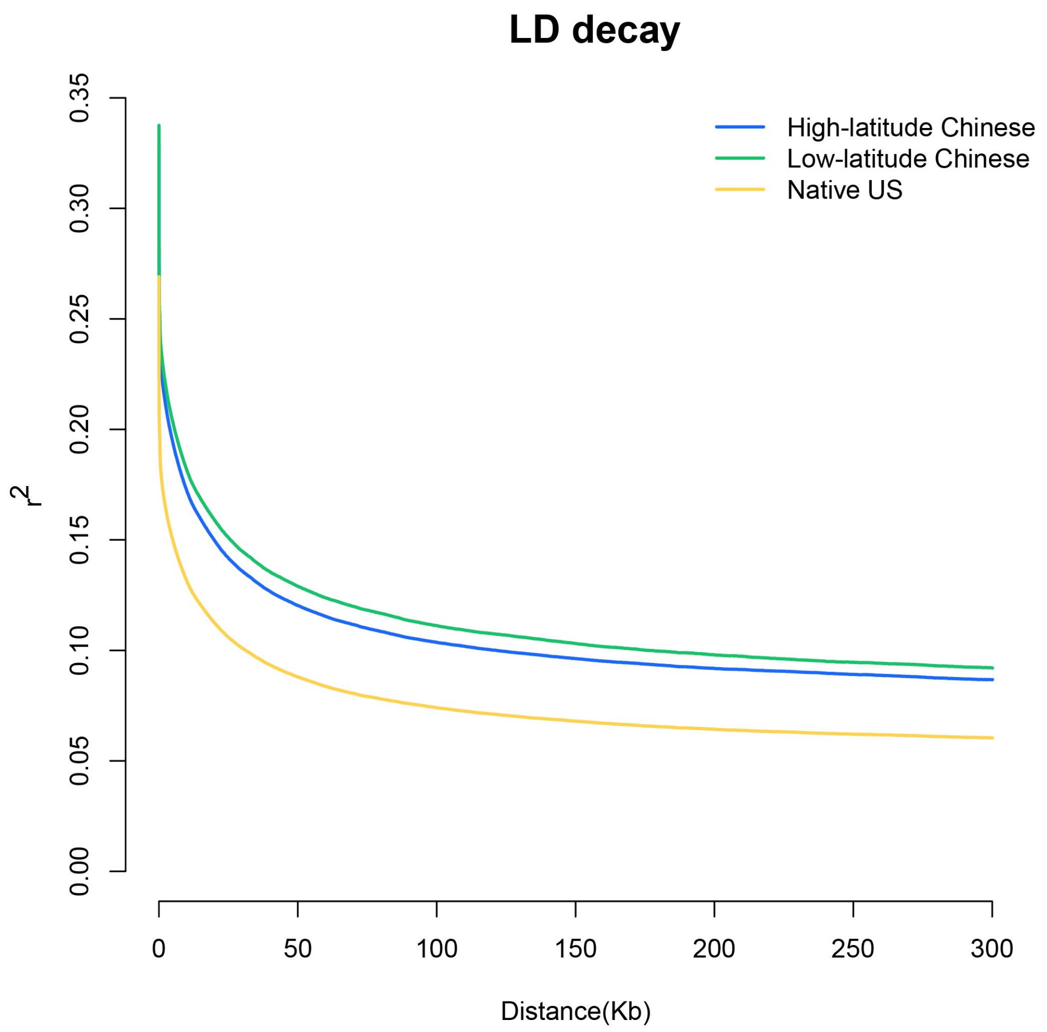

Supplementary Figure 4. Linkage disequilibrium decay of the native US, high- and low-latitude Chinese populations based on whole genome SNP dataset.

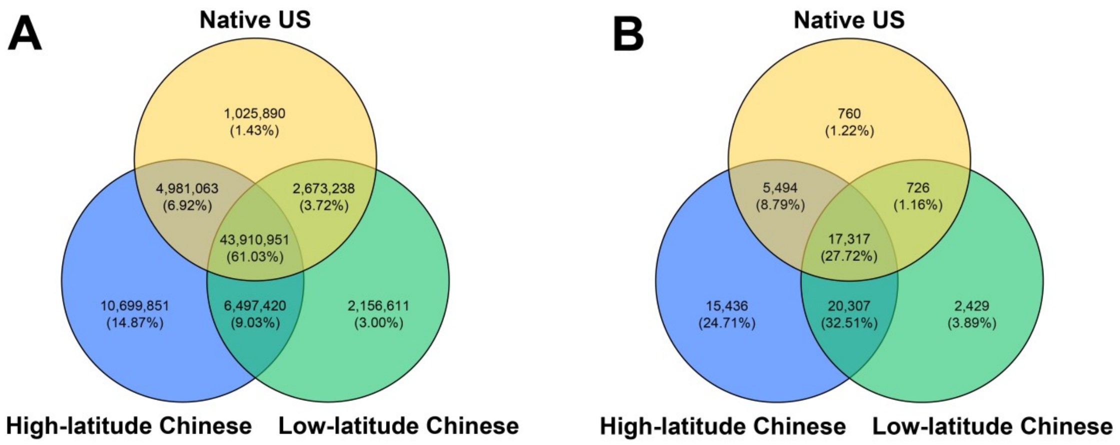

Supplementary Figure 5. Venn analysis of the specific- and shared-SNPs (**A**) and intersecting genes (**B**) among native US, low- and high-latitude Chinese populations. The intersecting genes are those that contain SNPs at gene body region.

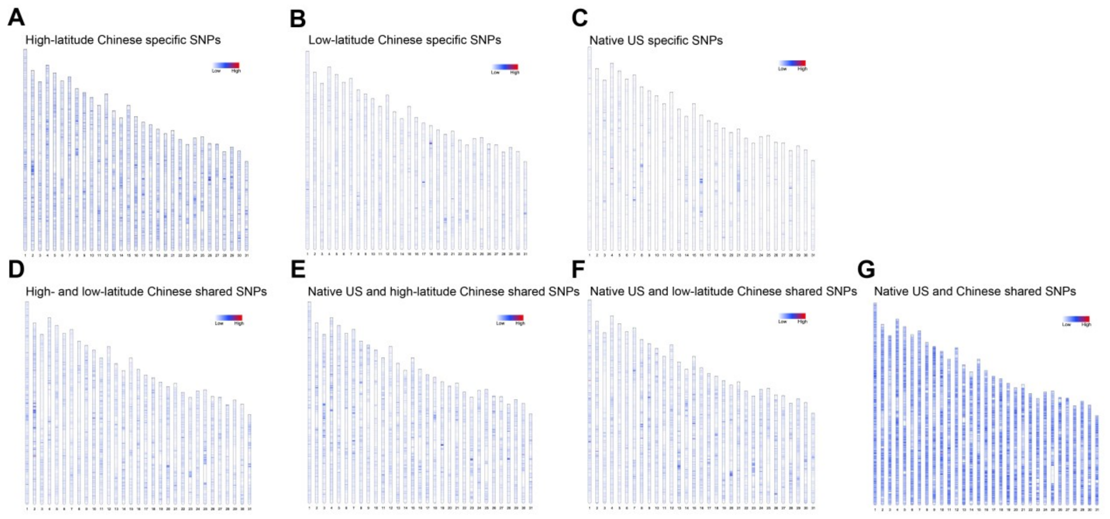

Supplementary Figure 6. Distribution patterns of the specific- and shared-SNPs on the 31 chromosomes. Colors from white to blue and red represent the numbers of SNPs for each 50-Kb sliding window. From subpanels (**A**) to (**G**) are the seven types of SNPs identified in Supplementary Figure 5A.

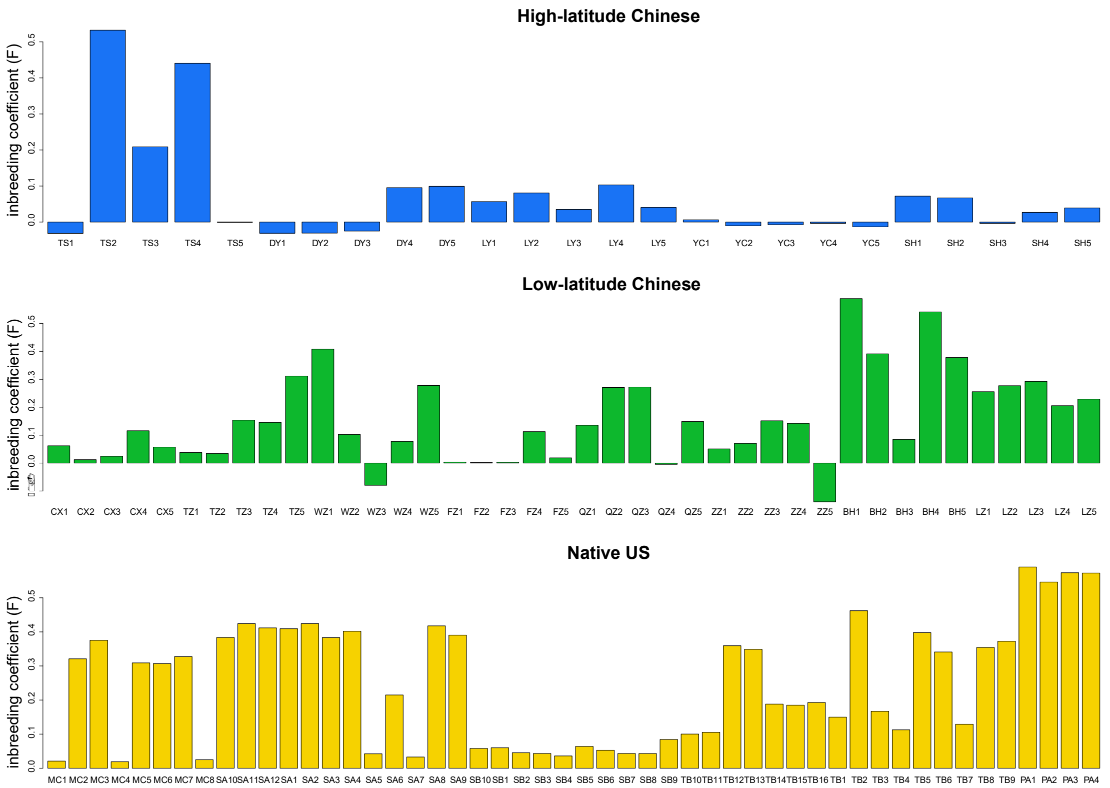

Supplementary Figure 7. Inbreeding coefficient of all native US and invasive Chinese accessions based on whole genome SNP dataset. Each bar on x axes represents an accession. Values on y axes are Inbreeding coefficient (Fis) for each accession.

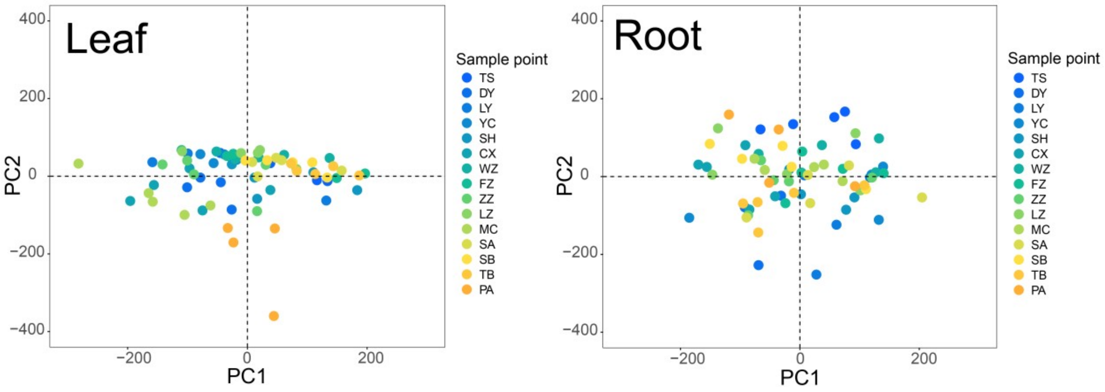

Supplementary Figure 8. Principal component analysis of the overall genes in leaf (on left) and root (on right) tissues, respectively. Different colors represent US and Chinese populations.

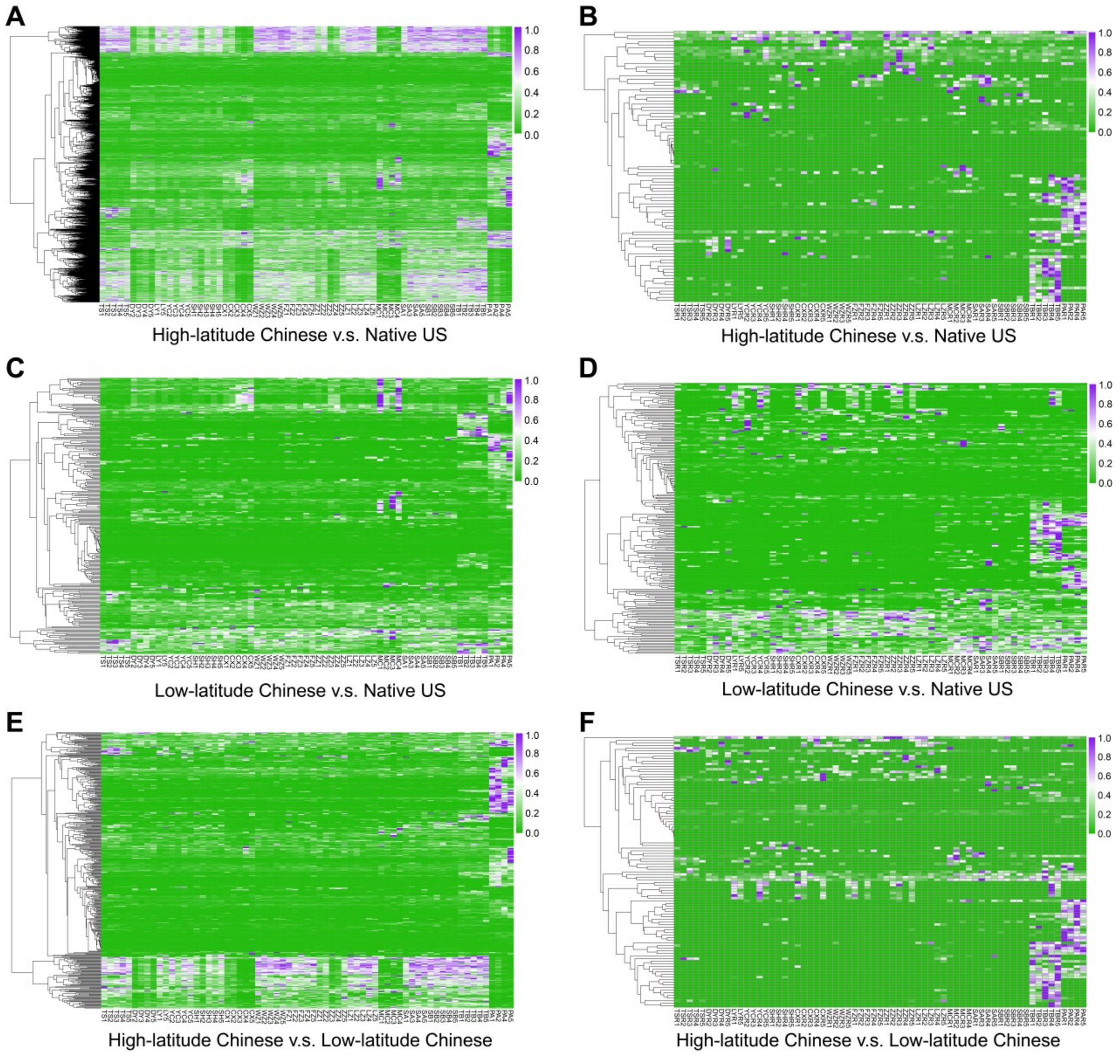

Supplementary Figure 9. Heatmap of differentially expressed genes (DEGs) identified between all invasive Chinese and native US accessions in leaf and root tissues, respectively. (**A**-**B**) Expression pattern of the DEGs identified between native US and high-latitude Chinese populations in leaf and root tissues, respectively. (**C**-**D**) Expression pattern of the DEGs identified between native US and low-latitude Chinese populations in leaf and root tissues, respectively. (**E**-**F**) Expression pattern of the DEGs identified between low- and high-latitude Chinese populations in leaf and root tissues, respectively.

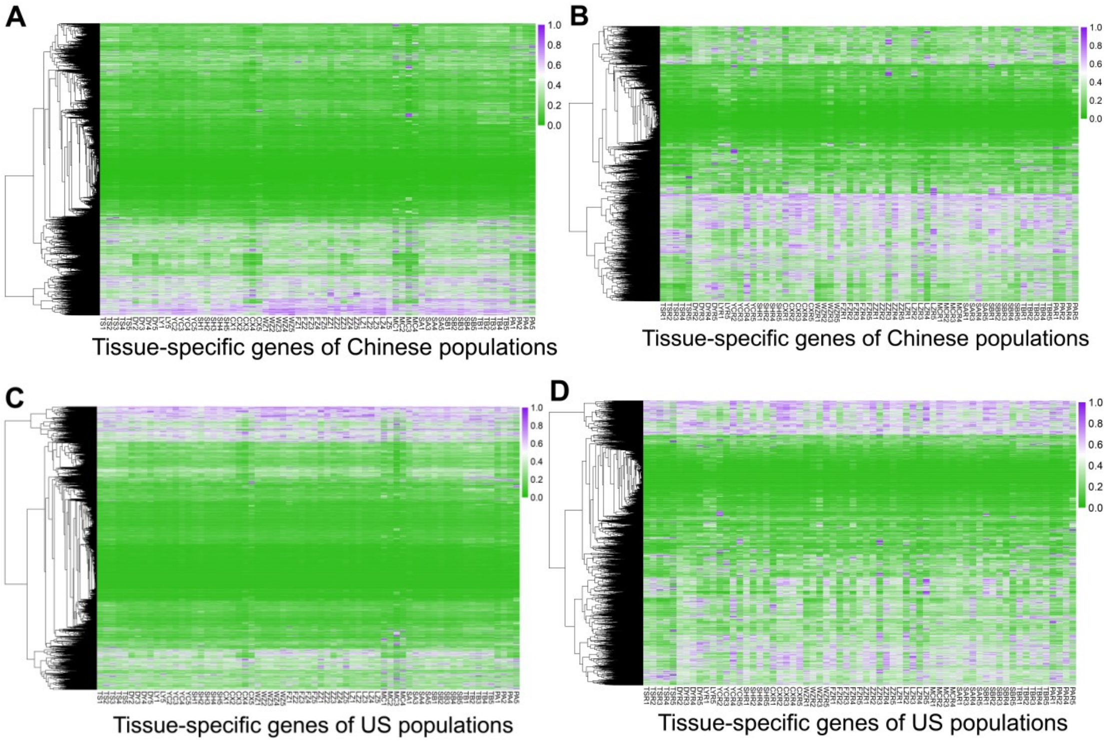

Supplementary Figure 10. Expression pattern of the tissue-specific genes identified between root and leaf tissues. (**A**-**B**) Tissue-specific genes identified in root and leaf tissues of Chinese populations, respectively. (**C**-**D**) Tissue-specific genes identified in root and leaf tissues of US populations, respectively.

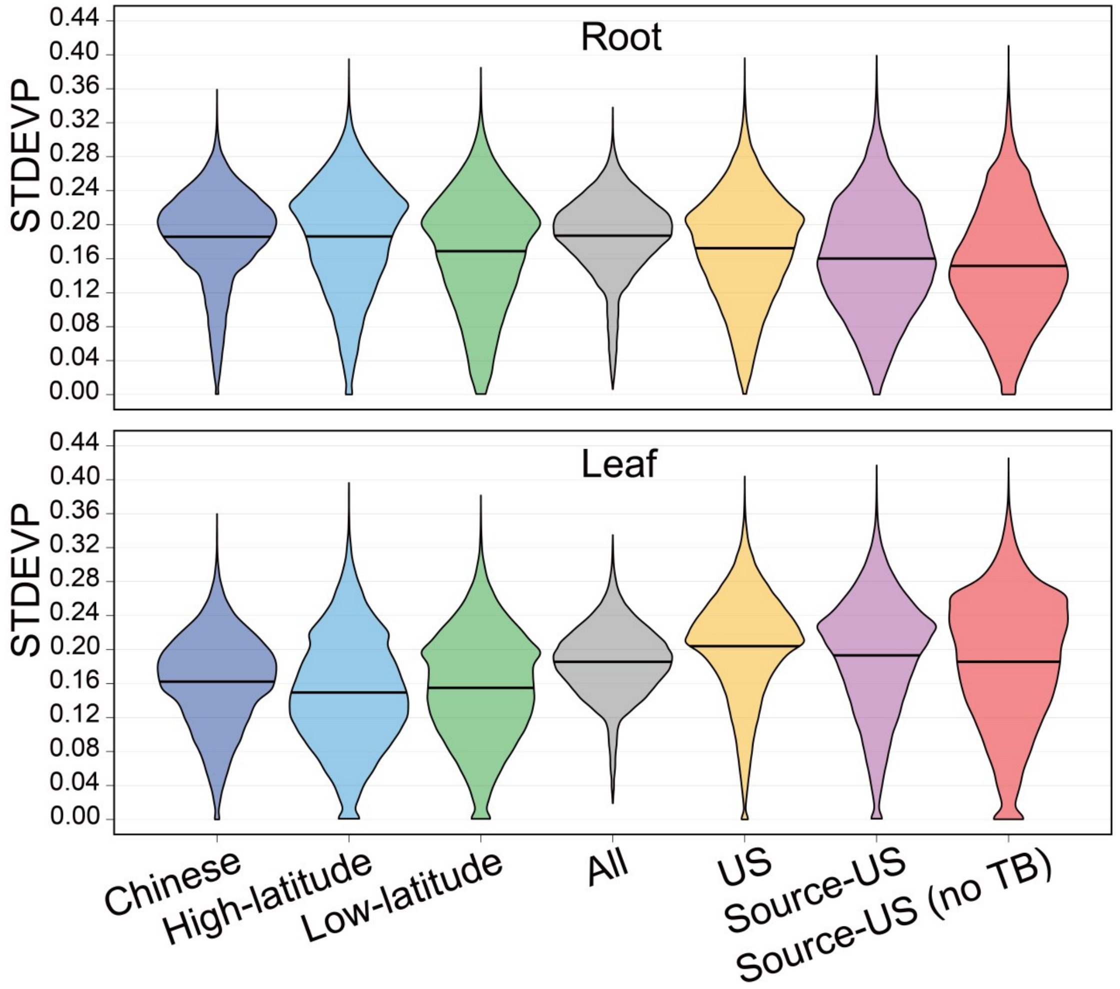

Supplementary Figure 11. Gene expression divergence in leaf and root tissues of the US and Chinese groups. Each colored violin represents a group of the US and Chinese populations. The black line in the violin is the mean value. Values on y axes are standard deviation (STDEVP) for each group.

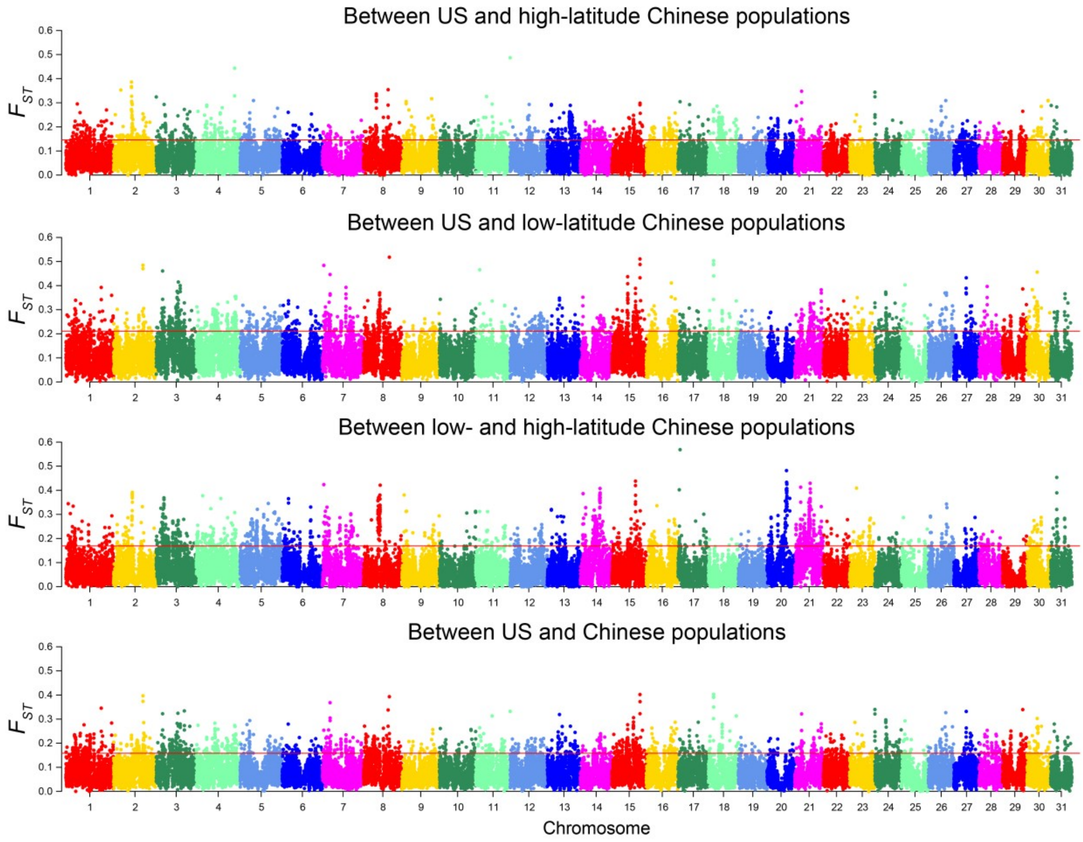

Supplementary Figure 12. Genetic differentiation (F_{ST}) among native US, low- and high-latitude populations of *S. alterniflora*. Each dot represents a 50-Kb genomic region. Dots above the red line is the top 5% highest divergent genomic regions.

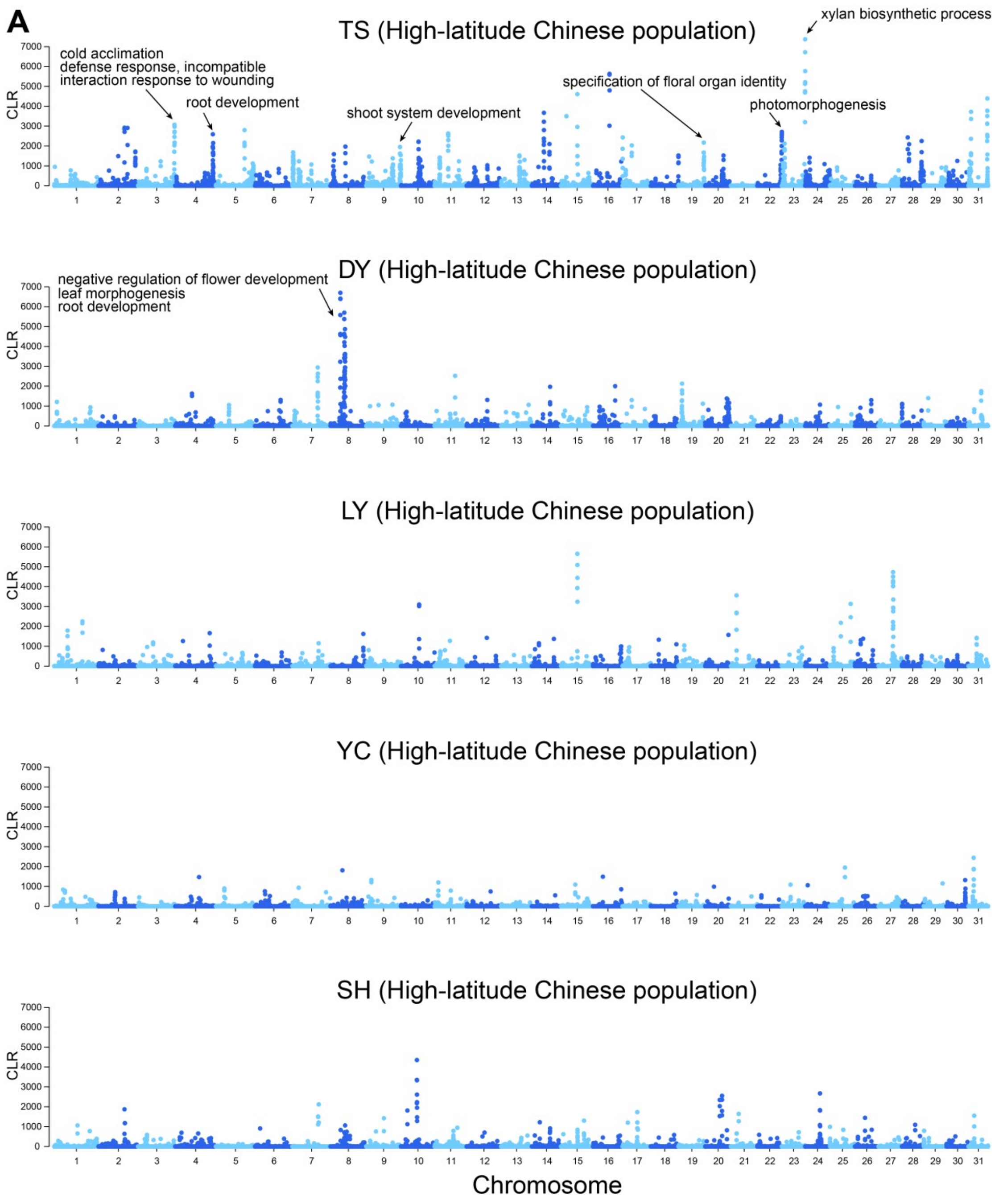
A
TS (High-latitude Chinese population)
cold acclimation
defense response, incompatible
interaction response to wounding
root development
shoot system development
specification of floral organ identity
photomorphogenesis
xylan biosynthetic process
DY (High-latitude Chinese population)
negative regulation of flower development
leaf morphogenesis
root development
LY (High-latitude Chinese population)
YC (High-latitude Chinese population)
SH (High-latitude Chinese population)
CLR
Chromosome

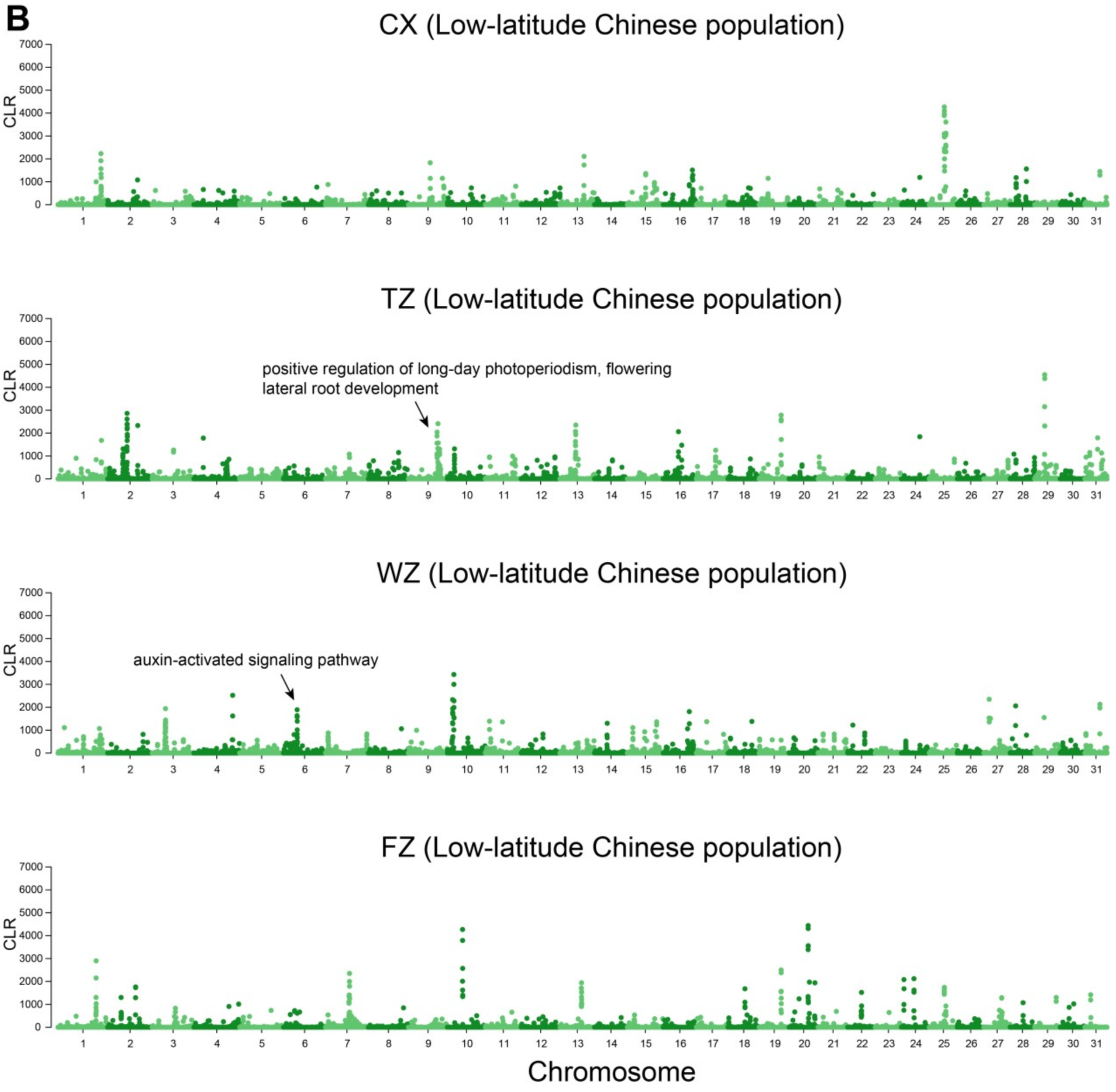
B
CX (Low-latitude Chinese population)
TZ (Low-latitude Chinese population)
positive regulation of long-day photoperiodism, flowering
lateral root development
WZ (Low-latitude Chinese population)
auxin-activated signaling pathway
FZ (Low-latitude Chinese population)
CLR
Chromosome

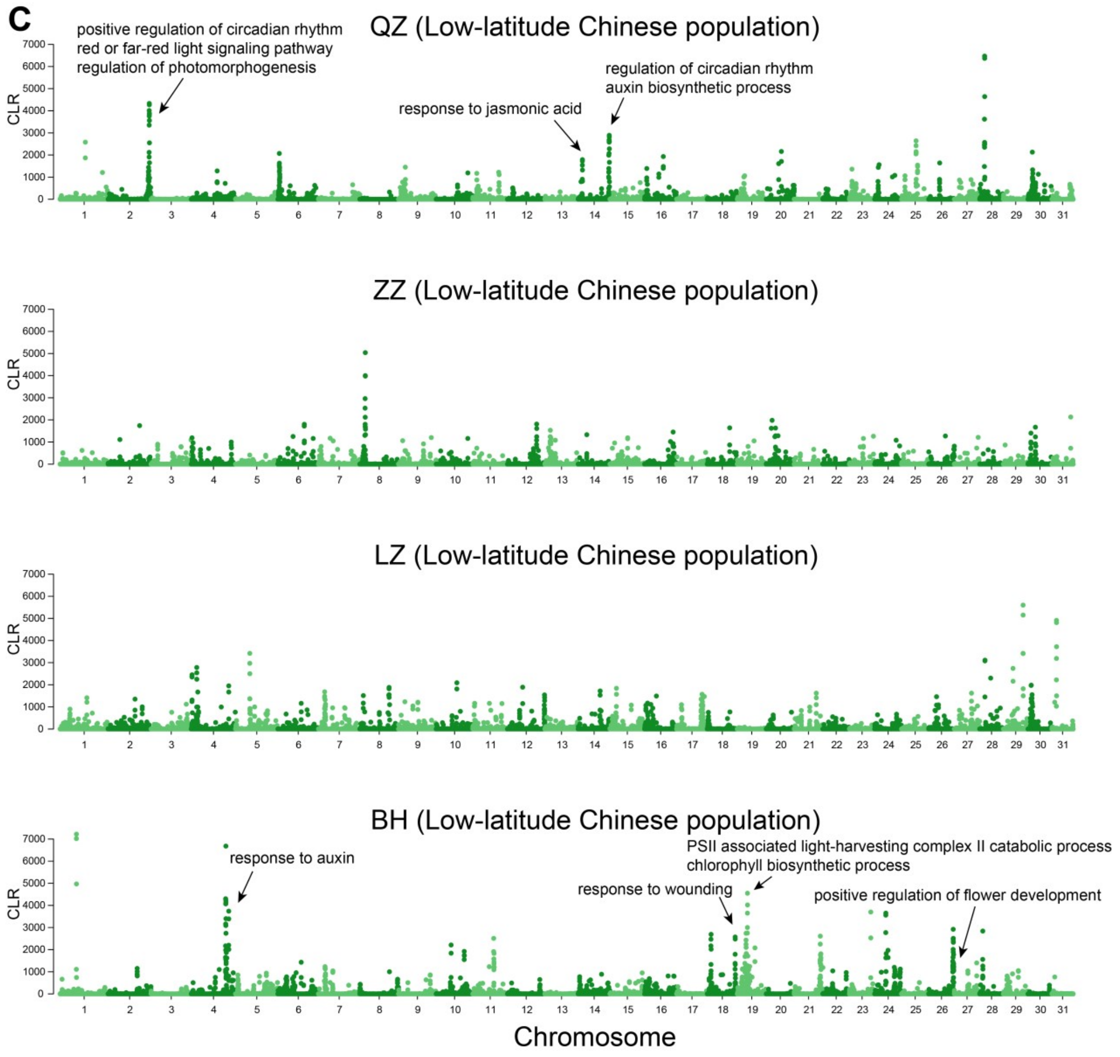
C
QZ (Low-latitude Chinese population)
positive regulation of circadian rhythm
red or far-red light signaling pathway
regulation of photomorphogenesis
response to jasmonic acid
regulation of circadian rhythm
auxin biosynthetic process
ZZ (Low-latitude Chinese population)
LZ (Low-latitude Chinese population)
BH (Low-latitude Chinese population)
response to auxin
PSII associated light-harvesting complex II catabolic process
chlorophyll biosynthetic process
response to wounding
positive regulation of flower development
CLR
Chromosome

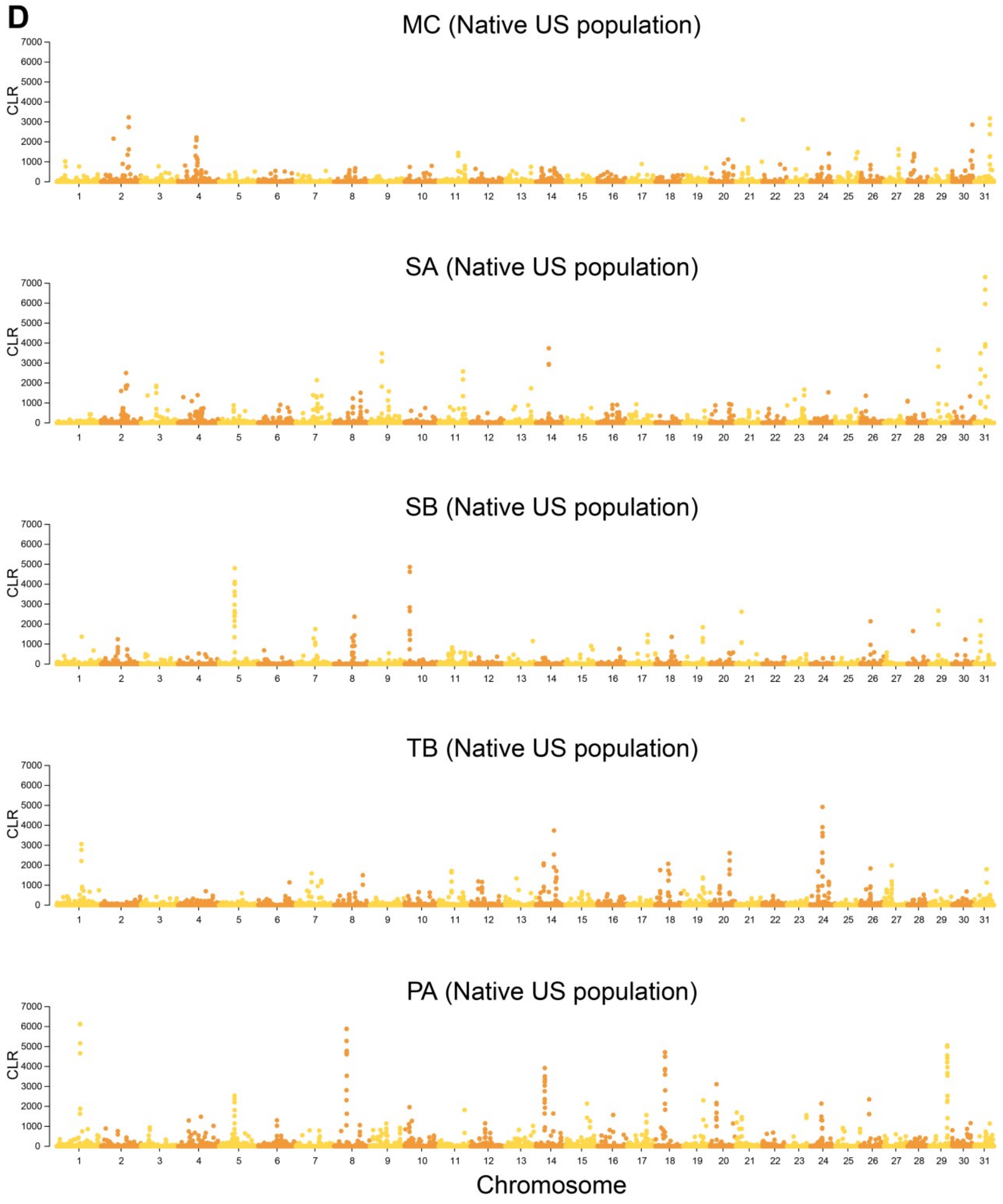

Supplementary Figure 13. Selective genomic regions along the chromosomes of *S. alterniflora* that identified by SweeD in high-latitude Chinese (**A**), low-latitude Chinese (**B**-**C**) and native US populations (**D**). Values on y axes represent the composite likelihood ratio (CLR). Each dot is a 50-Kb genomic region on the chromosome. Population names are the same as Supplementary Table S16.

BIO1: Annual Mean Temperature

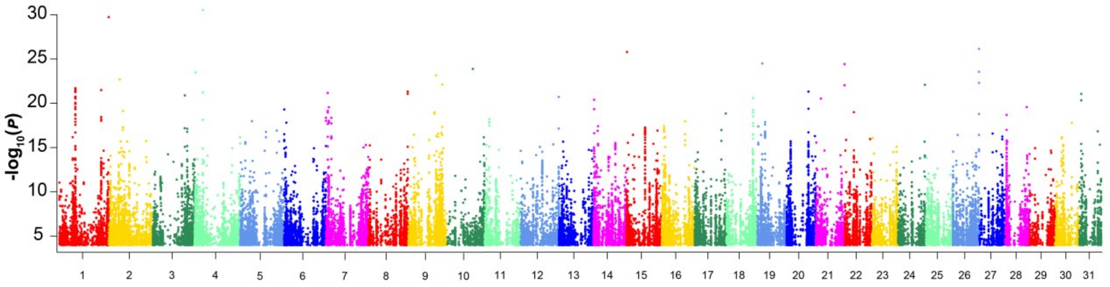

BIO2: Mean Diurnal Range (Mean of monthly (max temp - min temp))

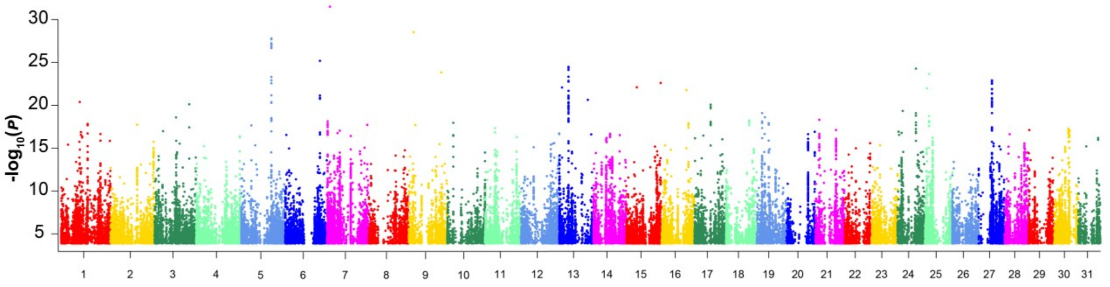

BIO3: Isothermality

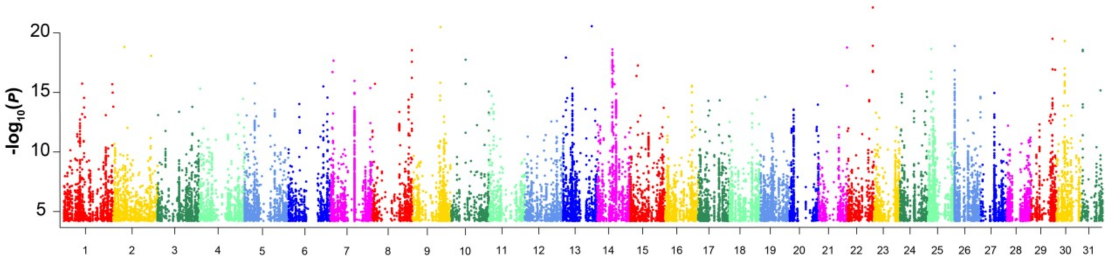

BIO4: Temperature Seasonality (standard deviation ×100)

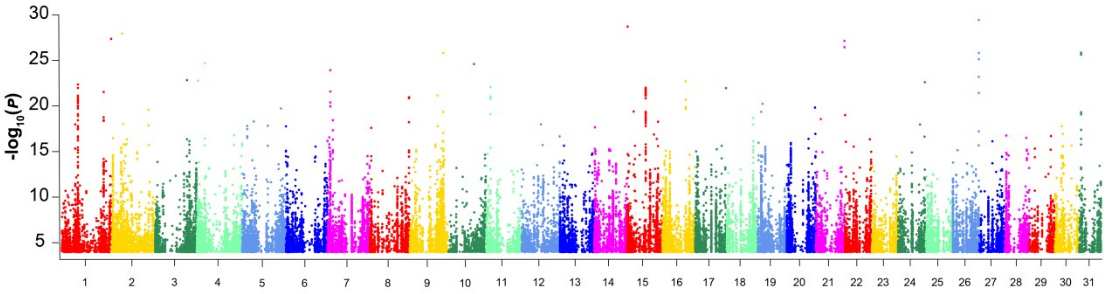

BIO5: Max Temperature of Warmest Month

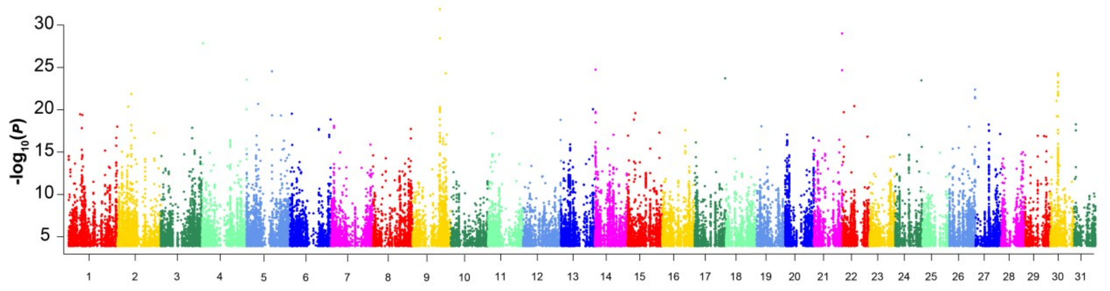

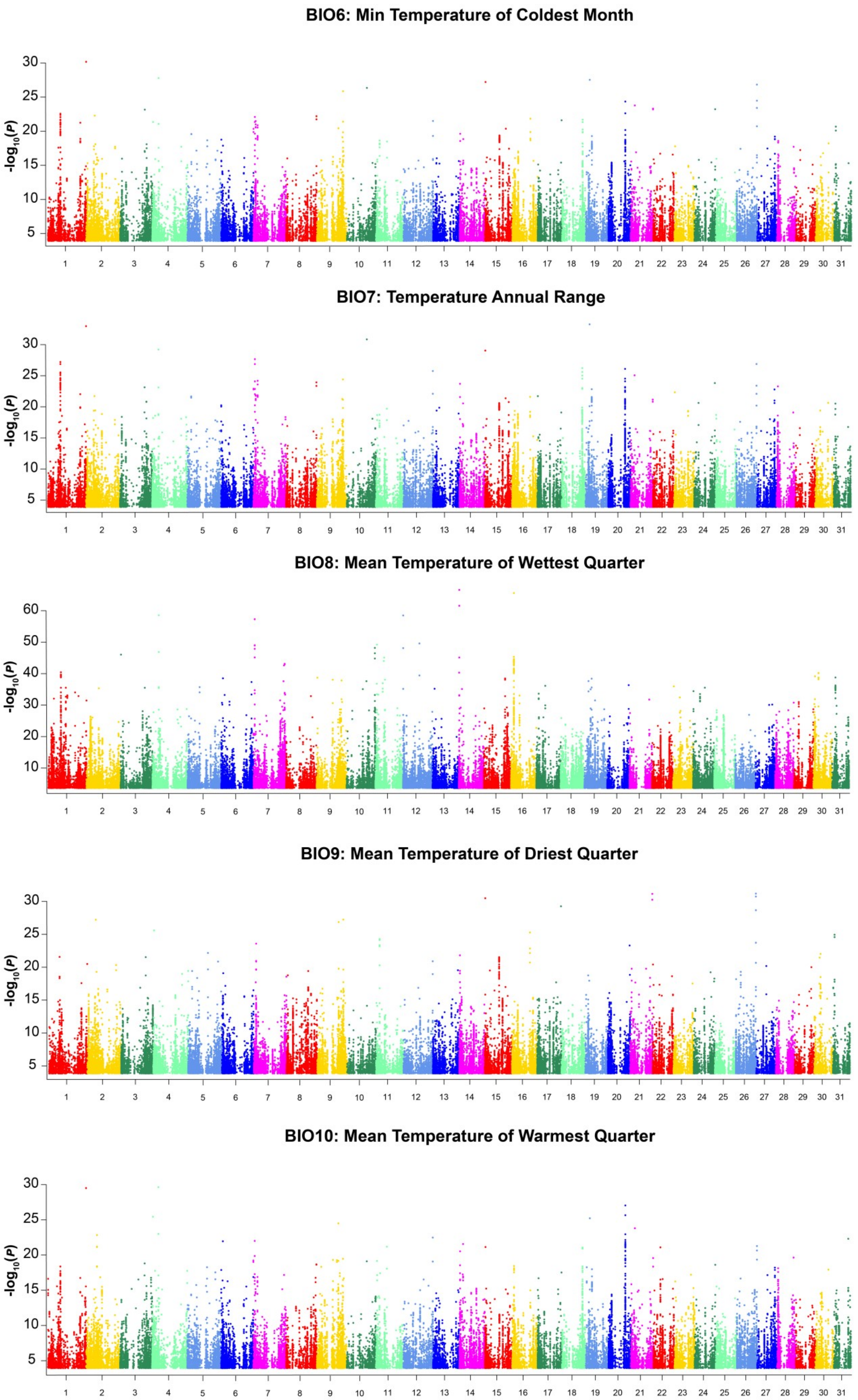
BIO6: Min Temperature of Coldest Month
BIO7: Temperature Annual Range
BIO8: Mean Temperature of Wettest Quarter
BIO9: Mean Temperature of Driest Quarter
BIO10: Mean Temperature of Warmest Quarter
$-\log_{10}(P)$
1 2 3 4 5 6 7 8 9 10 11 12 13 14 15 16 17 18 19 20 21 22 23 24 25 26 27 28 29 30 31

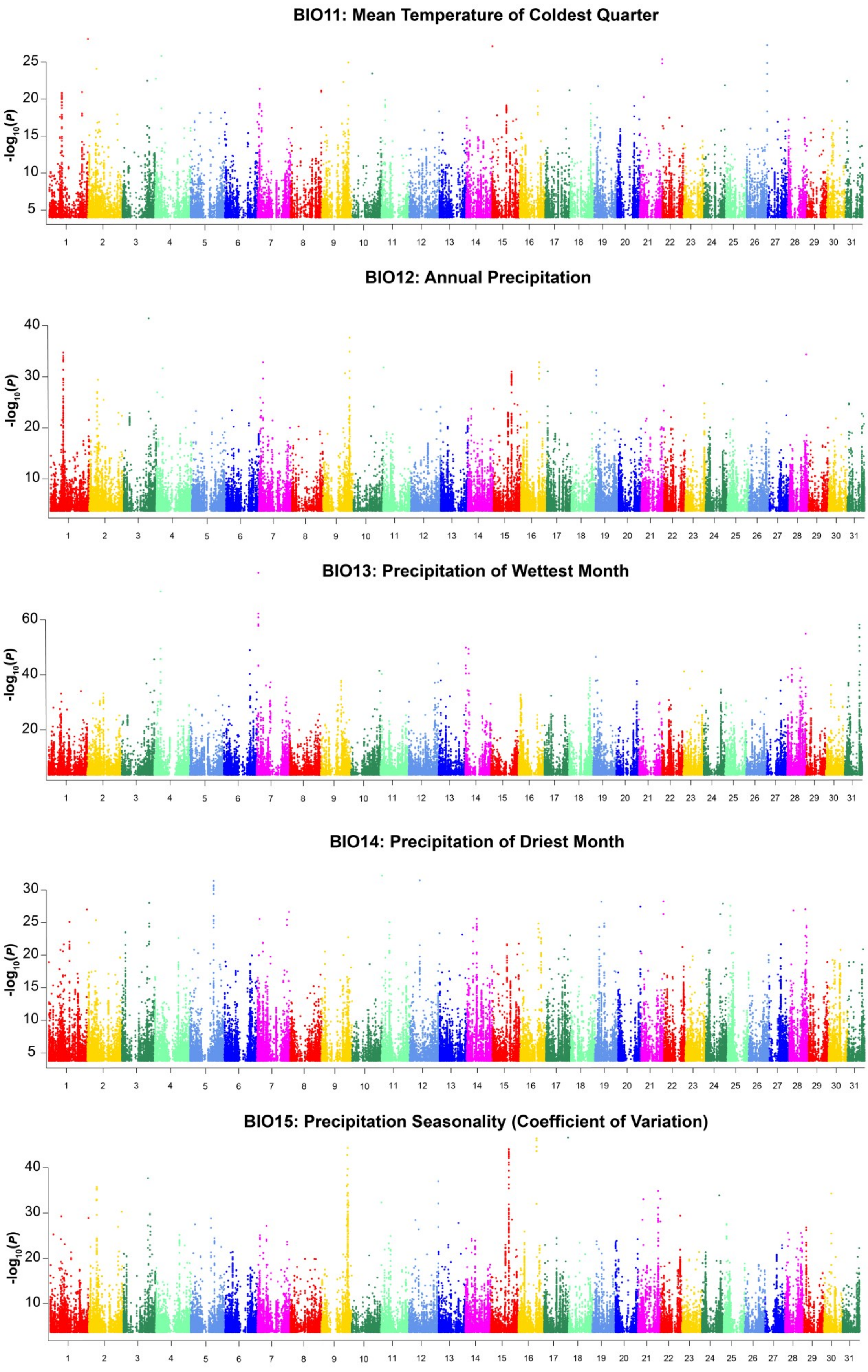
BIO11: Mean Temperature of Coldest Quarter
BIO12: Annual Precipitation
BIO13: Precipitation of Wettest Month
BIO14: Precipitation of Driest Month
BIO15: Precipitation Seasonality (Coefficient of Variation)
$-\log_{10}(P)$
1 2 3 4 5 6 7 8 9 10 11 12 13 14 15 16 17 18 19 20 21 22 23 24 25 26 27 28 29 30 31

BIO16: Precipitation of Wettest Quarter

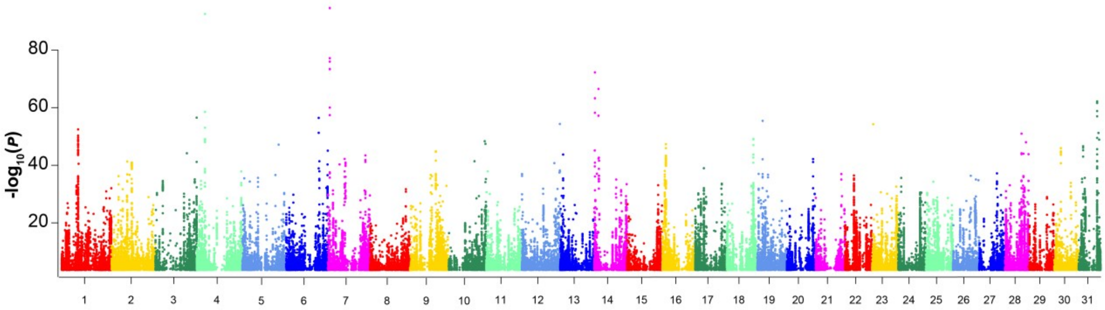

BIO17: Precipitation of Driest Quarter

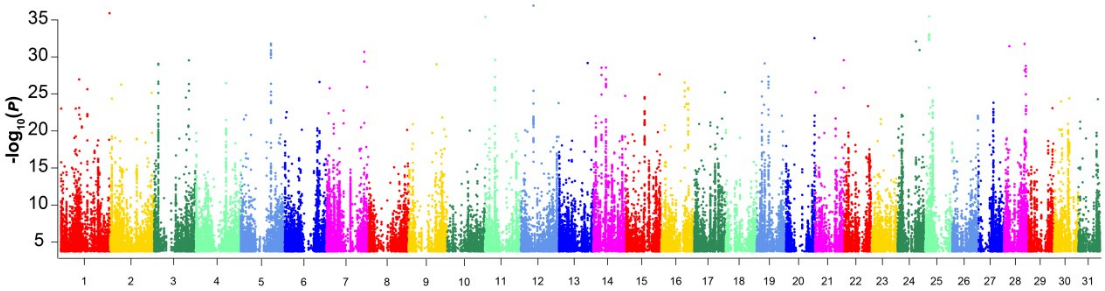

BIO18: Precipitation of Warmest Quarter

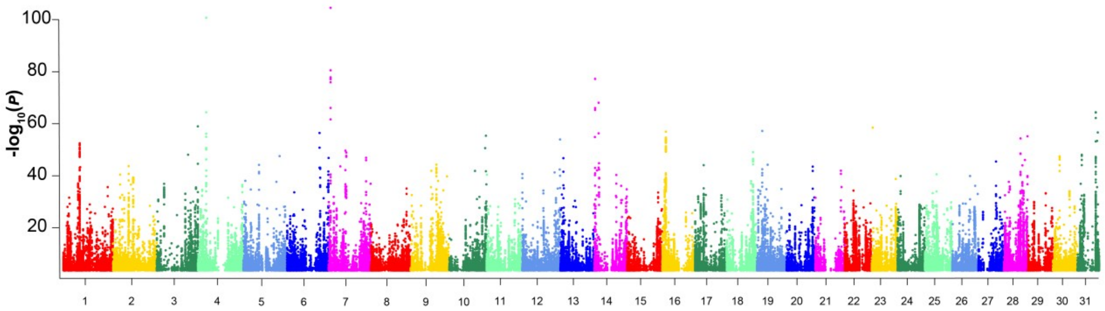

BIO19: Precipitation of Coldest Quarter

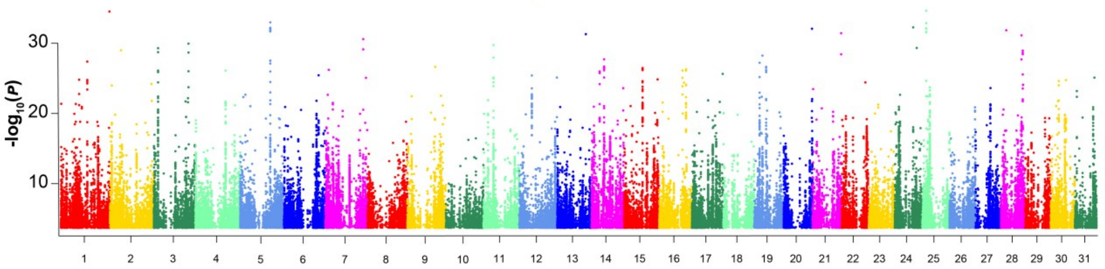

BIO20: Actual Evapotranspiration

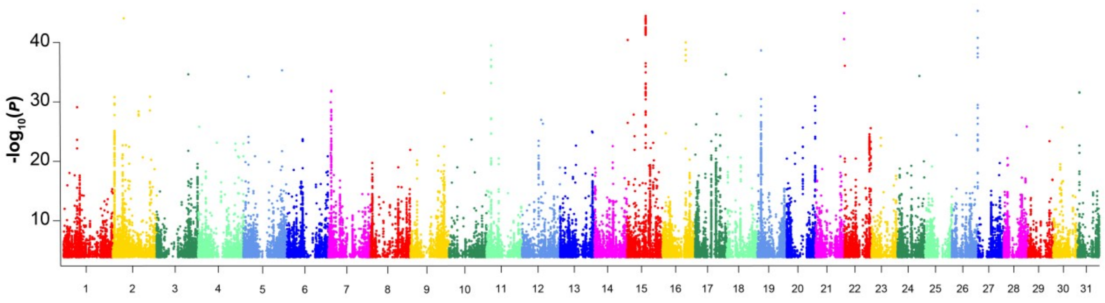

BIO21: Climate Water Deficit

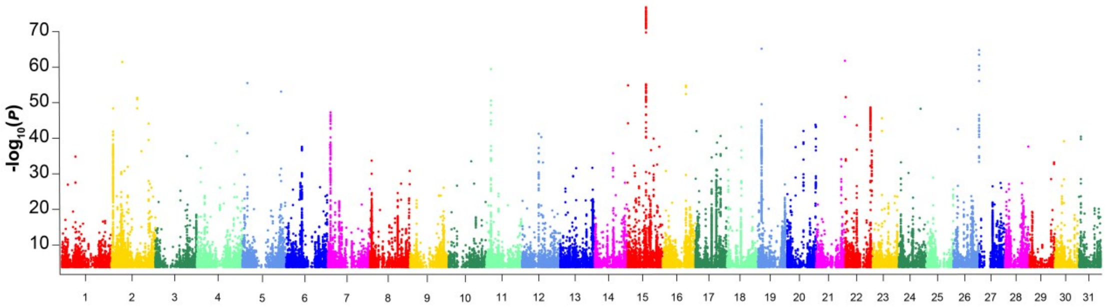

BIO22: Palmer Drought Severity Index

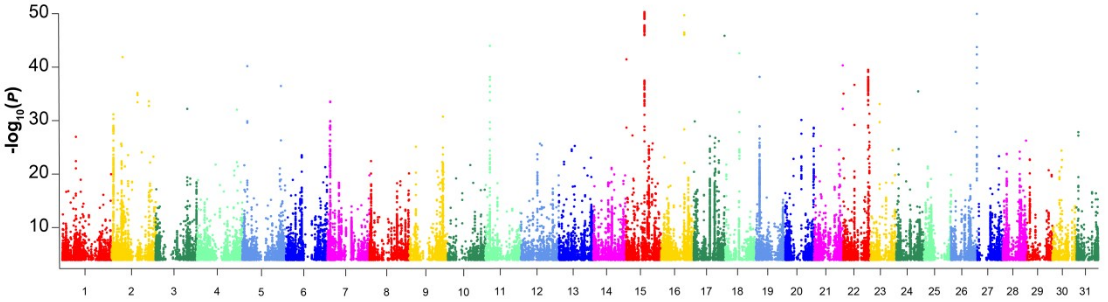

BIO23: Precipitation Accumulation

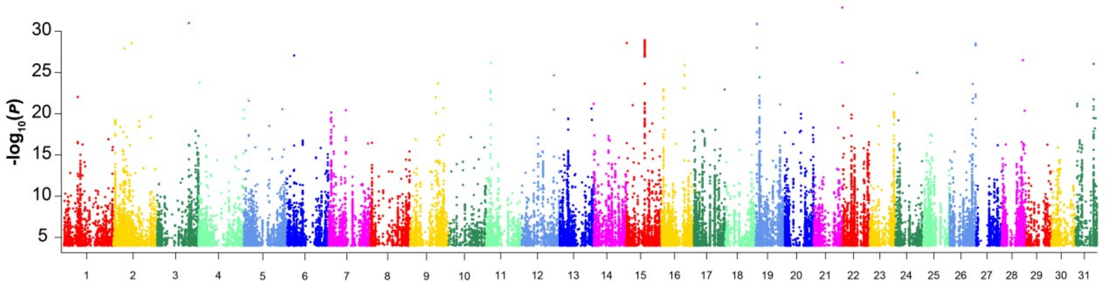

BIO24: Soil Moisture

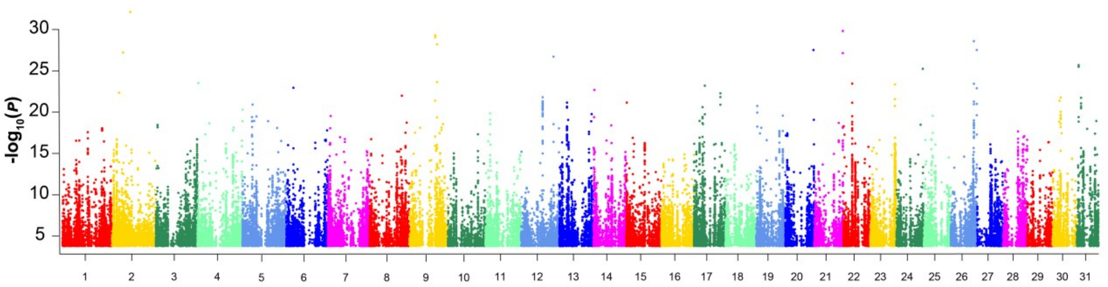

BIO25: Downward Surface Shortwave Radiation

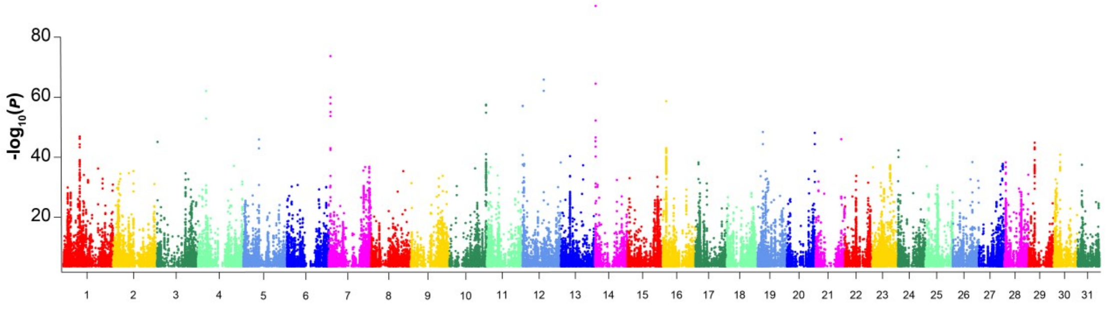

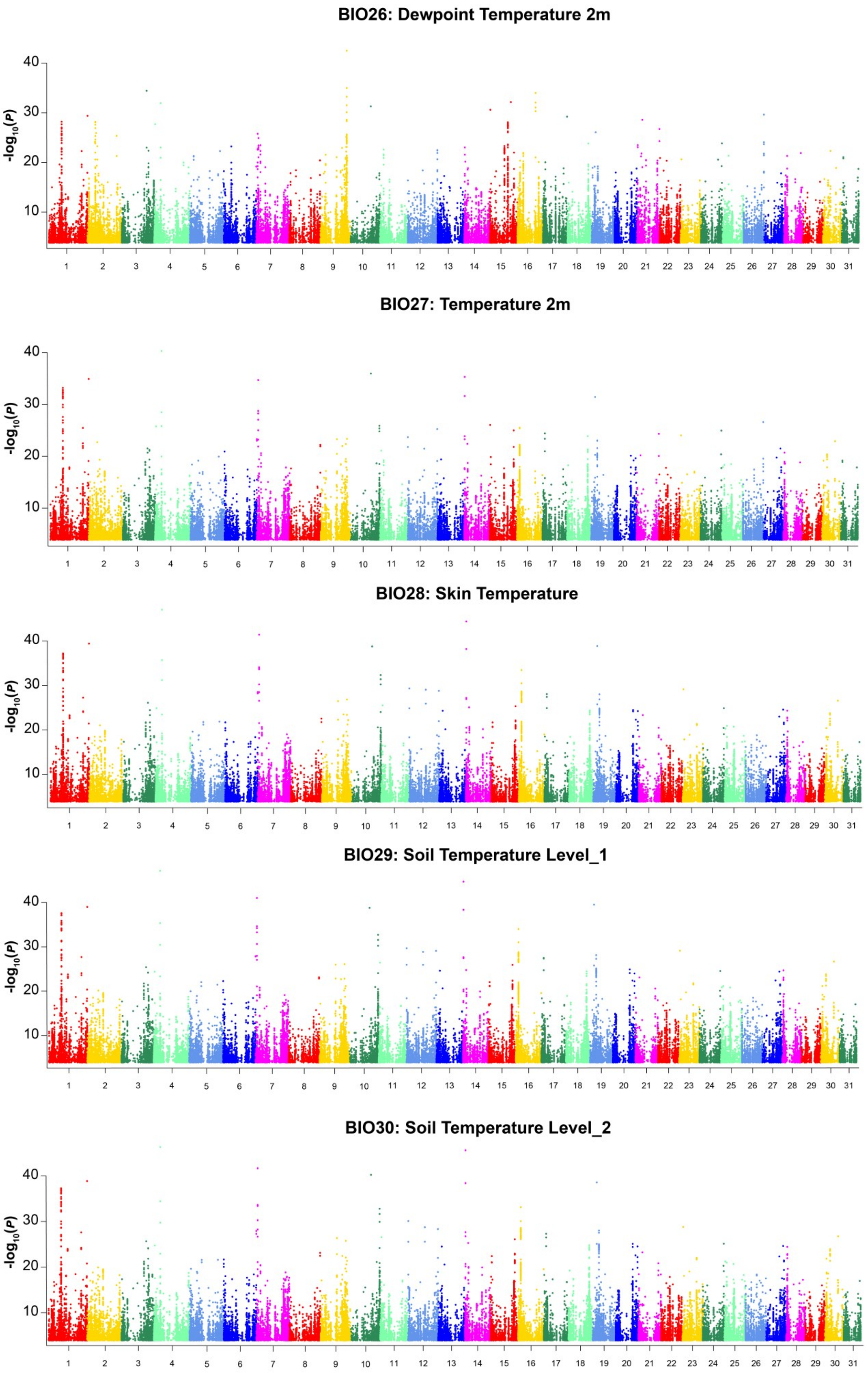
BIO26: Dewpoint Temperature 2m
BIO27: Temperature 2m
BIO28: Skin Temperature
BIO29: Soil Temperature Level_1
BIO30: Soil Temperature Level_2
$-\log_{10}(P)$
10
20
30
40
1 2 3 4 5 6 7 8 9 10 11 12 13 14 15 16 17 18 19 20 21 22 23 24 25 26 27 28 29 30 31

BIO31: Volumetric Soil Water Layer_1

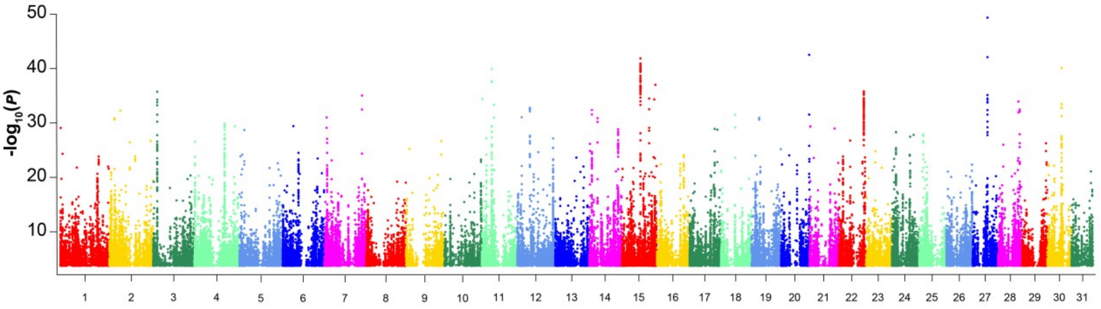

BIO32: Volumetric Soil Water Layer_2

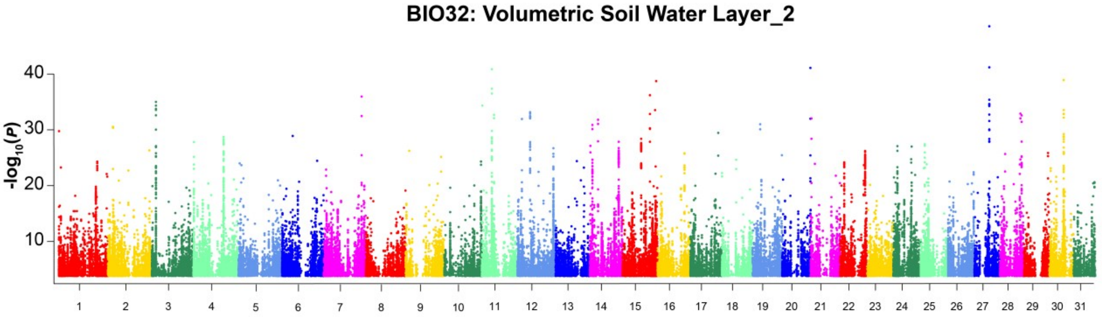

BIO33: Surface Latent Heat Flux

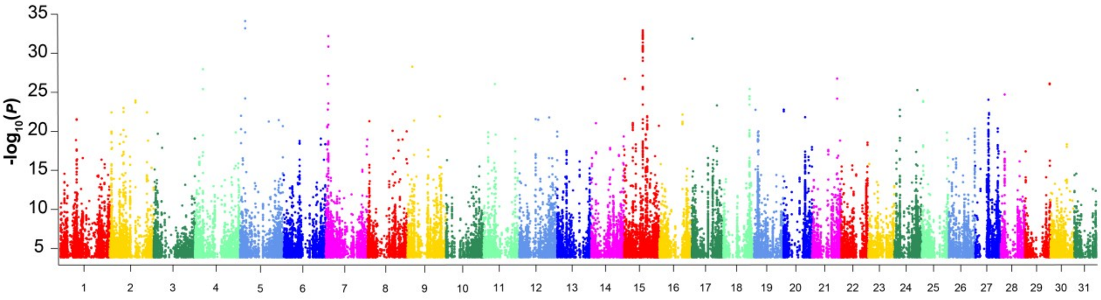

BIO34: Surface Net Solar Radiation

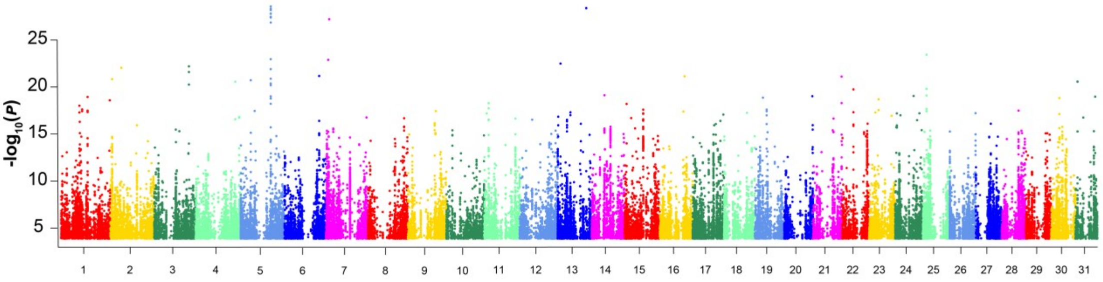

BIO35: Surface Net Thermal Radiation

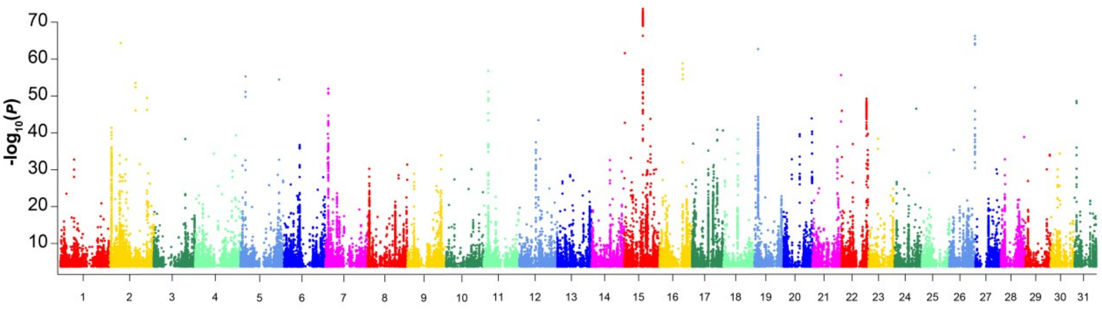

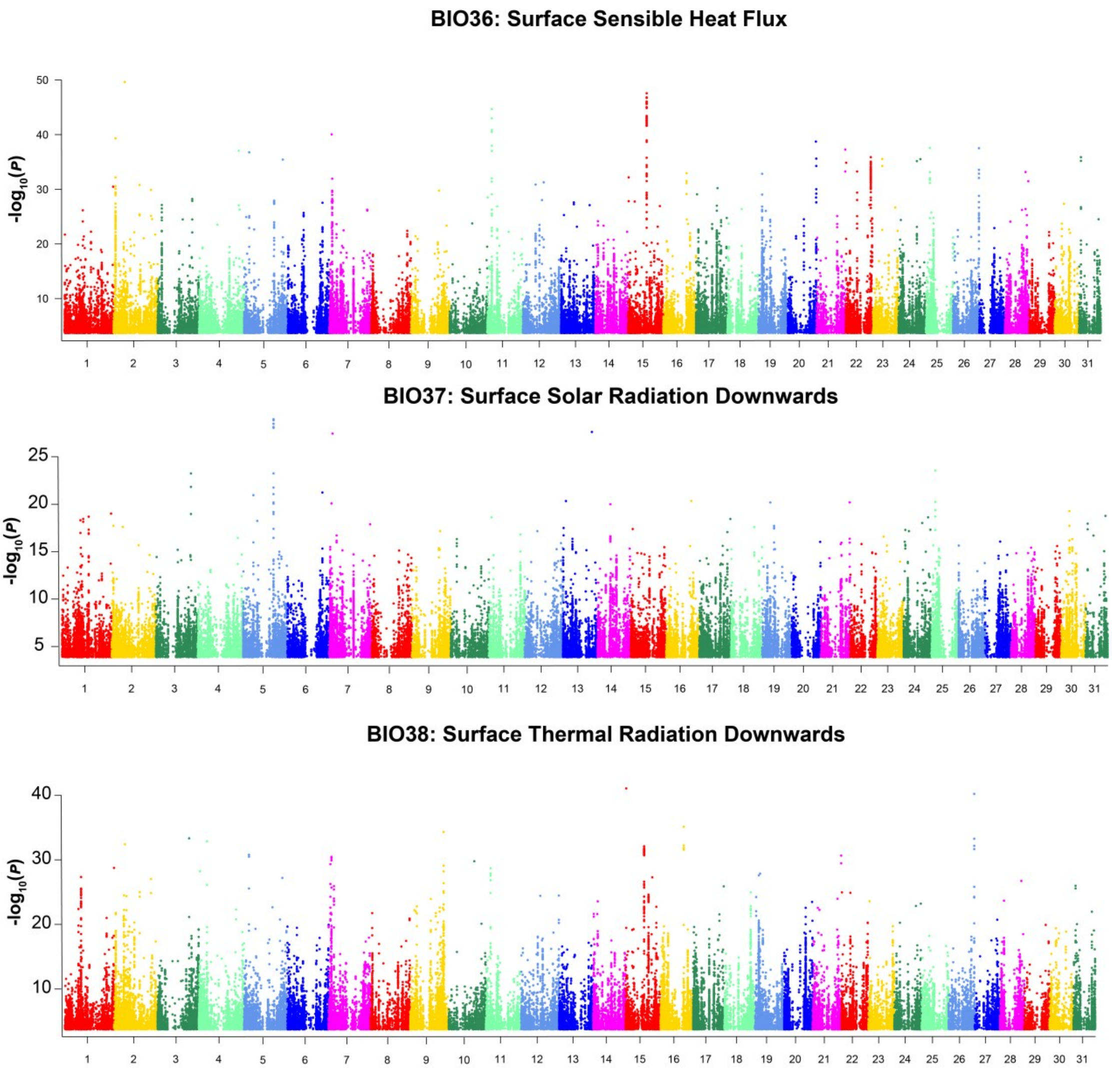

Supplementary Figure 14. Manhattan plot for SNPs associated with the 38 environmental factors. X and y axes are the chromosome number and significance of environmental factor-associated SNPs, respectively. The name of each environmental factor indicated on the top for each subpanel. Different chromosomes are shown in different colors.

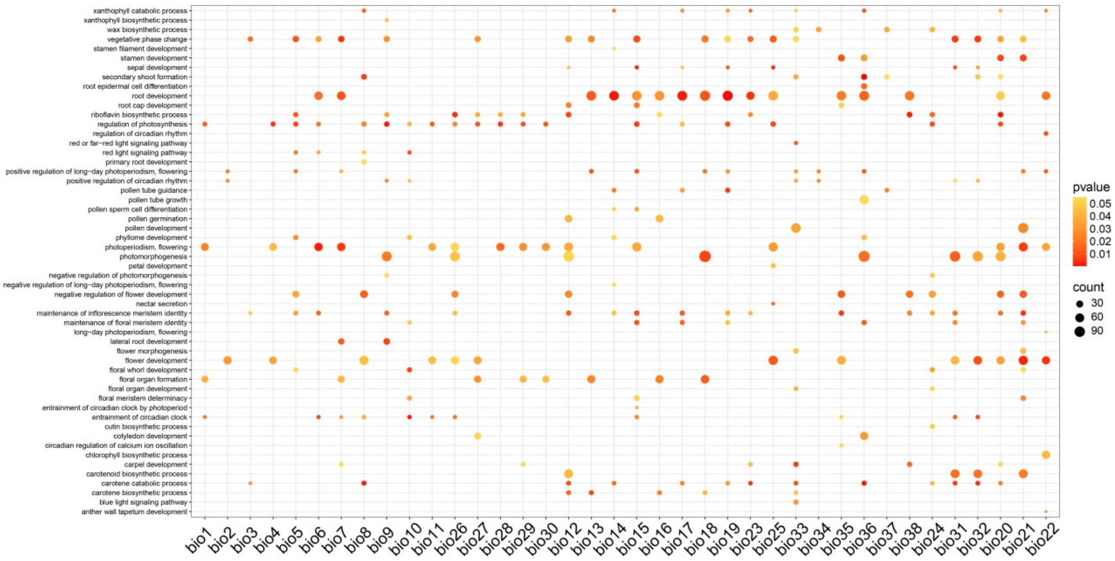

Supplementary Figure 15. Selective GO terms of the candidate genes that showed associations with the 38 environmental factors. Each column represents environmental factor associated GO terms. Circle size indicates the number of genes for each GO terms. Colors from orange to red are significant values. GO term IDs are shown on the y axes. Full GO terms of the 38 environmental factors associated genes were included in Supplementary Table 12.

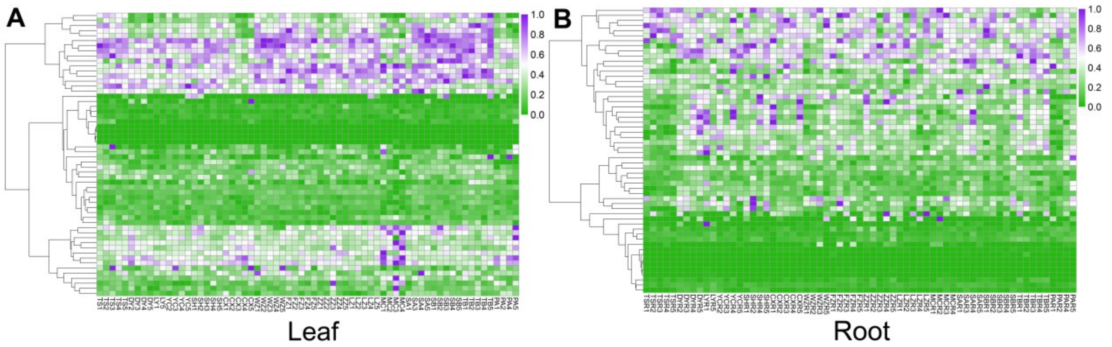

Supplementary Figure 16. Expression pattern of the flowering-related genes in leaf (**A**) and root (**B**) that showed high genetic differentiation between native US, low- and high-latitude Chinese populations.

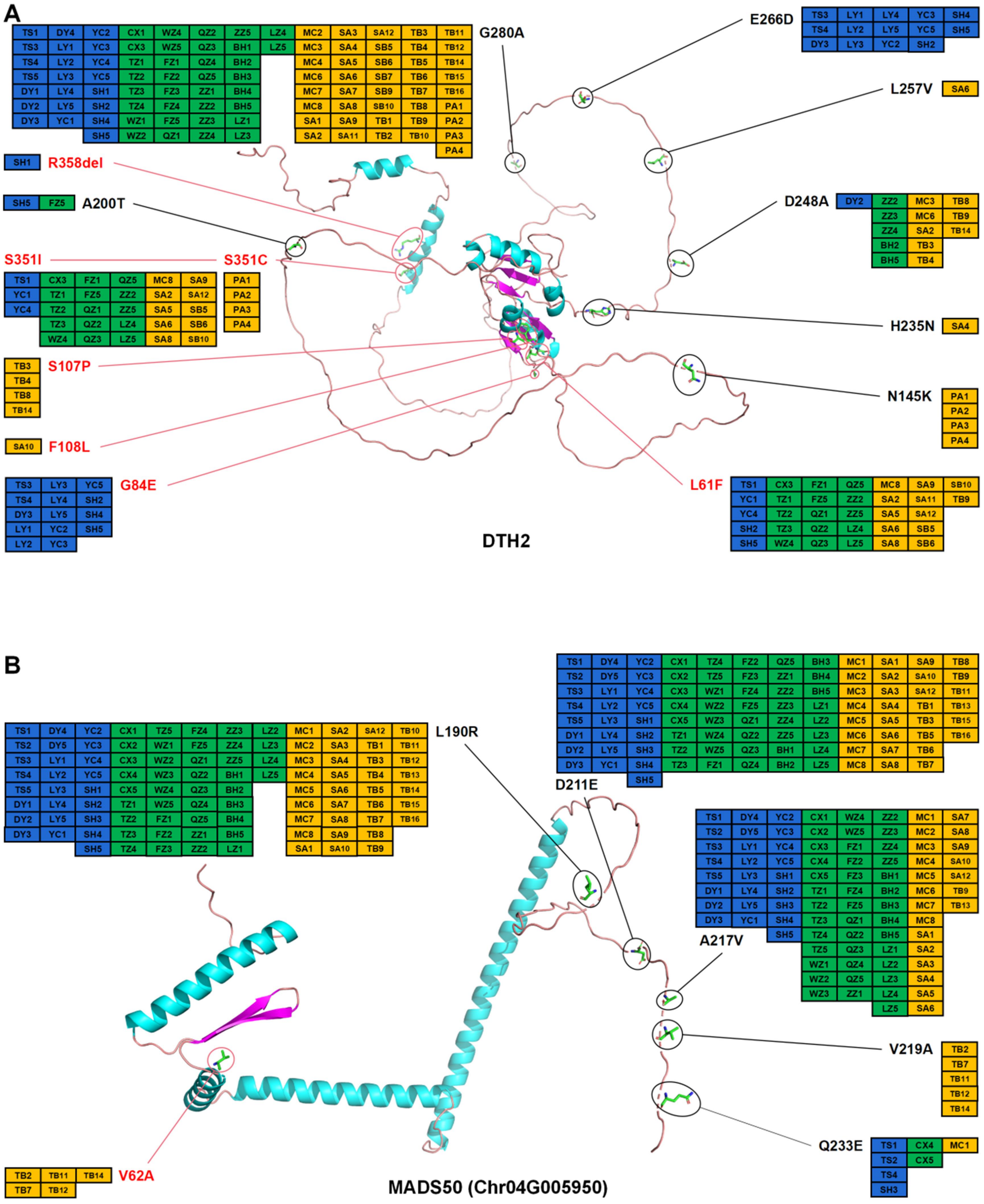
A
G280A
E266D
L257V
R358del
A200T
D248A
S351I
S351C
H235N
S107P
N145K
F108L
G84E
L61F
DTH2
B
L190R
D211E
A217V
V219A
Q233E
V62A
MADS50 (Chr04G005950)

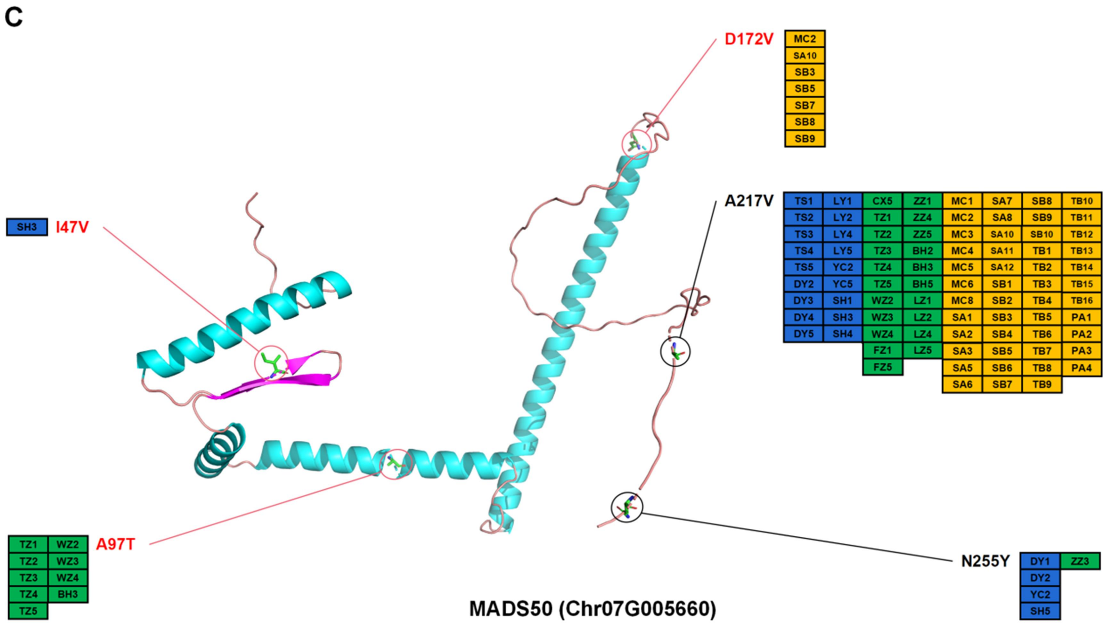
C
D172V
MC2 SA10 SB3 SB5 SB7 SB8 SB9
A217V
TS1 LY1 CX5 ZZ1 MC1 SA7 SB8 TB10
TS2 LY2 TZ1 ZZ4 MC2 SA8 SB9 TB11
TS3 LY4 TZ2 ZZ5 MC3 SA10 SB10 TB12
TS4 LY5 TZ3 BH2 MC4 SA11 TB1 TB13
TS5 YC2 TZ4 BH3 MC5 SA12 TB2 TB14
DY2 YC5 TZ5 BH5 MC6 SB1 TB3 TB15
DY3 SH1 WZ2 LZ1 MC8 SB2 TB4 TB16
DY4 SH3 WZ3 LZ2 SA1 SB3 TB5 PA1
DY5 SH4 WZ4 LZ4 SA2 SB4 TB6 PA2
FZ1 LZ5 SA3 SB5 TB7 PA3
FZ5 SA5 SB6 TB8 PA4
SA6 SB7 TB9
SH3
I47V
TZ1 WZ2
TZ2 WZ3
TZ3 WZ4
TZ4 BH3
TZ5
A97T
N255Y
DY1 ZZ3
DY2
YC2
SH5
MADS50 (Chr07G005660)

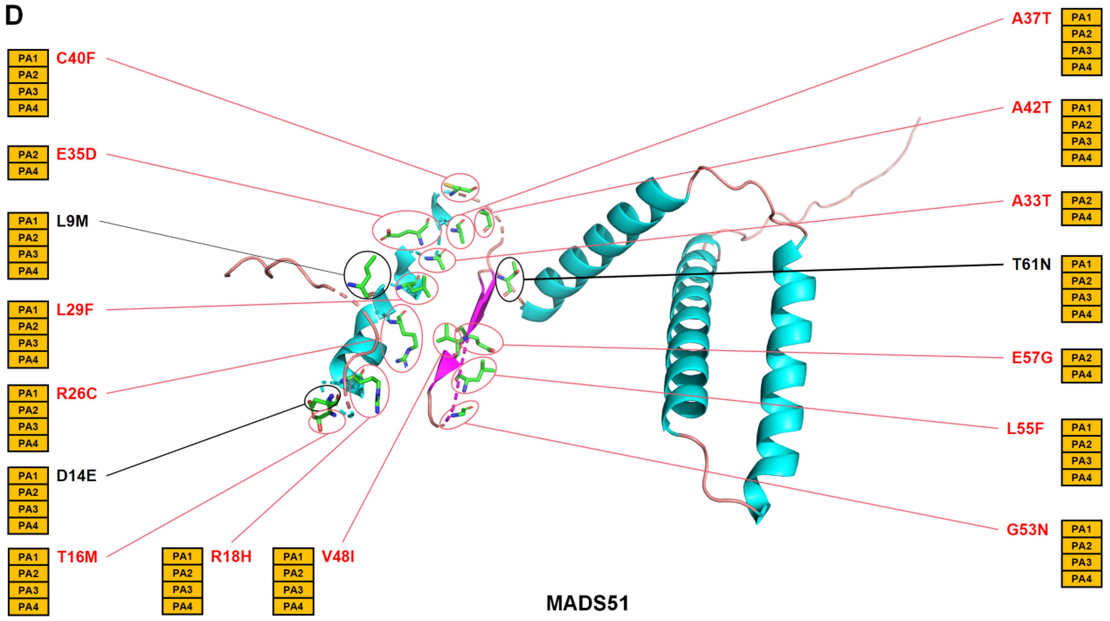
D
C40F
PA1 PA2 PA3 PA4
E35D
PA2 PA4
L9M
PA1 PA2 PA3 PA4
L29F
PA1 PA2 PA3 PA4
R26C
PA1 PA2 PA3 PA4
D14E
PA1 PA2 PA3 PA4
T16M
PA1 PA2 PA3 PA4
R18H
PA1 PA2 PA3 PA4
V48I
PA1 PA2 PA3 PA4
A37T
PA1 PA2 PA3 PA4
A42T
PA1 PA2 PA3 PA4
A33T
PA2 PA4
T61N
PA1 PA2 PA3 PA4
E57G
PA2 PA4
L55F
PA1 PA2 PA3 PA4
G53N
PA1 PA2 PA3 PA4
MADS51

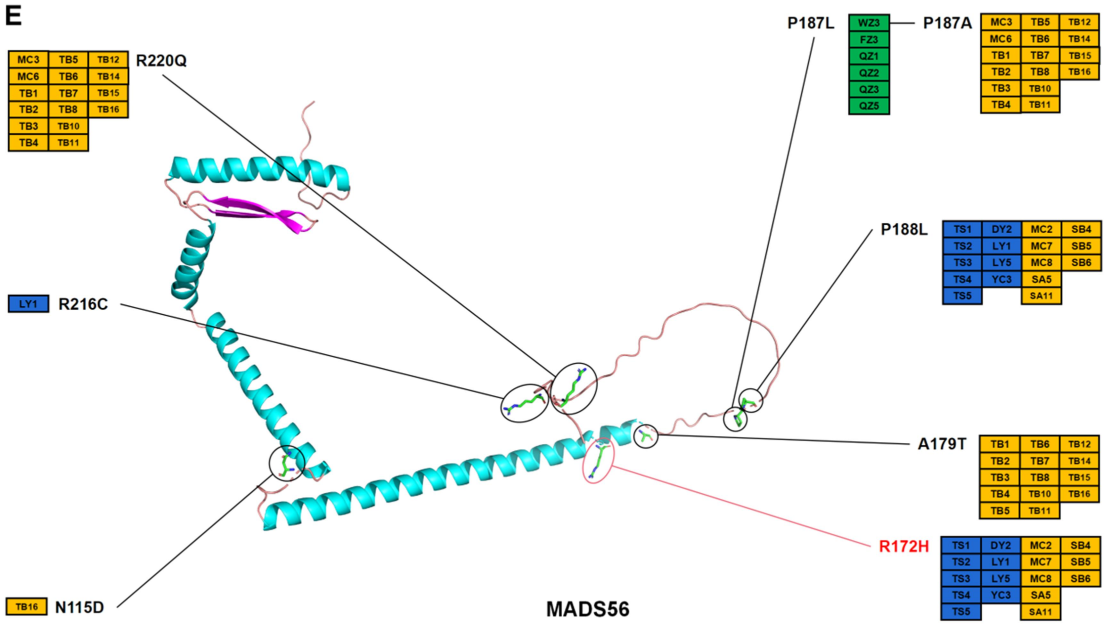

Supplementary Figure 17. Simulation of protein structure of the high divergence flowering genes. Non-synonymous mutations were indicated on left and right sides of each gene. Amino acid highlighted with red color indicated that these non-synonymous mutations lead to structural variation. Accessions numbers in blue, green and orange boxes represent high-, low-latitude Chinese and native US accessions, respectively.

Supplementary Table 1. Basic information of Pacbio and Nanopore long reads used in this study.

Parameter	PacBio reads	Nanopore reads
Reads number	71,810,411	9,935,868
Reads base (bp)	905,675,901,824	251,055,570,106
Reads N50 (bp)	13,929	34,411
Reads max (bp)	675,806	291,196

Supplementary Table 2. Features of the assembled genome of *S. alterniflora*.

Assembly	Parameter
Total length of scaffold (bp)	1,631,144,040
Scaffold number	763
Contig number	1,002
Scaffold N50 (bp)	50,761,989
Contig N50 (bp)	18,800,000
Scaffold max (bp)	73,030,954
Contig max (bp)	63,413,729
GC content (%)	44.95
Illumina reads mapping rate (%)	99.20
PacBio reads mapping rate (%)	99.95
CEGMA (%)	96.72
Complete and single-copy BUSCOs (%)	43.26
Complete and duplicated BUSCOs (%)	52.57
Fragmented BUSCOs (%)	0.42
Missing BUSCOs (%)	3.75
Hi-C reads (Gb)	251.49
Hi-C reads mapping rate (%)	97.73

Supplementary Table 3. Information of the protein-coding genes in *S. alterniflora* genome.

Annotation	Number
Number of protein-coding genes	73,711
Total length of protein-coding genes (bp)	282,156,950
Mean length of genes (bp)	3,827.88
Mean length of exons (bp)	1,432.95
Mean length of CDS (bp)	1,161.98
Mean length of intros (bp)	2,394.93
Number of pseudogenes	6,248
Number of miRNAs	233
Number of rRNAs	5,727
Number of tRNAs	978
GO_Annotation (%)	71.41
KEGG_Annotation (%)	33.53
KOG_Annotation (%)	50.19
TrEMBL_Annotation (%)	94.14
nr_Annotation (%)	94.04
All_Annotated (%)	94.32
